# Design, Synthesis, and Antifungal Structure‐Activity Relationship of Hydrazide Derivatives Containing a Cyclopropyl Group Against Phytopathogenic Fungi

**DOI:** 10.1002/cbdv.71820

**Published:** 2026-10-26

**Authors:** Shanshan Yang, Xinyuan Wang, Bixi Yin, Weiting Yan, Lei Wang, Yunying Sha, Lizhong Wang, Zhuyun Liu

**Affiliations:** ^1^ College of Intelligent Pharmaceutical and Chemical New Materials Taizhou Polytechnic College Taizhou P. R. China; ^2^ Yangtze River Pharmaceutical Group Taizhou P. R. China

**Keywords:** antifungal activity, cyclopropyl, hydrazide derivatives, phytopathogenic fungi, structure‐activity relationship

## Abstract

To discover novel antifungal agents with unique scaffolds for crop protection, a series of 23 hydrazide derivatives containing a cyclopropyl group, including 14 new chemical entities, were designed, synthesized, and evaluated for their antifungal activity against eight phytopathogenic fungi. The in vitro bioassay results at 50 µg/mL revealed that most target compounds exhibited moderate to excellent antifungal activities. Notably, three compounds (**4**, **6**, **7**) displayed the most potent broad‐spectrum activity with inhibition rates of 95.0%–100.0% against all eight tested fungi. Their EC_50_ values were below 10 µg/mL against most fungi. SEM analysis revealed that the parent compound **1** caused pronounced morphological alterations in *A. alternata* mycelia, including shrinkage, collapse, and irregular distortion, providing a preliminary indication of the cellular effect of the series. Molecular docking of compound **1** with succinate dehydrogenase (SDH) generated a plausible binding model, suggesting SDH as a potential target candidate for this series, pending direct enzymatic validation. SAR analysis demonstrated that the phenyl ring was consistently associated with high activity, and para‐substitution with halogen atoms is favorable for enhancing antifungal activity. These findings suggest that cyclopropyl‐containing hydrazide derivatives, particularly compounds **4**, **6**, and **7**, represent promising lead candidates for the development of novel antifungal agents.

## Graphical abstract

Twenty‐three cyclopropyl‐containing hydrazide derivatives, including 14 new chemical entities, are designed and synthesized. Compounds **4**, **6**, and **7** exhibit broad‐spectrum antifungal activity against eight phytopathogenic fungi with EC_50_ values below 10 µg/mL. SEM and molecular docking studies suggest disrupted fungal morphology and succinate dehydrogenase (SDH) as a potential target, highlighting these derivatives as promising antifungal lead candidates.

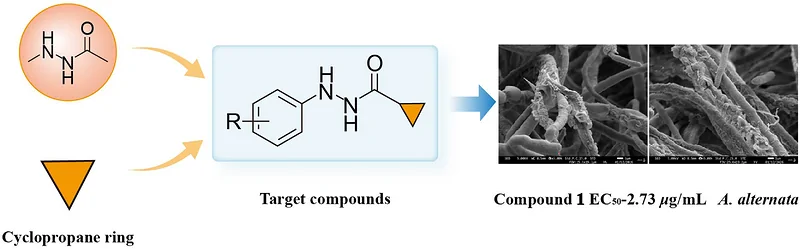

## 1 Introduction

It is estimated that up to 80% of plant diseases are caused by plant pathogenic fungi [1], which have seriously affected the yield and quality of crops, resulting in economic loss of 200 billion dollars every year worldwide [2, 3]. When plant pathogenic fungi infect crops, they also secrete some toxins and harmful metabolites. Some phytopathogenic fungi can also cause food safety problems due to these toxins, such as aflatoxin B1, a potent hepatocarcinogen [4, 5, 6, 7].

Although researchers have developed numerous solutions, including biological control, the cultivation of resistant plant varieties, and the application of biological pesticides, chemical fungicides remain the most effective measure for controlling plant pathogenic fungi [8, 9]. However, with the excessive and repeated use of chemical fungicides, fungal resistance is becoming increasingly serious [10]. In addition, some fungicides can be harmful to humans, beneficial insects, and microorganisms, and they can also influence the environment due to their slow degradation [1]. Therefore, it is necessary to develop new antifungal agents [11].

Hydrazide derivatives widely exist in natural and synthetic compounds, and are important pharmacophores in the field of drug and pesticide research and development. Hydrazide compounds have important application value in the development of new pesticides as a vital pharmacophore [12, 13, 14, 15]. Studies revealed that this class of compounds has diverse biological activities such as insecticidal [16], antibacterial [17], antifungal [18], as well as anticancer, antioxidant, and enzyme‐inhibitory activities [19, 20, 21, 22]. For instance, recent studies demonstrated that methoxy‐ and thiophene‐substituted benzodioxane hydrazone derivatives exhibited broad‐spectrum antibacterial, antifungal, anticancer, antioxidant, and enzyme‐inhibitory activities together with favorable pharmacokinetic profiles, and their terminal substituents play critical roles in modulating these biological activities [19, 20]. Indole‐acid‐based hydrazone derivatives also displayed potent antioxidant and cytoprotective effects via Nrf2‐mediated activation [21, 22]. These findings demonstrate that modification of the hydrazide/hydrazone scaffold can substantially influence biological activity and therapeutic potential. Up to now, many pesticides containing hydrazide structural units have been developed, such as Tebufenozide, Famoxadone, and Miexiuyihao (Figure 1) [23]. A series of N′‐phenylnicotinohydrazide and N′‐phenylisonicotinohydrazide derivatives bearing an aromatic acyl moiety have recently been reported as promising antifungal candidates [24, 25]. In those series, however, the acyl fragment was limited to aromatic carbonyl groups, and the influence of an aliphatic acyl moiety has not been explored.

**FIGURE 1:**
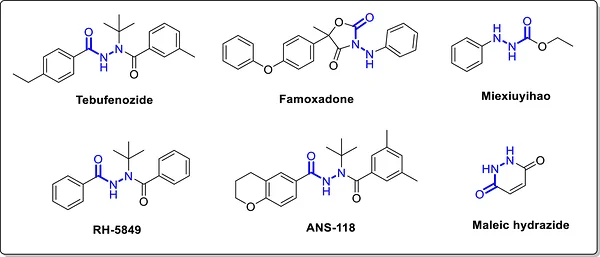
Pesticides containing hydrazide structural units.

The cyclopropane ring, the smallest cycloalkane, is widely used in synthetic molecules and secondary metabolites with biological activity, being an essential structural component [26]. Since August and his collaborators synthesized the first cyclopropane scaffold in 1882 [27], the field related to cyclopropane has received increasing attention [28], and many drugs containing a cyclopropyl group have been approved for marketing (Figure 2).

**FIGURE 2:**
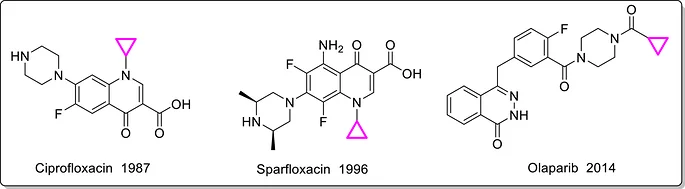
Marketed drugs containing a cyclopropyl group.

Many studies have demonstrated that the presence of cyclopropyl fragments is beneficial to the intrinsic lipophilicity, selectivity, and metabolic stability of drugs. In some cases, compounds can bind to target proteins through cyclopropyl groups, leading to increased activity and reduced off‐target effects [29, 30, 31]. However, to the best of our knowledge, hydrazide derivatives bearing a cyclopropylcarbonyl moiety have rarely been reported, and their antifungal activity against plant‐pathogenic fungi remains largely unexplored, which prompted us to undertake the present study.

Inspired by the above findings, a series of 23 hydrazide derivatives bearing a cyclopropyl group were designed and synthesized by adopting a molecular hybridization strategy to explore novel antifungal agents. The hydrazide pharmacophore and cyclopropyl fragment were selected as the privileged structures due to their well‐documented bioactivity profiles. The target compounds were evaluated for antifungal activity in vitro against phytopathogenic fungi, and the structure‐activity relationships (SARs) were systematically investigated. The potential antifungal mechanism was preliminarily elucidated through scanning electron microscopy and molecular docking studies.

## 2 Results and Discussion

### 2.1 Synthesis and Structure Characterization of Target Compounds

The synthesis route of compounds **1**‐**23** was shown in Scheme 1. Among them, compounds **1**‐**18** were prepared by reacting an activated intermediate generated from carbonyldiimidazole with substituted phenylhydrazine hydrochlorides. Compounds **19**‐**23** were synthesized by treating acyl chlorides with substituted anilines under alkaline conditions. The reaction conditions were simple and mild, the target compounds could be obtained in a short time with high yields, and the post‐treatment is convenient. Among the 23 target compounds, 14 compounds (**3**, **4**, **6**‐**16**, **18**) were novel structures reported for the first time, while 9 compounds (**1**, **2**, **5**, **17**, **19**‐**23**) were known compounds. All compound structures were confirmed by ^1^H NMR, ^13^C NMR, and HRMS analyses, with the corresponding spectra provided in Figures S1–S69.

**SCHEME 1:**
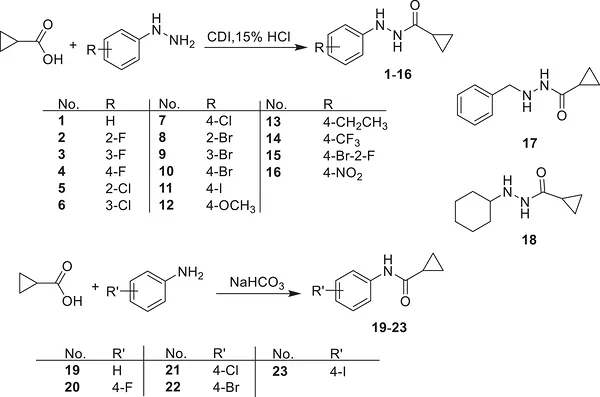
Synthesis route and structures of target compounds.

### 2.2 Antifungal Activity In Vitro

The in vitro antifungal activities of all target compounds were evaluated using the mycelial growth rate method at a concentration of 50 µg/mL, and the results were listed in Table 1.

**TABLE 1:** Antifungal activity of the target compounds against phytopathogenic fungi at 50 µg/mL.

No.	Inhibition rate (%) (mean ± SD, *n* = 3)							
	Fov	Fg	As	Ab	Aa	Cl	Cg	Po
**1**	82.5 ± 0.9	96.5 ± 0.9	100.0 ± 0.0	100.0 ± 0.0	100.0 ± 0.0	100.0 ± 0.0	100.0 ± 0.0	100.0 ± 0.0
**2**	70.6 ± 1.5	64.5 ± 1.7	92.7 ± 2.4	95.3 ± 0.0	96.8 ± 0.0	100.0 ± 0.0	91.5 ± 0.0	91.3 ± 0.0
**3**	95.4 ± 0.0	80.5 ± 3.0	100.0 ± 0.0	100.0 ± 0.0	96.8 ± 0.0	100.0 ± 0.0	100.0 ± 0.0	100.0 ± 0.0
**4**	100.0 ± 0.0	100.0 ± 0.0	100.0 ± 0.0	100.0 ± 0.0	96.8 ± 0.0	100.0 ± 0.0	100.0 ± 0.0	100.0 ± 0.0
**5**	45.9 ± 0.0	19.5 ± 1.7	87.1 ± 1.4	83.6 ± 0.0	90.8 ± 0.9	100.0 ± 0.0	80.9 ± 0.0	81.9 ± 2.5
**6**	100.0 ± 0.0	97.0 ± 0.0	95.2 ± 0.0	100.0 ± 0.0	96.8 ± 0.0	100.0 ± 0.0	100.0 ± 0.0	100.0 ± 0.0
**7**	95.4 ± 0.0	100.0 ± 0.0	95.2 ± 0.0	100.0 ± 0.0	100.0 ± 0.0	100.0 ± 0.0	100.0 ± 0.0	100.0 ± 0.0
**8**	45.9 ± 0.0	20.5 ± 0.0	83.1 ± 2.4	84.4 ± 1.4	84.3 ± 0.9	100.0 ± 0.0	68.8 ± 2.5	76.1 ± 0.0
**9**	82.5 ± 2.4	92.0 ± 0.9	87.1 ± 1.4	100.0 ± 0.0	93.5 ± 0.0	100.0 ± 0.0	89.4 ± 0.0	95.7 ± 0.0
**10**	82.0 ± 0.9	90.5 ± 0.9	95.2 ± 0.0	100.0 ± 0.0	88.6 ± 0.0	100.0 ± 0.0	93.6 ± 0.0	100.0 ± 0.0
**11**	52.1 ± 0.0	69.5 ± 1.7	95.2 ± 0.0	90.6 ± 2.3	91.9 ± 0.0	100.0 ± 0.0	80.1 ± 1.2	91.3 ± 0.0
**12**	66.5 ± 1.8	85.5 ± 1.7	100.0 ± 0.0	100.0 ± 0.0	95.1 ± 0.0	100.0 ± 0.0	89.4 ± 0.0	91.3 ± 0.0
**13**	78.8 ± 0.9	91.8 ± 0.0	95.7 ± 0.0	100.0 ± 0.0	93.3 ± 0.9	100.0 ± 0.0	100.0 ± 0.0	94.0 ± 0.0
**14**	69.3 ± 0.9	74.1 ± 1.2	93.6 ± 0.0	100.0 ± 0.0	94.4 ± 0.9	96.4 ± 0.0	79.2 ± 1.9	94.0 ± 0.0
**15**	68.8 ± 1.8	72.1 ± 3.1	95.7 ± 0.0	100.0 ± 0.0	98.5 ± 0.0	100.0 ± 0.0	83.6 ± 1.1	94.0 ± 0.0
**16**	27.5 ± 1.8	27.2 ± 2.4	46.8 ± 0.0	69.5 ± 2.5	59.5 ± 1.8	47.0 ± 1.2	26.4 ± 3.8	70.0 ± 2.0
**17**	6.9 ± 0.9	26.5 ± 2.0	36.2 ± 2.1	75.9 ± 1.2	20.5 ± 1.8	15.3 ± 0.7	26.4 ± 0.0	49.3 ± 1.2
**18**	20.1 ± 0.9	23.1 ± 2.4	32.6 ± 2.5	29.1 ± 3.3	21.5 ± 0.0	16.5 ± 0.7	25.8 ± 1.1	26.0 ± 2.0
**19**	30.7 ± 1.8	40.8 ± 0.0	55.3 ± 0.0	57.4 ± 2.1	50.8 ± 1.5	26.9 ± 0.7	45.3 ± 0.0	53.3 ± 2.3
**20**	34.9 ± 0.0	50.3 ± 1.2	45.4 ± 1.2	41.8 ± 1.2	28.7 ± 2.4	17.7 ± 1.4	34.0 ± 0.0	73.3 ± 1.2
**21**	49.7 ± 1.8	59.9 ± 1.2	52.5 ± 1.2	78.0 ± 1.2	51.8 ± 0.9	47.0 ± 1.2	67.3 ± 2.2	64.0 ± 2.0
**22**	52.4 ± 1.6	45.6 ± 4.2	56.7 ± 1.2	83.7 ± 1.2	62.6 ± 0.9	41.8 ± 1.4	75.5 ± 0.0	58.0 ± 2.0
**23**	34.9 ± 0.0	36.1 ± 1.2	39.7 ± 2.5	56.7 ± 2.5	62.6 ± 0.9	22.1 ± 0.7	23.3 ± 1.1	38.0 ± 3.5
TBZ	100.0 ± 0.0	100.0 ± 0.0	75.2 ± 1.0	60.6 ± 1.0	61.9 ± 0.0	38.1 ± 1.6	100.0 ± 0.0	58.4 ± 1.9
KXM	60.9 ± 1.9	73.7 ± 2.1	72.9 ± 3.8	100.0 ± 0.0	55.4 ± 0.0	37.8 ± 1.5	45.6 ± 0.0	50.3 ± 2.0

*Abbreviations*: Aa, *Alternaria alternata*; Ab, *Alternaria brassicae*; As, *Alternaria solani*; Cg, *Colletotrichum gloeosporioides*; Cl, *Curvularia lunata*; Fg, *Fusarium graminearum*; Fov, *Fusarium oxysporum* f. sp. vasinfectum; KXM, kresoxim‐methyl; Po, *Pyricularia oryzae*; TBZ, *Thiabendazole*.

As shown in Table 1, most compounds exhibited moderate to excellent inhibitory activities against all tested phytopathogenic fungi. Notably, seven compounds displayed outstanding broad‐spectrum activity. Compounds **4**, **6**, and **7** demonstrated the most potent efficacy, achieving inhibition rates of 95.0%–100.0% across all eight tested fungi. In particular, compound **4** exhibited almost 100.0% inhibition against every tested fungus, representing the most comprehensive antifungal profile in this series. Compounds **1**, **3**, **9**, and **10** also showed good broad‐spectrum activities, with inhibition rates exceeding 80.0% for most of the tested fungi. By contrast, compounds **17** and **18** displayed notably weak activities, with inhibition rates generally below 50.0% for most fungi.

Among the tested fungi, *C. lunata* was found to be the most sensitive target: 15 out of 23 compounds achieved inhibition rates of 100.0% or very close to it, markedly surpassing both positive controls TBZ (inhibition rate = 38.1%) and KXM (inhibition rate = 37.8%). Similarly, the target compounds also exhibited superior activity against *A. solani* and *P. oryzae*, with 15 and 18 compounds showing higher inhibition rates than TBZ (inhibition rate = 75.2%) and KXM (inhibition rate = 72.9%) for *A. solani*, respectively, and comparable advantages observed for *P. oryzae*. It is worth noting that even the least active compounds (**17**, **18**, and the amide series **19**‐**23**) retained moderate activity against certain fungi such as *F. graminearum* and *A. brassicae*, suggesting some degree of pathogen‐selective susceptibility.

In order to further evaluate the antifungal potential of the target compounds, based on the above results of the initial screening activity, we selected fifteen compounds (**1**‐**15**) with high inhibition rates (>80% against most of the tested fungi) and further determined their EC_50_ values against the eight fungi mentioned above (Table 2). In contrast, compounds **16**‐**18** and the amide series **19**‐**23** showed only weak activity in the primary screening (inhibition rates generally below 50%), and were therefore not subjected to EC_50_ determination; their qualitative data at 50 µg/mL already suffice to support the corresponding SAR conclusions.

**TABLE 2:** EC_50_ values (95% CI) of the target compounds against eight phytopathogenic fungi.

No.	EC_50_ (µg/mL)							
	Fov	Fg	As	Ab	Aa	Cl	Cg	Po
**1**	17.8 (13.9‐22.8)	9.36 (8.00‐11.0)	5.64 (5.04‐6.31)	3.73 (3.56‐3.91)	2.73 (2.33‐3.20)	3.85 (3.37‐4.40)	10.3 (7.50‐14.1)	3.92 (3.31‐4.64)
**2**	18.6 (16.2‐21.5)	27.7 (22.2‐34.6)	9.22 (7.09‐12.0)	8.04 (7.14‐9.05)	3.76 (3.24‐4.36)	3.51 (2.75‐4.48)	16.4 (12.0‐22.3)	8.72 (8.44‐9.00)
**3**	5.65 (4.54‐7.05)	14.8 (12.0‐18.3)	7.48 (6.24‐8.98)	5.40 (4.55‐6.40)	2.52 (2.28‐2.79)	5.66 (4.59‐6.98)	11.9 (10.2‐14.1)	5.86 (5.29‐6.50)
**4**	4.92 (4.44‐5.45)	4.56 (3.82‐5.45)	3.32 (2.77‐3.99)	2.60 (2.31‐2.94)	2.10 (1.85‐2.38)	1.15 (0.78‐1.71)	8.82 (6.57‐11.9)	3.36 (2.98‐3.79)
**5**	>50	>50	8.84 (8.13‐9.63)	12.1 (10.8‐13.7)	4.59 (3.77‐5.59)	3.34 (2.53‐4.40)	17.2 (13.5‐21.9)	12.8 (11.4‐14.3)
**6**	4.92 (4.21‐5.76)	4.37 (3.68‐5.19)	3.61 (2.79‐4.67)	3.44 (3.03‐3.90)	4.01 (3.54‐4.55)	3.86 (3.38‐4.42)	8.04 (7.08‐9.13)	5.24 (4.63‐5.93)
**7**	6.16 (5.83‐6.50)	5.06 (4.16‐6.17)	3.25 (2.96‐3.57)	4.07 (3.44‐4.81)	1.90 (1.65‐2.19)	2.38 (1.92‐2.94)	5.03 (4.22‐6.00)	3.50 (3.10‐3.95)
**8**	>50	>50	10.4 (9.82‐11.1)	14.3 (11.0‐18.6)	9.34 (7.86‐11.1)	3.77 (3.17‐4.48)	25.9 (19.6‐34.4)	18.6 (15.4‐22.4)
**9**	16.0 (13.3‐19.3)	8.65 (6.88‐10.9)	11.3 (8.99‐14.1)	8.58 (7.67‐9.60)	6.42 (5.23‐7.87)	4.14 (2.30‐7.48)	9.91 (8.31‐11.8)	6.40 (4.59‐8.94)
**10**	12.3 (11.5‐13.1)	6.67 (6.00‐7.43)	5.93 (5.29‐6.65)	5.44 (4.92‐6.02)	5.42 (4.42‐6.64)	3.95 (3.26‐4.78)	9.04 (7.74‐10.6)	5.34 (4.85‐5.87)
**11**	43.3 (26.8‐63.9)	18.6 (16.8‐20.5)	7.68 (5.44‐10.9)	7.59 (6.26‐9.20)	4.26 (3.00‐6.03)	3.78 (3.11‐4.59)	12.0 (11.0‐13.2)	5.36 (4.27‐6.74)
**12**	17.7 (12.6‐24.9)	9.12 (8.16‐10.2)	4.84 (4.04‐5.80)	5.52 (4.30‐7.09)	5.02 (3.74‐6.75)	4.00 (3.26‐4.91)	8.59 (7.81‐9.46)	7.05 (6.68‐7.45)
**13**	8.19 (6.37‐10.5)	5.91 (4.88‐7.17)	5.52 (4.73‐6.44)	5.32 (4.63‐6.11)	7.94 (6.19‐10.2)	3.91 (3.06‐4.98)	7.86 (6.94‐8.90)	5.79 (5.44‐6.16)
**14**	19.7 (17.4‐22.2)	13.8 (12.7‐15.0)	5.79 (4.53‐7.41)	5.59 (4.74‐6.60)	5.04 (4.12‐6.16)	4.56 (3.49‐5.96)	12.5 (10.6‐14.7)	8.89 (7.20‐11.0)
**15**	16.6 (14.4‐19.0)	17.1 (15.8‐18.6)	6.61 (5.35‐8.16)	5.40 (4.42‐6.61)	5.84 (5.00‐6.82)	4.11 (3.49‐4.84)	9.84 (7.73‐12.5)	8.35 (6.91‐10.1)
TBZ	4.23 (3.66‐4.91)	0.83 (0.80‐0.85)	21.3 (16.8‐26.9)	28.7 (23.8‐34.6)	27.1 (22.6‐32.3)	>50	1.03 (0.72‐1.47)	35.5 (30.5‐41.3)
KXM	23.1 (21.7‐24.5)	6.62 (5.87‐7.45)	13.5 (10.5‐17.3)	0.48 (0.39‐0.60)	36.3 (29.9‐44.1)	>50	>50	48.9 (42.7‐56.0)

*Abbreviations*: Aa, *Alternaria alternata*; Ab, *Alternaria brassicae*; As, *Alternaria solani*; Cg, *Colletotrichum gloeosporioides*; Cl, *Curvularia lunata*; Fg, *Fusarium graminearum*; Fov, *Fusarium oxysporum* f. sp. vasinfectum; KXM, kresoxim‐methyl; Po, *Pyricularia oryzae*; TBZ, *Thiabendazole*.

From the data in Table 2, we can see that the EC_50_ values of most compounds were below 10 µg/mL, indicating that they had certain inhibitory activities against various tested fungi. The comprehensive activities of the three compounds (**4**, **6**, and **7**) were relatively outstanding, with EC_50_ values below 10 µg/mL for all tested fungi, demonstrating a broad spectrum of antifungal potential. The comprehensive performance of compound **4** was particularly outstanding, with an EC_50_ value of 2.10 µg/mL against *A. alternata* and 1.15 µg/mL against *C. lunata*, the latter being far lower than those of the two positive controls (EC_50_> 50 µg/mL). Compound **4** exhibited potent antifungal activity against *C. lunata*, with an EC_50_ value of 1.15 µg/mL, which was substantially lower than those of the two positive controls (EC_50_> 50 µg/mL). Moreover, compound **4** also maintained good activity for *A. solani* (EC_50_ = 3.32 µg/mL) and *P. oryzae* (EC_50_ = 3.36 µg/mL).

Before comparing the target compounds with the reference fungicides, it should be noted that TBZ remained the most potent agent against *F. graminearum* (EC_50_ = 0.83 µg/mL) and *C. gloeosporioides* (EC_50_ = 1.03 µg/mL), and KXM against *A. brassicae* (EC_50_ = 0.48 µg/mL), values that none of the target compounds reached against these three fungi. Nevertheless, both TBZ and KXM showed markedly weaker activity against *A. solani*, *A. alternata*, *C. lunata*, and *P. oryzae*, fungi for which the target compounds offered a clear advantage, as detailed below. Among the eight tested fungi, TBZ and KXM exhibited weak inhibitory activity against *A. solani*, with EC_50_ values of 21.3 and 13.5 µg/mL, respectively. In contrast, the target compounds generally demonstrated potent antifungal activity, with nearly all compounds displaying lower EC_50_ values than both positive controls. Notably, compounds **4** (EC_50_ = 3.32 µg/mL) and **7** (EC_50_ = 3.25 µg/mL) exhibited the most prominent activity, with EC_50_ values approximately 1/6 that of TBZ and 1/4 that of KXM. For *A. alternata*, both TBZ (EC_50_ = 27.1 µg/mL) and KXM (EC_50_ = 36.3 µg/mL) showed poor inhibitory efficacy, with EC_50_ values exceeding 25 µg/mL. However, the target compounds exhibited markedly superior activity against this fungus. Compounds **4** (EC_50_ = 2.10 µg/mL) and **7** (EC_50_ = 1.90 µg/mL) displayed EC_50_ values below 3 µg/mL, representing approximately 1/13 and 1/19 of those of TBZ and KXM, respectively. For *C. lunata*, the target compounds generally maintained strong inhibitory effects. Compounds **4** (EC_50_ = 1.15 µg/mL), **7** (EC_50_ = 2.38 µg/mL), and **6** (EC_50_ = 3.86 µg/mL) all exhibited EC_50_ values below 5 µg/mL, far surpassing the two positive controls (EC_50_> 50 µg/mL). For *P. oryzae*, TBZ and KXM showed weak activity, with EC_50_ values of 35.5 and 48.9 µg/mL, respectively. The target compounds demonstrated significantly enhanced inhibitory activities, with compounds **4** (EC_50_ = 3.36 µg/mL), **7** (EC_50_ = 3.50 µg/mL), and **6** (EC_50_ = 5.24 µg/mL) all substantially outperforming both positive controls.

### 2.3 Structure‐Activity Relationship

Based on the results presented in Tables 1 and 2, several experimentally supported SARs can be summarized (Figure 3).

**FIGURE 3:**
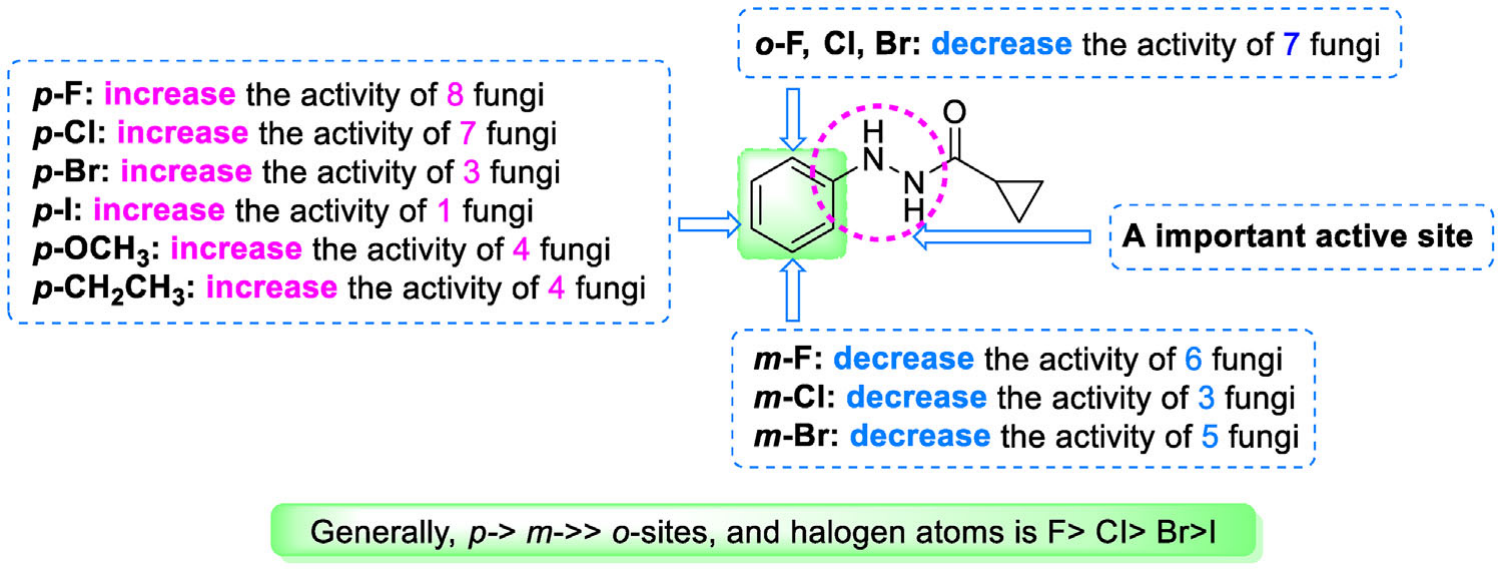
Structure‐activity relationships of the target compounds.

First, a phenyl ring directly attached to the hydrazide nitrogen is associated with high antifungal activity. Comparison of the phenyl‐bearing hydrazides with their nonaromatic analogues showed that replacing the phenyl ring with a benzyl (**17**) or cyclohexyl (**18**) group led to a sharp decline in inhibitory activity against all tested fungi; compounds **17** and **18** were consistently among the least active in the whole series. However, because these substitutions simultaneously alter aromaticity, linker length, and conformational properties, their individual contributions cannot be decoupled from the present dataset. Notably, the benzyl analogue **17** retains an aromatic ring (separated from the hydrazide nitrogen by only a methylene spacer), yet still displays very weak activity, suggesting that direct attachment of the ring, rather than aromaticity per se, may be the more relevant feature.

Second, the type of substituent on the phenyl ring exerts a marked influence on antifungal activity. Halogen substituents are generally beneficial, with the fluoro compounds (**2**‐**4**) conferring the highest activity, followed by the chloro (**5**‐**7**) and bromo (**8**‐**10**) compounds, whereas iodo substitution (**11**) resulted in a significant reduction in potency. In contrast, strong electron‐withdrawing groups were notably detrimental: introduction of NO_2_ (**16**) or CF_3_ (**14**) led to a marked decrease in antifungal efficacy.

Third, the position of the substituent on the phenyl ring plays a crucial role. For the halogenated pairs (**2**‐**4**, **5**‐**7**, and **8**‐**10**), the para isomers (**4**, **7**, **10**) consistently outperformed their ortho counterparts (**2**, **5**, **8**), and the para‐halogen compounds **4**, **7**, and **10** showed the highest broad‐spectrum activity in the whole series; compound **13** (4‐ethyl) was likewise effective. These observations indicate that the para position is the preferred site for introducing activity‐enhancing substituents.

Fourth, the hydrazide N‐N linker is critical for potent activity. Compared with their corresponding hydrazide analogues (**19** vs. **1**, **20** vs. **4**, **21** vs. **7**, **22** vs. **10**, and **23** vs. **11**), the amide derivatives **19**‐**23** exhibited markedly reduced antifungal activity across all tested fungi.

### 2.4 SEM Analysis

To obtain a preliminary, morphological indication of the cellular effect of this series, SEM was performed on *A. alternata* hyphae treated with the parent compound **1** (3 µg/mL), which was selected as the template compound, in line with its role in the docking study. The results were shown in Figure 4.

**FIGURE 4:**
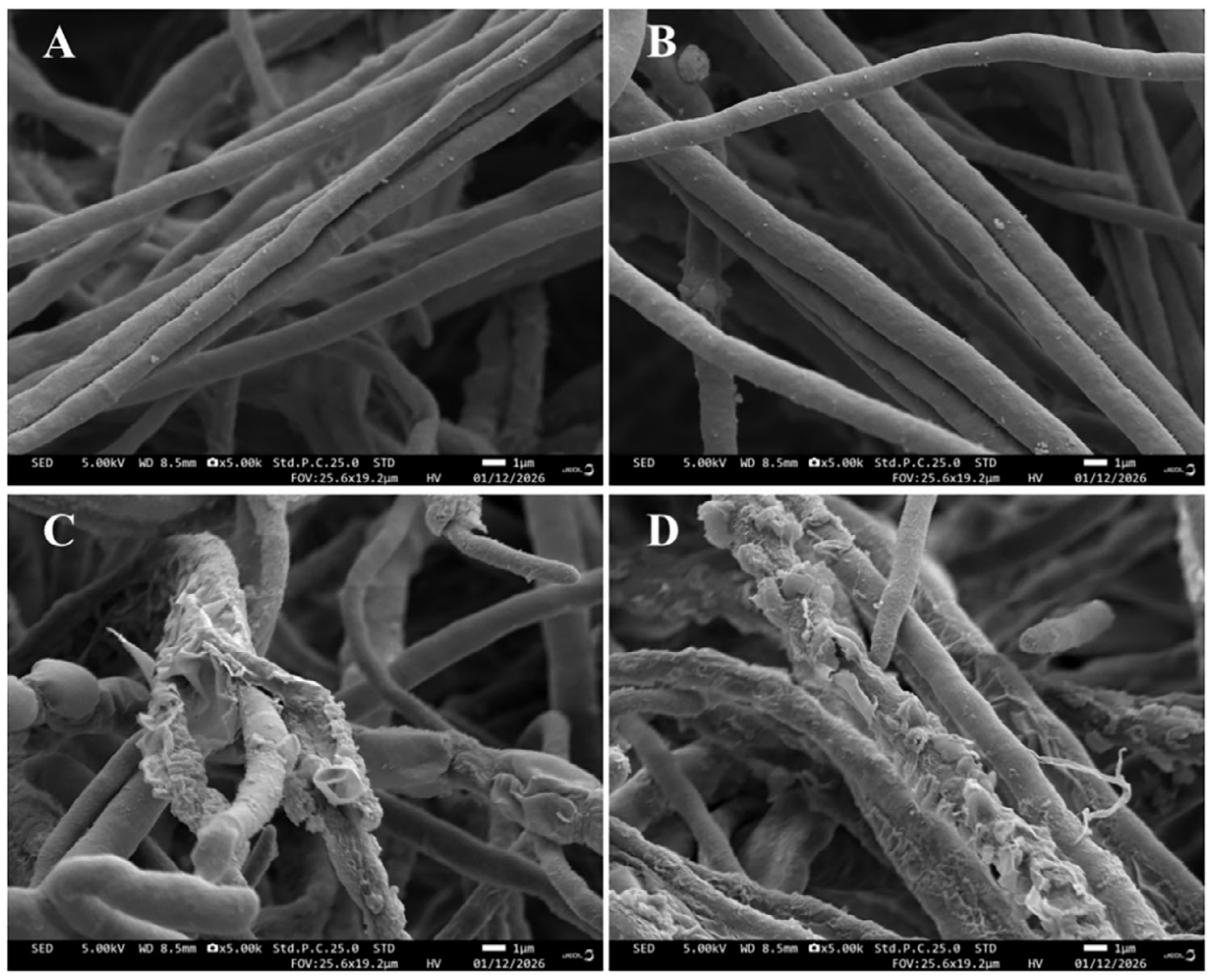
Analysis of hyphal morphology of *A. alternate* treated with **1** at 3 µg/mL. (A, B) Control group and (C, D) treated group.

In the control group, the hyphae exhibited a uniform, regular, and intact morphology. The hyphal surfaces were smooth and full, with consistent diameters and straight, linear growth patterns, showing no visible signs of shrinkage, distortion, or collapse (Figure 4A,B). By contrast, the hyphae treated with compound **1** underwent severe and extensive morphological alterations. Treatment with compound **1** caused a pronounced loss of structural integrity in the hyphae (Figure 4C,D), characterized by rough, shriveled, and irregularly wrinkled surfaces, together with evident distortion, twisting, and partial fragmentation. In addition, some hyphae appeared flattened and collapsed, suggesting disruption of the cell wall structure and possible leakage of intracellular contents. These pronounced ultrastructural damages are consistent with an inhibitory effect of compound **1** on mycelial growth and are suggestive of compromised cell wall integrity and membrane stability, although the underlying mechanism remains to be established.

### 2.5 Molecular Docking Analysis

The antifungal activity evaluation revealed that the cyclopropylhydrazides designed in this study exhibited significant broad‐spectrum antifungal activity. Moreover, the target compounds possess structural fragments similar to the carboxamide scaffold, a common structural motif found in succinate dehydrogenase inhibitors (SDHIs) [24]. To gain preliminary insight into the possible molecular basis of this activity, the parent compound **1—**which shares the same pharmacophoric scaffold (a phenyl ring directly attached to the hydrazide nitrogen) as the most potent analogues **4**, **6**, and **7—**was selected as a representative for molecular docking against SDH (Figure 5A). The results in Figure 5 show that compound **1** binds well to the ubiquinone binding site of SDH, primarily through three hydrogen bonds (Arg469‐N, 3.23 Å; Arg469‐N, 3.22 Å; and Asn53‐O, 3.16 Å), one π–cation interaction (Arg469, 4.11 Å), one π–alkyl interaction (Arg218, 4.99 Å), and other van der Waals forces. The calculated binding energy of this conformation was −6.779 kcal/mol, indicating that compound **1** binds to SDH with moderate to strong affinity. Given that compounds **4**, **6**, and **7** share the same pharmacophoric scaffold as compound **1** and differ only in the type and position of the halogen substituent, it is plausible that they bind to SDH in a similar orientation, with their higher antifungal potency possibly arising from substituent‐induced modulation of the interaction. As docking represents a computational prediction rather than experimental evidence, these in silico results are presented here as a preliminary hypothesis that SDH may serve as a plausible candidate target of this chemical series; direct enzymatic verification on compounds **1**, **4**, **6**, and **7** would be required to confirm this and is planned for future work.

**FIGURE 5:**
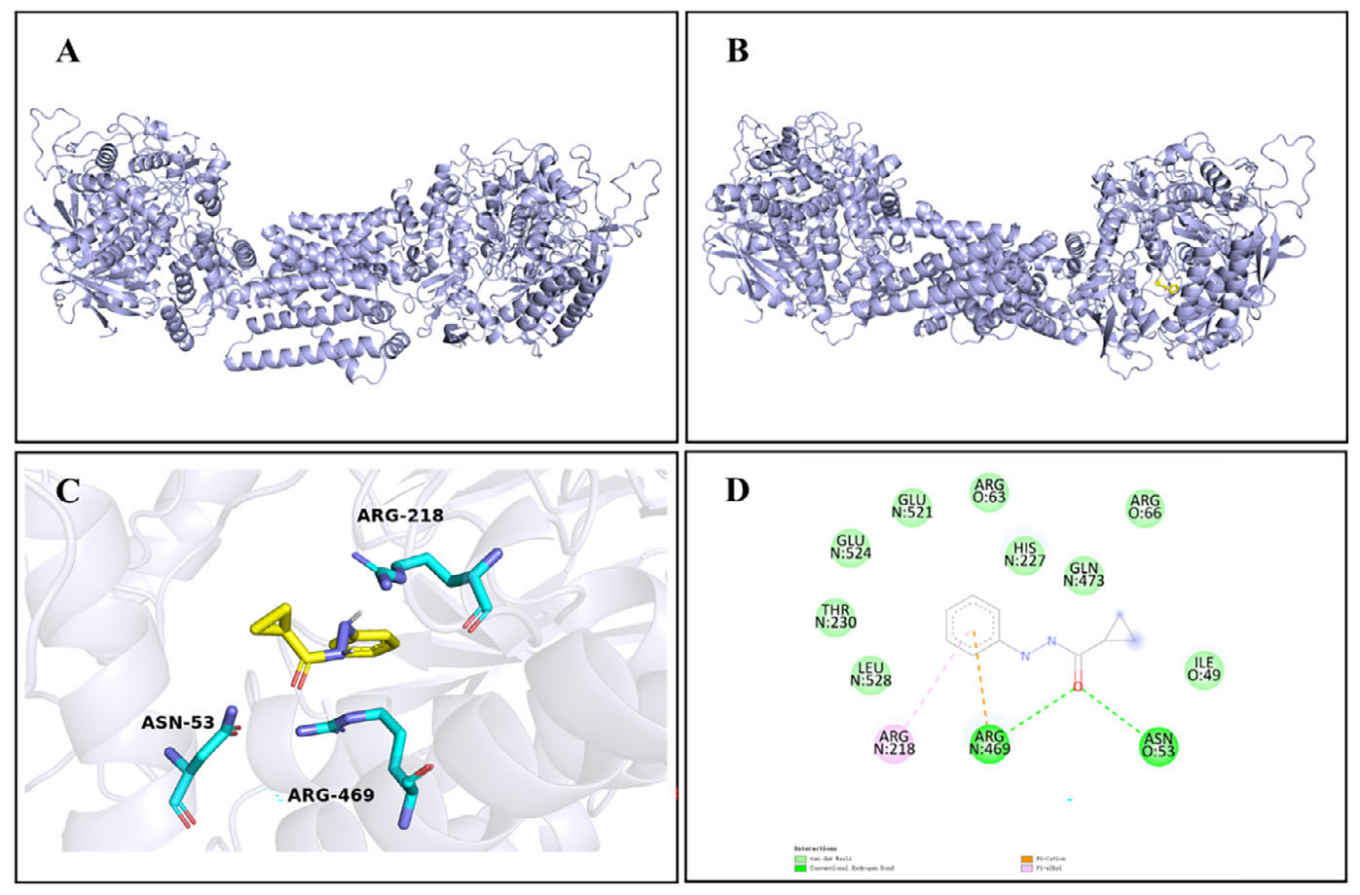
Binding mode of the compound **1** in the active site of SDH (PDB code: 2FBW).

It should be noted that the docking results presented here are computational predictions and are intended to provide a working hypothesis for the potential mode of action, rather than experimental proof of target engagement. Direct biochemical assays, such as SDH enzyme activity inhibition tests, are necessary to establish a causal link between SDH binding and the observed antifungal effects.

## 3 Conclusions

In summary, a series of 23 cyclopropyl‐containing hydrazide derivatives were designed, synthesized, and evaluated for their antifungal activity against eight phytopathogenic fungi. The bioassay results demonstrated that most target compounds showed moderate to excellent antifungal activity at 50 µg/mL. Among them, compounds **4** (4‐F), **6** (3‐Cl), and **7** (4‐Cl) exhibited the most potent efficacy, with inhibition rates of 95.0%–100.0% against all tested fungi and EC_50_ values below 10 µg/mL for most pathogens. In particular, compound **4** showed remarkable activity against *C. lunata* with an EC_50_ value of 1.15 µg/mL, far superior to both positive controls. SEM observations showed that the parent compound **1** induced pronounced mycelial morphological damage in *A. alternata*, providing morphological support for the cellular effect of this chemical series. Molecular docking of the parent hydrazide (compound **1**) provided a structural hypothesis suggesting SDH as a potential candidate target for this chemical class. Together with the observed morphological damage, this in silico finding offers a foundation for future target identification and mode‐of‐action studies. SAR analysis revealed that ring the phenyl ring was a critical structural feature for high activity, and para‐halogen substitution is the optimal modification strategy. Overall, this study highlights the potential of cyclopropyl‐containing hydrazide derivatives as novel antifungal lead compounds and provides valuable insights for further structural optimization.

## 4 Materials and Methods

### 4.1 Chemicals and Instruments

All reagents are of chemical purity grade, purchased from Aladdin or Sigma‐Aldrich and used without further purification. All chemical reactions were monitored by thin‐layer chromatography (TLC) on GF‐254, using a mixture of petroleum ether and ethyl acetate as the solvent system. The melting point was determined using an X‐4 binocular microscope melting point apparatus. ^1^H NMR and ^1^ ^3^C NMR spectra were recorded on a Bruker Avance 400/600 MHz spectrometer (Bruker, Germany) at room temperature, using DMSO‐*d*_6_ as the solvent and tetramethylsilane (TMS) as the internal standard. Chemical shifts (δ) are reported in parts per million (ppm), and coupling constants (*J*) in hertz (Hz).

### 4.2 General Synthesis of Compounds **1**‐**18**

Cyclopropanecarboxylic acid (0.861 g, 10 mmol, 1.0 equiv), 1,1′‐carbonyldiimidazole (CDI, 2.432 g, 15 mmol, 1.5 equiv), and 0.5 mL of 15% hydrochloric acid (w/v) were placed in a mortar and ground thoroughly until gas evolution ceased, indicating completion of the activation step. After the reaction, the mixture was extracted with ethyl acetate. The organic layer was dried over anhydrous Na_2_SO_4_ and concentrated under reduced pressure to afford the crude acyl‐CDI active intermediate. Subsequent reaction of this intermediate with the corresponding substituted phenylhydrazine hydrochlorides, followed by recrystallization from ethanol, gave the target compounds **1**‐**18** [32, 33]. Compounds **1**, **2**, **5**, **17**, and **19**‐**23** have been previously reported; their ^1^H NMR, ^13^C NMR, and HRMS data were in good agreement with the literature, except for compounds **2**, **5**, and **17**, for which no reference spectra were available and which were therefore fully characterized in this study. All spectra are provided in the Supporting Information.

#### 4.2.1 N'‐phenylcyclopropylhydrazide (1)

Light yellow crystal; yield: 75%; m.p.: 157.3°C–158.5°C. The ^1^H NMR, ^1^ ^3^C NMR, and HRMS data were basically consistent with the data of literature [34].

#### 4.2.2 N'‐(2‐fluorophenyl)cyclopropylhydrazide (2)

White crystal; yield: 71%; m.p.: 134.6°C–135.8°C; ^1^H NMR (400 MHz, DMSO‐*d*_6_) δ 9.91 (d, *J* = 2.0 Hz, 1H, NH‐CO), 7.60 (s, 1H, NH‐Ar), 7.11 – 6.96 (m, 2H, Ar‐H), 6.79 – 6.68 (m, 2H, Ar‐H), 1.67 (tt, *J* = 7.6, 4.9 Hz, 1H, cyclopropyl CH), 0.74 (ddt, *J* = 9.6, 4.7, 2.6 Hz, 4H, cyclopropyl CH_2_). ^13^C NMR (101 MHz, DMSO‐*d*_6_) δ 173.26, 151.75, 149.37, 137.50 (d, *J* = 11.11 Hz), 124.98, 119.00 (d, *J* = 7.07 Hz), 115.26 (d, *J* = 8.18 Hz), 113.91 (d, *J* = 4.04 Hz), 12.44, 6.82. HRMS (positive ESI) m/z: calcd for C_10_H_12_FN_2_O^+^ [M + H]^+^, 195.0928; found, 195.0923.

#### 4.2.3 N'‐(3‐fluorophenyl)cyclopropylhydrazide (3)

Yellow crystal; yield: 69%; m.p.: 151.0°C–152.3°C; ^1^H NMR (400 MHz, DMSO‐*d*_6_) δ 9.89 (d, *J* = 2.3 Hz, 1H, NH‐CO), 8.00 (d, *J* = 2.1 Hz, 1H, NH‐Ar), 7.15 (td, *J* = 8.1, 6.9 Hz, 1H, Ar‐H), 6.54 – 6.38 (m, 3H, Ar‐H), 1.65 (tt, *J* = 7.7, 4.8 Hz, 1H, cyclopropyl CH), 0.74 (ddt, *J* = 11.9, 4.8, 2.6 Hz, 4H, cyclopropyl CH_2_). ^13^C NMR (101 MHz, DMSO‐*d*_6_) δ 173.24, 163.65 (d, *J* = 241.39 Hz), 152.26, 152.16, 130.77 (d, *J* = 10.1 Hz), 108.49, 104.84 (d, *J* = 22.22 Hz), 98.89(d, *J* = 26.26 Hz), 12.41, 6.84. HRMS (positive ESI) m/z: calcd for C_10_H_12_FN_2_O^+^ [M + H]^+^, 195.0928; found, 195.0922.

#### 4.2.4 N'‐(4‐fluorophenyl)cyclopropylhydrazide (4)

Yellow crystal; yield: 74%; m.p.: 138.0°C–139.3°C; ^1^H NMR (400 MHz, DMSO‐*d*_6_) δ 9.86 (d, *J* = 2.9 Hz, 1H, NH‐CO), 7.66 (d, *J* = 2.9 Hz, 1H, NH‐Ar), 7.09 – 6.93 (m, 2H, Ar‐H), 6.77 – 6.65 (m, 2H, Ar‐H), 1.65 (ddd, *J* = 7.7, 4.7, 2.8 Hz, 1H, cyclopropyl CH), 0.79 – 0.63 (m, 4H, cyclopropyl CH_2_). ^13^C NMR (101 MHz, DMSO‐*d*_6_) δ 173.21, 156.23 (*J* = 234.32 Hz), 146.51, 115.57 (*J* = 22.22 Hz), 113.69(*J* = 8.08 Hz), 12.44, 6.76. HRMS (positive ESI) m/z: calcd for C_10_H_12_FN_2_O^+^ [M + H]^+^, 195.0928; found, 195.0922.

#### 4.2.5 N'‐(2‐chlorophenyl)cyclopropylhydrazide (5)

Yellow crystal; yield: 68%; m.p.: 149.1°C–150.7°C; ^1^H NMR (400 MHz, DMSO‐*d*_6_) δ 10.02 (d, *J* = 2.4 Hz, 1H, NH‐CO), 7.40 (d, *J* = 2.3 Hz, 1H, NH‐Ar), 7.28 (dd, *J* = 7.7, 1.4 Hz, 1H, Ar‐H), 7.20 – 7.14 (m, 1H, Ar‐H), 6.78 – 6.72 (m, 2H, Ar‐H), 1.70 (tt, *J* = 7.7, 4.9 Hz, 1H, cyclopropyl CH), 0.76 (tt, *J* = 7.8, 2.8 Hz, 4H, cyclopropyl CH_2_). ^13^C NMR (101 MHz, DMSO‐*d*_6_) δ 173.19, 145.29, 129.59, 128.20, 119.84, 117.57, 113.26, 12.47, 6.91. HRMS (positive ESI) m/z: calcd for C_10_H_12_ClN_2_O^+^ [M + H]^+^, 211.0633(^35^Cl), 213.0603(^37^Cl); found, 211.0624, 213.0593.

#### 4.2.6 N'‐(3‐chlorophenyl)cyclopropylhydrazide (6)

Light yellow crystal; yield: 65%; m.p.: 138.9°C–140.8°C; ^1^H NMR (400 MHz, DMSO‐*d*_6_) δ 9.90 (d, *J* = 2.6 Hz, 1H, NH‐CO), 8.01 (d, *J* = 2.6 Hz, 1H, NH‐Ar), 7.15 (t, *J* = 8.0 Hz, 1H, Ar‐H), 6.73 – 6.61 (m, 3H, Ar‐H), 1.65 (tt, *J* = 7.7, 4.7 Hz, 1H, cyclopropyl CH), 0.74 (ddt, *J* = 14.7, 9.6, 3.8 Hz, 4H, cyclopropyl CH_2_). ^13^C NMR (101 MHz, DMSO‐*d*_6_) δ 173.26, 151.57, 133.93, 130.85, 118.23, 111.70, 111.13, 12.42, 6.88. HRMS (positive ESI) m/z: calcd for C_10_H_12_ClN_2_O^+^ [M + H]^+^, 211.0633(^35^Cl), 213.0603(^37^Cl); found, 211.0625, 213.0593.

#### 4.2.7 N'‐(4‐chlorophenyl)cyclopropylhydrazide (7)

White crystal; yield: 73%; m.p.: 142.6°C–144.1°C; ^1^H NMR (400 MHz, DMSO‐*d*_6_) δ 9.88 (d, *J* = 2.7 Hz, 1H, NH‐CO), 7.88 (d, *J* = 2.7 Hz, 1H, NH‐Ar), 7.19 – 7.15 (m, 2H, Ar‐H), 6.69 (d, *J* = 8.8 Hz, 2H, Ar‐H), 1.64 (tt, *J* = 7.8, 4.7 Hz, 1H, cyclopropyl CH), 0.76 – 0.69 (m, 4H, cyclopropyl CH_2_). ^13^C NMR (101 MHz, DMSO‐*d*_6_) δ 173.20, 148.94, 128.95, 122.08, 113.96, 12.42, 6.83. HRMS (positive ESI) m/z: calcd for C_10_H_12_ClN_2_O^+^ [M + H]^+^, 211.0633(^35^Cl), 213.0603(^37^Cl); found, 211.0625, 213.0594.

#### 4.2.8 N'‐(2‐bromophenyl)cyclopropylhydrazide (8)

Light yellow crystal; yield: 68%; m.p.: 135.2°C–136.9°C; ^1^H NMR (600 MHz, DMSO‐*d*_6_) δ 10.06 (d, *J* = 2.4 Hz, 1H, NH‐CO), 7.43 (dd, *J* = 7.9, 1.4 Hz, 1H, Ar‐H), 7.23 – 7.18 (m, 2H, NH‐Ar), 6.74 – 6.67 (m, 2H, Ar‐H), 1.67 (tt, *J* = 7.9, 4.7 Hz, 1H, cyclopropyl CH), 0.79 – 0.72 (m, 4H, cyclopropyl CH_2_). ^13^C NMR (151 MHz, DMSO‐*d*_6_) δ 173.13, 146.23, 132.80, 128.84, 120.56, 113.51, 107.33, 12.48, 6.94. HRMS (positive ESI) m/z: calcd for C_10_H_12_BrN_2_O^+^ [M + H]^+^, 255.0128.(^79^Br), 257.0107(^81^Br); found, 255.0118, 257.0096.

#### 4.2.9 N'‐(3‐bromophenyl)cyclopropylhydrazide (9)

Yellow crystal; yield: 60%; m.p.: 149.9°C–151.2°C; ^1^H NMR (600 MHz, DMSO‐*d*_6_) δ 9.91 (d, *J* = 2.7 Hz, 1H, NH‐CO), 8.01 (d, *J* = 2.5 Hz, 1H, NH‐Ar), 7.08 (t, *J* = 8.0 Hz, 1H, Ar‐H), 6.84 (dd, *J* = 7.7, 1.9 Hz, 1H, Ar‐H), 6.80 (t, *J* = 2.1 Hz, 1H, Ar‐H), 6.66 (dd, *J* = 8.3, 2.2 Hz, 1H, Ar‐H), 1.64 (tt, *J* = 8.1, 4.7 Hz, 1H, cyclopropyl CH), 0.77 – 0.69 (m, 4H, cyclopropyl CH_2_). ^13^C NMR (151 MHz, DMSO‐*d*_6_) δ 173.26, 151.69, 131.21, 122.51, 121.12, 114.56, 111.49, 12.42, 6.90. HRMS (positive ESI) m/z: calcd for C_10_H_12_BrN_2_O^+^ [M + H]^+^, 255.0128.(^79^Br), 257.0107(^81^Br); found, 255.0117, 257.0095.

#### 4.2.10 N'‐(4‐bromophenyl)cyclopropylhydrazide (10)

Light yellow crystal; yield: 71%; m.p.: 142.5°C–144.3°C; ^1^H NMR (400 MHz, DMSO‐*d*_6_) δ 9.88 (d, *J* = 2.6 Hz, 1H, NH‐CO), 7.90 (d, *J* = 2.6 Hz, 1H, NH‐Ar), 7.31 – 7.27 (m, 2H, Ar‐H), 6.67 – 6.63 (m, 2H, Ar‐H), 1.64 (tt, *J* = 7.7, 4.8 Hz, 1H, cyclopropyl CH), 0.73 (ddd, *J* = 10.2, 5.1, 2.8 Hz, 4H, cyclopropyl CH_2_). ^13^C NMR (101 MHz, DMSO‐*d*_6_) δ 173.21, 149.34, 131.78, 114.49, 109.59, 12.43, 6.85. HRMS (positive ESI) m/z: calcd for C_10_H_12_BrN_2_O^+^ [M + H]^+^, 255.0128.(^79^Br), 257.0107(^81^Br); found, 255.0121, 257.0099.

#### 4.2.11 N'‐(4‐iodophenyl)cyclopropylhydrazide (11)

Light yellow crystal; yield: 67%; m.p.: 162.7°C–164.4°C; ^1^H NMR (400 MHz, DMSO‐*d*_6_) δ 9.87 (d, *J* = 2.6 Hz, 1H, NH‐CO), 7.89 (d, *J* = 2.6 Hz, 1H, NH‐Ar), 7.45 – 7.41 (m, 2H, Ar‐H), 6.55 – 6.51 (m, 2H, Ar‐H), 1.63 (tt, *J* = 7.9, 4.8 Hz, 1H, cyclopropyl CH), 0.72 (ddt, *J* = 14.8, 4.8, 2.8 Hz, 4H, cyclopropyl CH_2_). ^13^C NMR (101 MHz, DMSO‐*d*_6_) δ 173.17, 149.85, 137.54, 115.07, 79.94, 12.42, 6.85. HRMS (positive ESI) m/z: calcd for C_10_H_12_IN_2_O^+^ [M + H]^+^, 302.9989; found, 302.9976.

#### 4.2.12 N'‐(4‐methoxyphenyl)cyclopropylhydrazide (12)

Reddish brown crystal; yield: 59%; m.p.: 138.1°C–140.0°C; ^1^H NMR (400 MHz, DMSO‐*d*_6_) δ 9.79 (d, *J* = 3.2 Hz, 1H, NH‐CO), 7.49 (d, *J* = 3.1 Hz, 1H, NH‐Ar), 6.95 (d, *J* = 8.2 Hz, 2H, Ar‐H), 6.61 (d, *J* = 8.4 Hz, 2H, Ar‐H), 3.75 (s, 3H, OCH_3_), 1.67 – 1.61 (m, 1H, cyclopropyl CH), 0.75 – 0.68 (m, 4H, cyclopropyl CH_2_). ^13^C NMR (101 MHz, DMSO‐*d*_6_) δ 173.06, 147.70, 129.55, 127.41, 112.77, 20.59, 54.84, 6.68. HRMS (positive ESI) m/z: calcd for C_11_H_15_N_2_O_2_^+^ [M + H]^+^, 207.1128; found, 207.1120.

#### 4.2.13 N'‐(4‐ethylphenyl)cyclopropylhydrazide (13)

Light yellow crystal; yield: 63%; m.p.: 144.1°C–145.3°C; ^1^H NMR (400 MHz, DMSO‐*d*_6_) δ 9.80 (d, *J* = 3.2 Hz, 1H, NH‐CO), 7.51 (d, *J* = 3.1 Hz, 1H, NH‐Ar), 6.98 (d, *J* = 8.5 Hz, 2H, Ar‐H), 6.63 (d, *J* = 8.4 Hz, 2H, Ar‐H), 2.50 – 2.46 (m, 2H, CH_2_CH_3_), 1.64 (tt, *J* = 7.7, 4.8 Hz, 1H, cyclopropyl CH), 1.12 (t, *J* = 7.6 Hz, 3H, CH_2_CH_3_), 0.71 (ddt, *J* = 11.9, 4.9, 2.6 Hz, 4H, cyclopropyl CH_2_). ^13^C NMR (101 MHz, DMSO‐*d*_6_) δ 173.08, 147.94, 134.16, 128.38, 112.75, 27.86, 16.53, 12.44, 6.68. HRMS (positive ESI) m/z: calcd for C_12_H_17_N_2_O^+^ [M + H]^+^, 205.1335; found, 205.1328.

#### 4.2.14 N'‐(4‐trifluoromethylphenyl)cyclopropylhydrazide (14)

Light yellow crystal; yield: 61%; m.p.: 148.6°C–150.5°C; ^1^H NMR (400 MHz, DMSO‐*d*_6_) δ 9.99 (d, *J* = 2.1 Hz, 1H, NH‐CO), 8.35 (d, *J* = 2.2 Hz, 1H, NH‐Ar), 7.47 (d, *J* = 8.6 Hz, 2H, Ar‐H), 6.79 (d, *J* = 8.4 Hz, 2H, Ar‐H), 1.67 (tt, *J* = 7.7, 4.7 Hz, 1H, cyclopropyl CH), 0.75 (ddt, *J* = 10.0, 4.9, 2.8 Hz, 4H, cyclopropyl CH_2_). ^13^C NMR (101 MHz, DMSO‐*d*_6_) δ 173.29, 153.07, 126.63 (q, *J* = 4.04 Hz), 124.16, 118.54 (q, *J* = 32.3 Hz), 111.78, 12.39, 6.92. HRMS (positive ESI) m/z: calcd for C_11_H_12_F_3_N_2_O^+^ [M + H]^+^, 245.0896; found, 245.0886.

#### 4.2.15 N'‐(4‐bromo‐2‐fluorophenyl)cyclopropylhydrazide (15)

Light yellow crystal; yield: 66%; m.p.: 132.4°C–133.8°C; ^1^H NMR (600 MHz, DMSO‐*d*_6_) δ 9.94 (d, *J* = 2.1 Hz, 1H, NH‐CO), 7.85 (s, 1H, NH‐Ar), 7.35 (dd, *J* = 11.2, 2.2 Hz, 1H, Ar‐H), 7.19 (dd, *J* = 8.8, 2.1 Hz, 1H, Ar‐H), 6.66 (t, *J* = 9.0 Hz, 1H, Ar‐H), 1.64 (td, *J* = 8.0, 4.1 Hz, 1H, cyclopropyl CH), 0.77 – 0.69 (m, 4H, cyclopropyl CH_2_). ^13^C NMR (151 MHz, DMSO‐*d*_6_) δ 173.28, 150.19 (d, *J* = 244.6 Hz), 137.19 (d, *J* = 10.6 Hz), 127.89 (d, *J* = 3.02 Hz), 118.46 (d, *J* = 21.1 Hz), 115.23 (d, *J* = 4.53 Hz), 108.37 (d, *J* = 9.06 Hz), 12.42, 6.93. HRMS (positive ESI) m/z: calcd for C_10_H_11_BrFN_2_O^+^ [M + H]^+^, 273.0033(^79^Br), 275.0013(^81^Br); found, 273.0021, 275.0000.

#### 4.2.16 N'‐(4‐nitrophenyl)cyclopropylhydrazide (16)

Reddish brown crystal; yield: 55%; m.p.: 173.0°C–174.5°C; ^1^H NMR (600 MHz, DMSO‐*d*_6_) δ 10.20 (s, 1H, NH‐CO), 9.05 (s, 1H, NH‐Ar), 8.10 – 8.05 (m, 2H, Ar‐H), 6.73 (d, *J* = 9.2 Hz, 2H, Ar‐H), 1.67 (tt, *J* = 8.1, 4.6 Hz, 1H, cyclopropyl CH), 0.82 – 0.73 (m, 4H, cyclopropyl CH_2_). ^13^C NMR (151 MHz, DMSO‐*d*_6_) δ 173.29, 155.46, 138.38, 126.45, 110.90, 12.43, 7.18. HRMS (positive ESI) m/z: calcd for C_10_H_12_N_3_O_3_^+^ [M + H]^+^, 222.0873; found, 222.0864.

#### 4.2.17 N'‐(benzyl)cyclopropylhydrazide (17)

White solid; yield: 59%; m.p.: 138.6°C–140.1°C; ^1^H NMR (600 MHz, DMSO‐*d*_6_) δ 9.47 (d, *J* = 3.5 Hz, 1H, NH‐CO), 7.33 (d, *J* = 4.4 Hz, 4H, Ar‐H), 7.25 (p, *J* = 4.2 Hz, 1H, Ar‐H), 3.87 – 3.82 (m, 2H, CH_2_‐Ph), 1.46 (tt, *J* = 7.9, 4.7 Hz, 1H, cyclopropyl CH), 0.70 – 0.62 (m, 4H, cyclopropyl CH_2_). ^13^C NMR (151 MHz, DMSO‐*d*_6_) δ 172.61, 139.25, 128.89, 128.63, 127.37, 55.17, 12.50, 6.48. HRMS (positive ESI) m/z: calcd for C_11_H_15_N_2_O^+^ [M + H]^+^, 191.1179; found, 191.1171.

#### 4.2.18 N'‐(cyclohexyl)cyclopropylhydrazide (18)

Light yellow crystal; yield: 66%; m.p.: 122.5°C‐124.3°C; ^1^H NMR (600 MHz, DMSO‐*d*_6_) δ 9.52 – 9.34 (m, 1H, NH‐CO), 4.63 (s, 1H, NH‐cyclohexyl), 2.61 – 2.54 (m, 1H, cyclohexyl CH), 1.77 – 1.70 (m, 2H, cyclohexyl CH_2_), 1.66 (dt, *J* = 12.6, 3.7 Hz, 2H, cyclohexyl CH_2_), 1.56 – 1.46 (m, 2H, cyclohexyl CH_2_, cyclopropyl CH), 1.21 – 1.09 (m, 3H, cyclohexyl CH_2_), 1.07 – 0.98 (m, 2H, cyclohexyl CH_2_), 0.69 – 0.62 (m, 4H, cyclopropyl CH_2_). ^13^C NMR (151 MHz, DMSO‐*d*_6_) δ 172.17, 58.48, 31.43, 26.19, 24.39, 12.47, 6.42. HRMS (positive ESI) m/z: calcd for C_10_H_19_N_2_O^+^ [M + H]^+^, 183.1492; found, 183.1485.

### 4.3 General Synthesis of Compounds **19**–**23**

To a solution of the substituted aniline (10 mmol, 1.0 equiv) in dry tetrahydrofuran was added sodium bicarbonate (1.008 g, 12 mmol, 1.2 equiv), followed by the dropwise addition of cyclopropanecarbonyl chloride (1.254 g, 12 mmol, 1.2 equiv) at 0°C. After completion of the reaction, the mixture was diluted with ethyl acetate (3 × 30 mL), washed with saturated saline and dried over anhydrous sodium sulfate, and the solvent was removed under reduced pressure. The residue was purified by column chromatography (petroleum ether/ethyl acetate) to afford the target compounds **19**‐**23** [35].

#### 4.3.1 N‐phenylcyclopropanecarboxamide (19)

White crystal; yield: 92%; m.p.: 112.5°C–113.7°C. The ^1^H NMR, ^1^ ^3^C NMR, and HRMS data were basically consistent with the data of literature [36].

#### 4.3.2 N‐(4‐fluorophenyl)cyclopropanecarboxamide (20)

White crystal; yield: 87%; m.p.: 158.8°C–160.2°C. The ^1^H NMR, ^1^ ^3^C NMR, and HRMS data were basically consistent with the data of literature [37].

#### 4.3.3 N‐(4‐chlorophenyl)cyclopropanecarboxamide (21)

White crystal; yield: 86%; m.p.: 165.2°C–166.1°C. The ^1^H NMR, ^1^ ^3^C NMR, and HRMS data were basically consistent with the data of literature [38].

#### 4.3.4 N‐(4‐bromophenyl)cyclopropanecarboxamide (22)

White crystal; yield: 90%; m.p.: 188.6°C–190.01°C. The ^1^H NMR, ^1^ ^3^C NMR, and HRMS data were basically consistent with the data of literature [39].

#### 4.3.5 N‐(4‐iodophenyl)cyclopropanecarboxamide (23)

White crystal; yield: 84%; m.p.: 198.1°C–199.7°C. The ^1^H NMR, ^1^ ^3^C NMR, and HRMS data were basically consistent with the data of literature [40].

### 4.4 Antifungal Activity Assay In Vitro

The in vitro antifungal activity of compounds **1**‐**23** was evaluated by the mycelial linear growth rate method at 50 µg/mL. Each test compound (10 mg) was dissolved in 5% DMSO (10 mL), and the solution was thoroughly mixed with sterilized potato dextrose agar (PDA) medium (190 mL). Fungal plugs (d = 5 mm) were then placed on the amended PDA medium and incubated for 72 h. Inhibition rates were calculated based on colony diameter measurements, with each assay performed in triplicate. Where complete inhibition (100%) was reached by all three replicates at a given concentration, the standard deviation was reported as 0.0. Thiabendazole and kresoxim‐methyl, two commercial fungicides, were used as positive controls.

The median effective concentration (EC_50_) values of active compounds were determined using the same method. Inhibition rates were measured across a concentration series of 50, 40, 30, 20, 10, 5, 2.5, and 1.25 µg/mL. Dose‐response curves were analyzed using GraphPad Prism 7.00 (GraphPad Software Inc., San Diego, CA) to calculate EC_50_ values and 95% confidence interval (95%CI) [41].

### 4.5 SEM Analysis

SEM was conducted according to the methods reported in the literature [42]. The mycelia treated with the parent compound **1**, selected as the template compound because it is representative of the series and its EC_50_ against *A. alternata* (2.73 µg/mL) is in the same order of magnitude as the most potent analogues **4**, **6**, and **7**, at a concentration of 3 µg/mL for 5 days were excised from the colony's edge. The samples were fixed with 4% glutaraldehyde at 4°C for 24 h, then rinsed with 0.1 M buffer (pH = 7.0) and treated with graded ethanol (10, 30, 50, 70, 80, and 90 %, v/v). Then 100% ethanol was used to dehydrate the samples for three times. The dehydrated samples were observed on a Nova Nano SEM‐450 scanning electron microscope after dried at a critical point and coated with gold.

### 4.6 Molecular Docking Analysis

As compound **1** is the parent hydrazide (template compound) of the series, it was selected as a representative compound to explore the mechanism of action of this chemical series through molecular docking. The structure of parent compound **1** drawn in ChemBio Draw Ultra14.0, was imported into ChemBio 3D Ultra14.0 for energy minimization treatment, including the allocation of partial charges and optimization of the force field to generate a stable, low‐energy conformation. The crystal structure of the receptor protein, succinate dehydrogenase (SDH, avian mitochondrial respiratory complex II, Gallus gallus, PDB code: 2FBW), was retrieved from the Research Collaboratory for Structural Bioinformatics Protein Data Bank (RCSB PDB). In order to streamline the docking environment, the crystal structure of 2FBW was pretreated by removing water molecules and irrelevant substructures [9]. the FAD cofactor was retained, and the receptor and ligand were converted to PDBQT format using AutoDockTools (MGLTools 1.5.7) with polar hydrogens, Gasteiger partial charges, and all rotatable bonds assigned, with the receptor kept rigid. Docking was performed using AutoDock Vina 1.2.5 with the built‐in Vina scoring function; the grid box (40.0 Å × 40.0 Å × 40.0 Å) was centered on the N5 atom of the FAD cofactor (x = 37.411, y = −49.677, z = 29.417 Å), with an exhaustiveness of 60 and up to 100 output poses, and the top‐ranked pose was used for subsequent analysis.

### 4.7 Statistical Analysis

All experiments were performed in triplicate (n = 3), and all results are expressed as the mean ± standard deviation (SD). Inhibition rates were calculated directly from the raw colony diameter measurements, and no data transformation, normalization, or outlier exclusion was applied. The EC_50_ values and 95% CI were calculated by nonlinear regression analysis using GraphPad Prism 7.00 (GraphPad Software Inc., San Diego, CA, USA). No additional statistical tests were applied.

During the preparation of this manuscript, WorkBuddy (Tencent) was used solely for English language editing, grammatical correction, and sentence structure refinement. All research conception, experimental design, data analysis, result interpretation, and manuscript drafting were completed independently by the authors. The authors take full responsibility for all content of this article.

## Author Contributions

**Shanshan Yang**: writing – review and editing, writing – original draft, investigation, formal analysis, data curation, conceptualization. **Xinyuan Wang**: writing – original draft, methodology, investigation. **Bixi Yin**: data curation. **Weiting Yan**: visualization, methodology, formal analysis. **Lei Wang**: software, methodology, investigation. **Yunying Sha**: methodology, formal analysis, conceptualization. **Lizhong Wang**: methodology. **Zhuyun Liu**: data curation, conceptualization, writing – review and editing.

## Conflicts of Interest

The authors declare no conflicts of interest.

## Supporting information




**Supporting File 1**: cbdv71820‐sup‐0001‐SuppMat.pdf.

## Data Availability

The data that support the findings of this study are available on request from the corresponding author. The data are not publicly available due to privacy or ethical restrictions.
