# Supplementary material for: Design, Synthesis, and Antifungal Structure‐Activity Relationship of Hydrazide Derivatives Containing a Cyclopropyl Group Against Phytopathogenic Fungi
Source: Chem Biodivers. 2026 Oct 26;23(10):e71820. doi: 10.1002/cbdv.71820 (PMC13698643; doi:10.1002/cbdv.71820)
Supplement: Supplementary file 1 [file CBDV-23-e71820-s001.pdf]

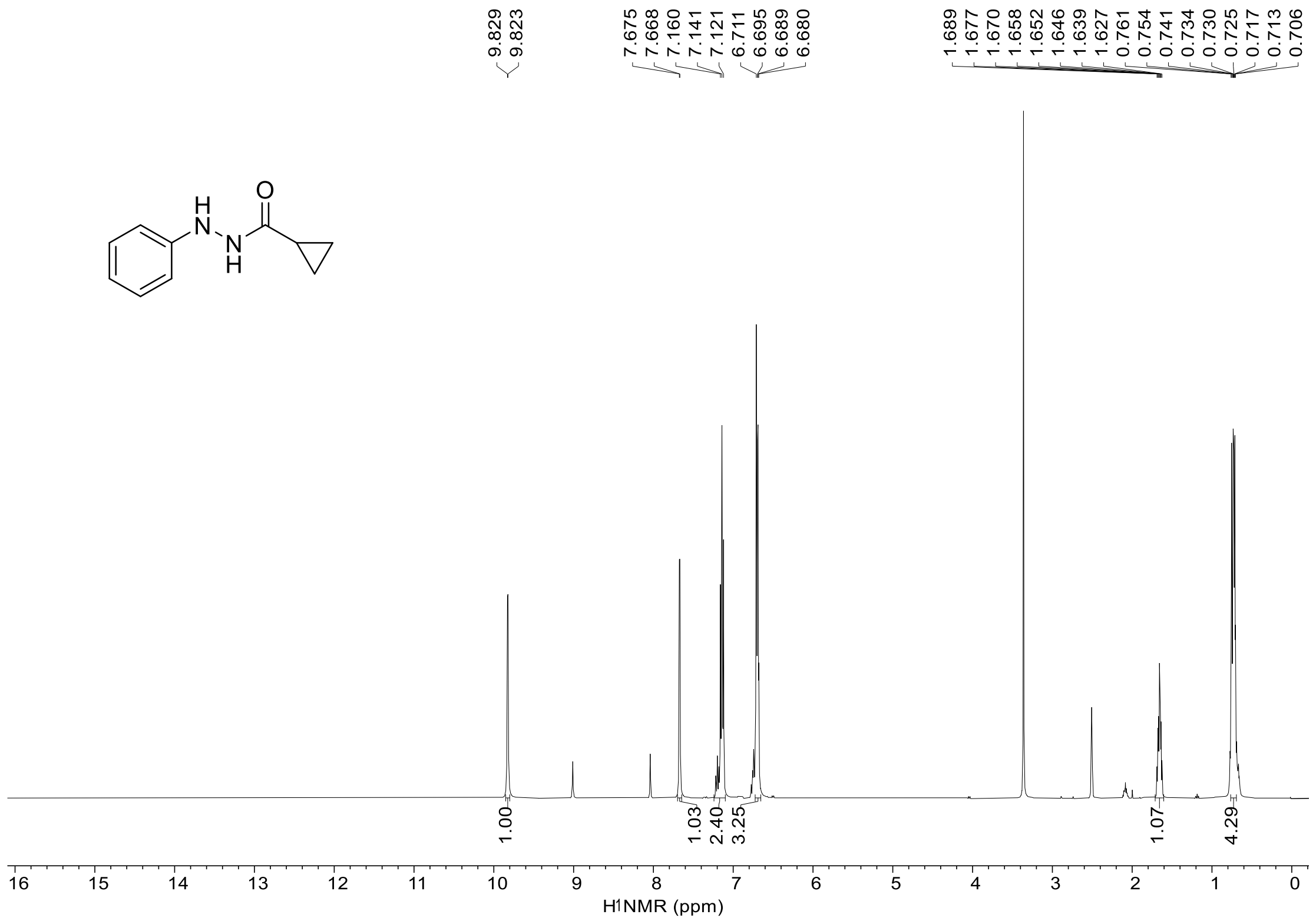

Figure S1 ^{1}H NMR spectrum of compound **1** (400 MHz, DMSO-d_6)

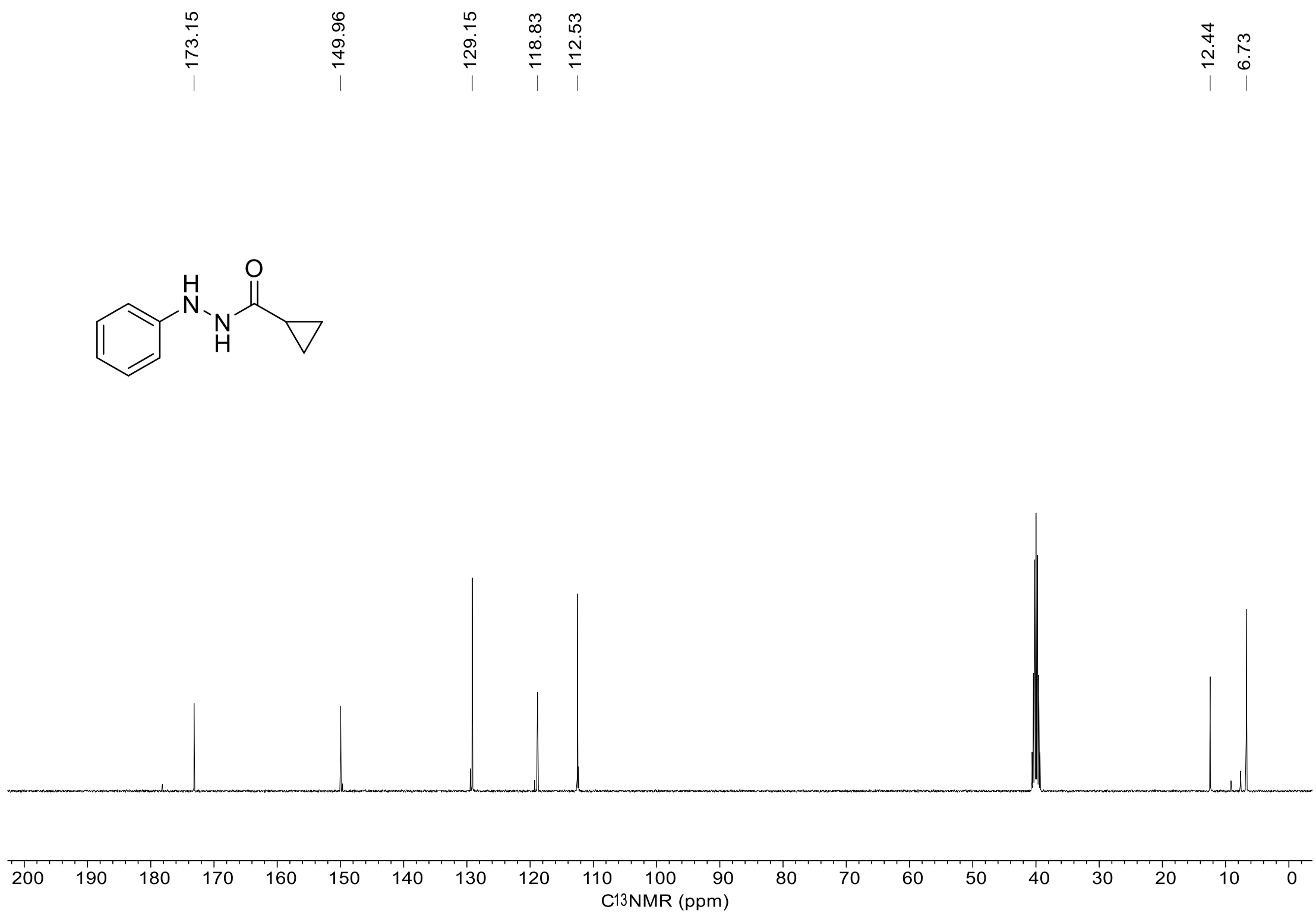

Figure S2 ^{13}C NMR spectrum of compound **1** (101 MHz, DMSO-d_6)

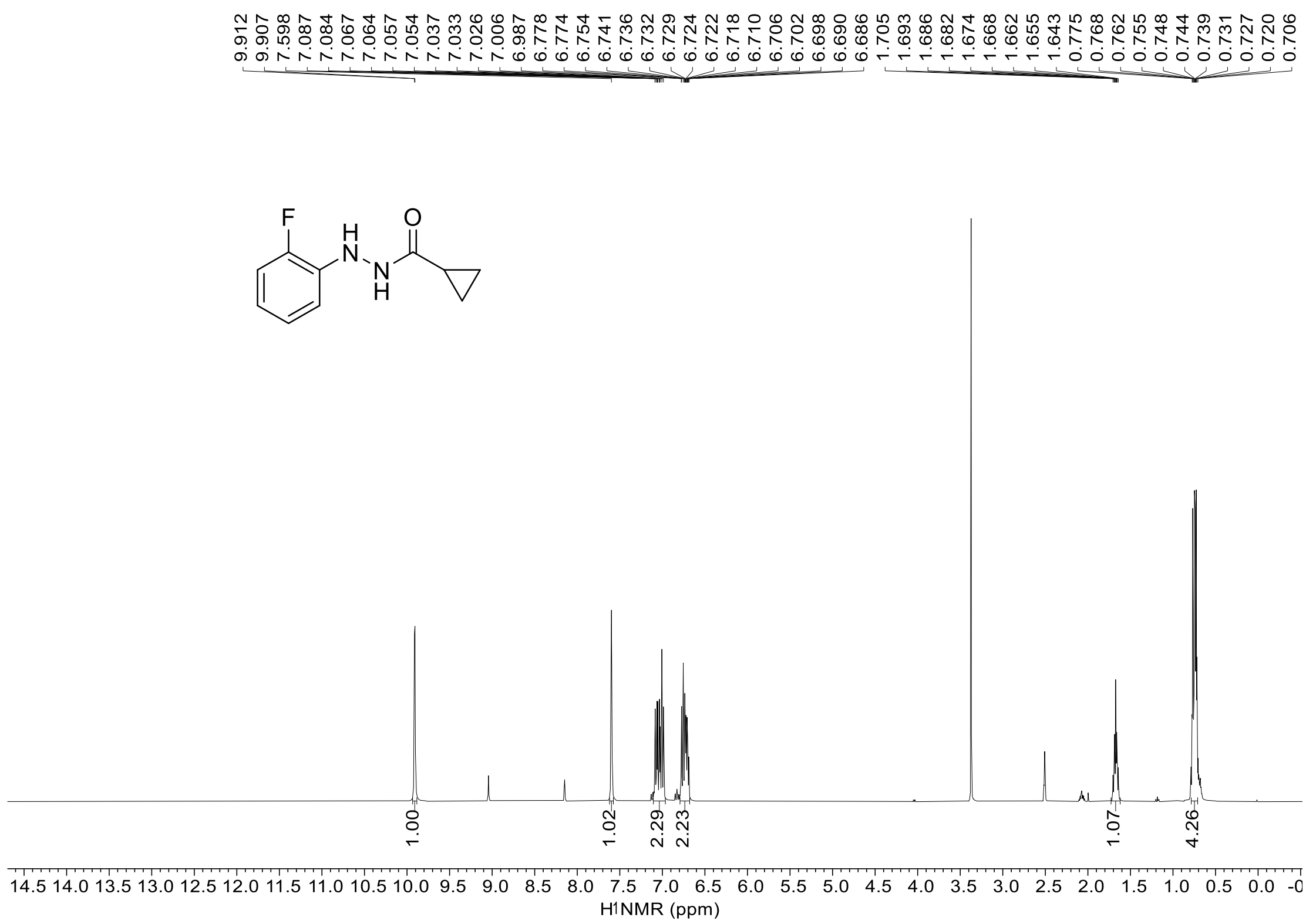

Figure S3 ^{1}H NMR spectrum of compound **2** (400 MHz, DMSO-d_6)

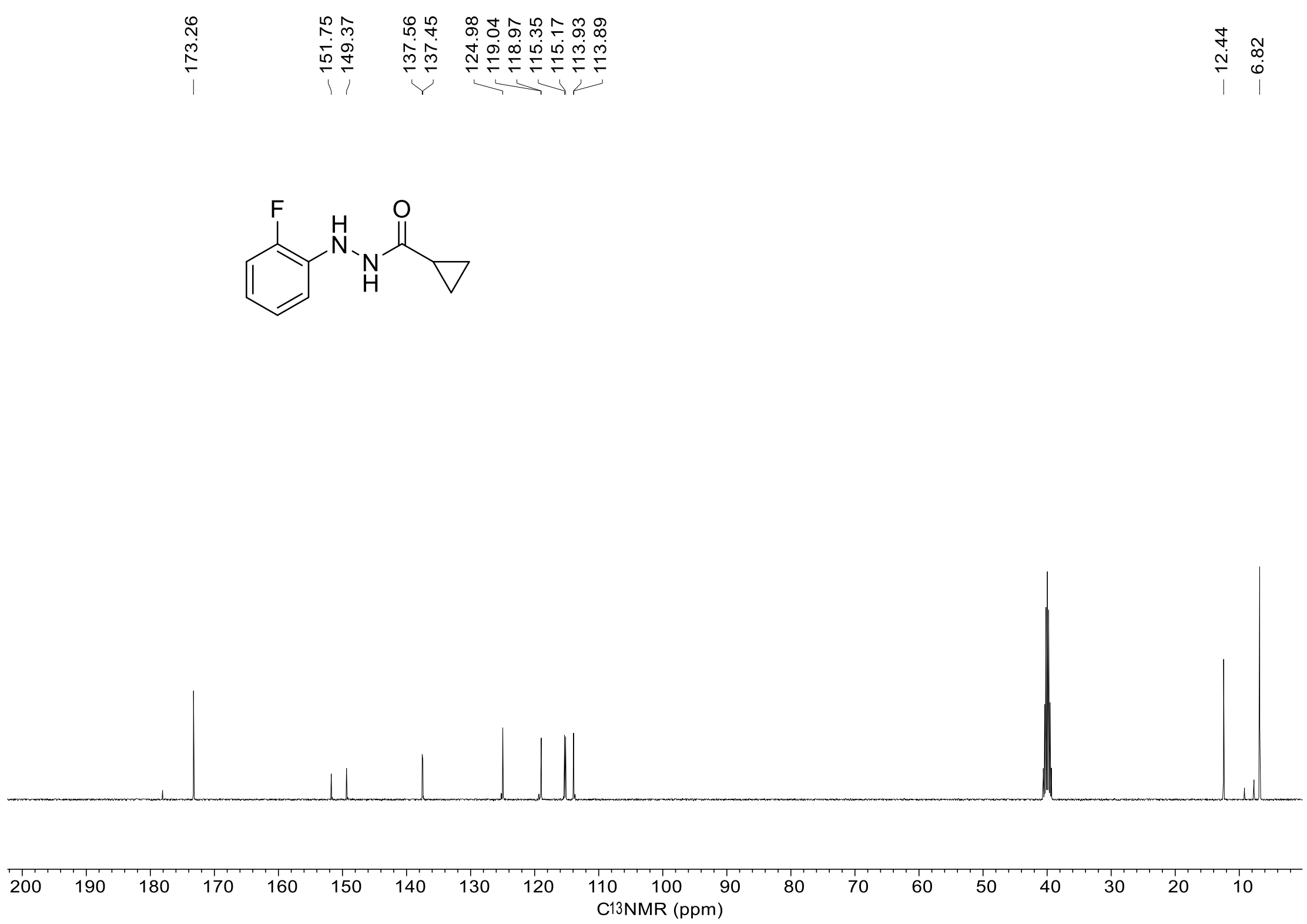

Figure S4 ^{13}C NMR spectrum of compound **2** (101 MHz, DMSO-d_6)

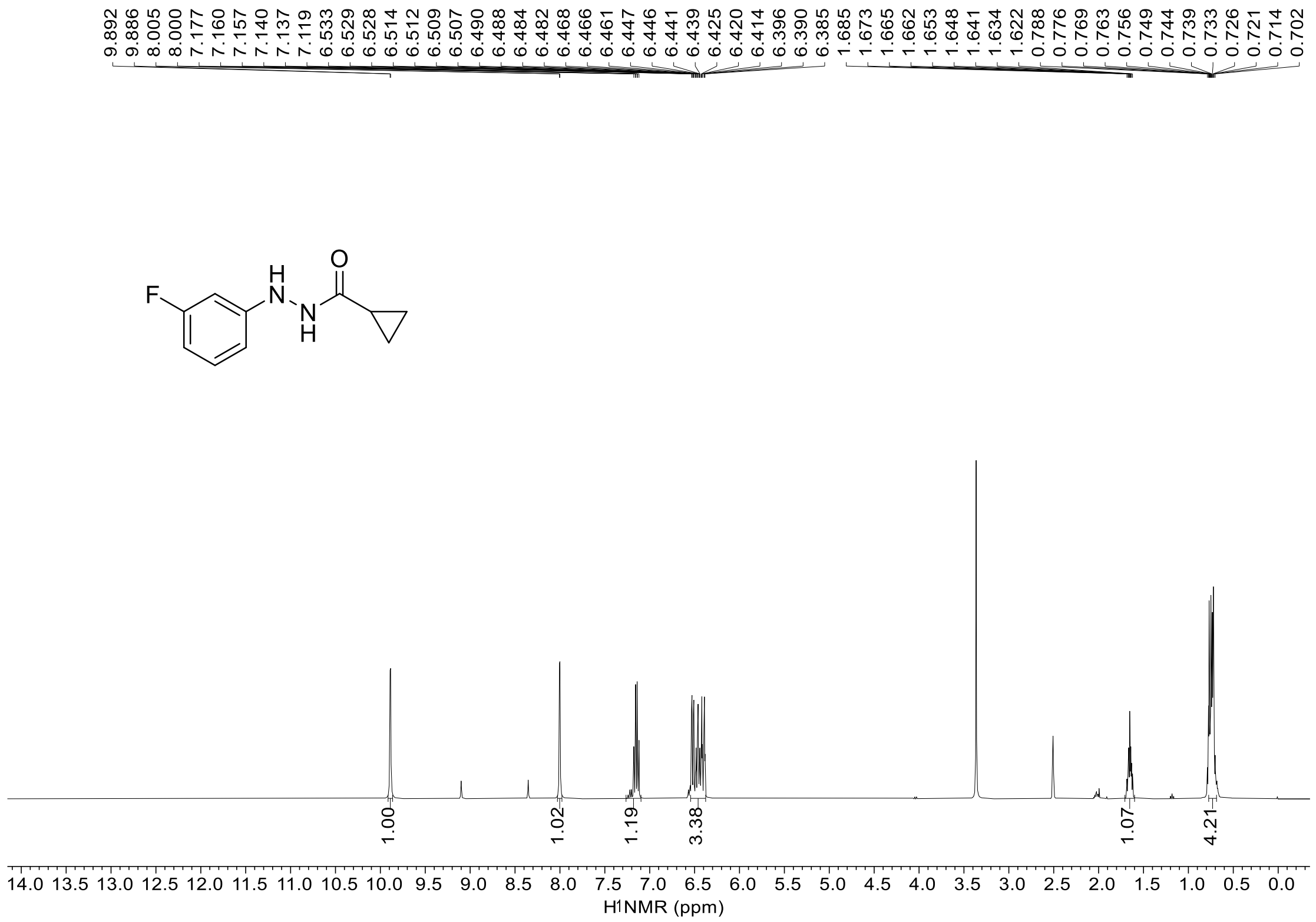

Figure S5 ^{1}H NMR spectrum of compound **3** (400 MHz, DMSO-d_6)

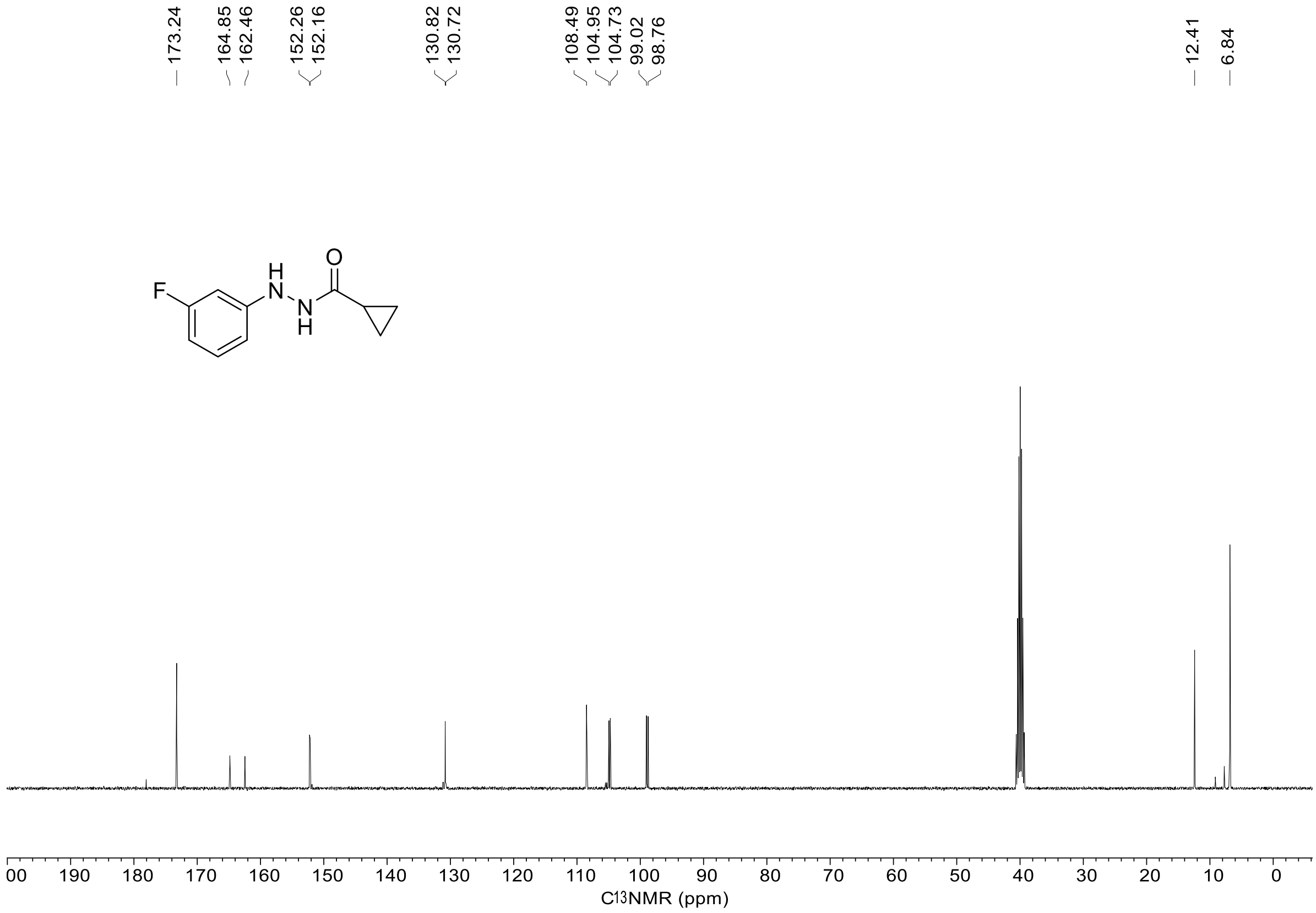

Figure S6 ^{13}C NMR spectrum of compound **3** (101 MHz, DMSO-d_6)

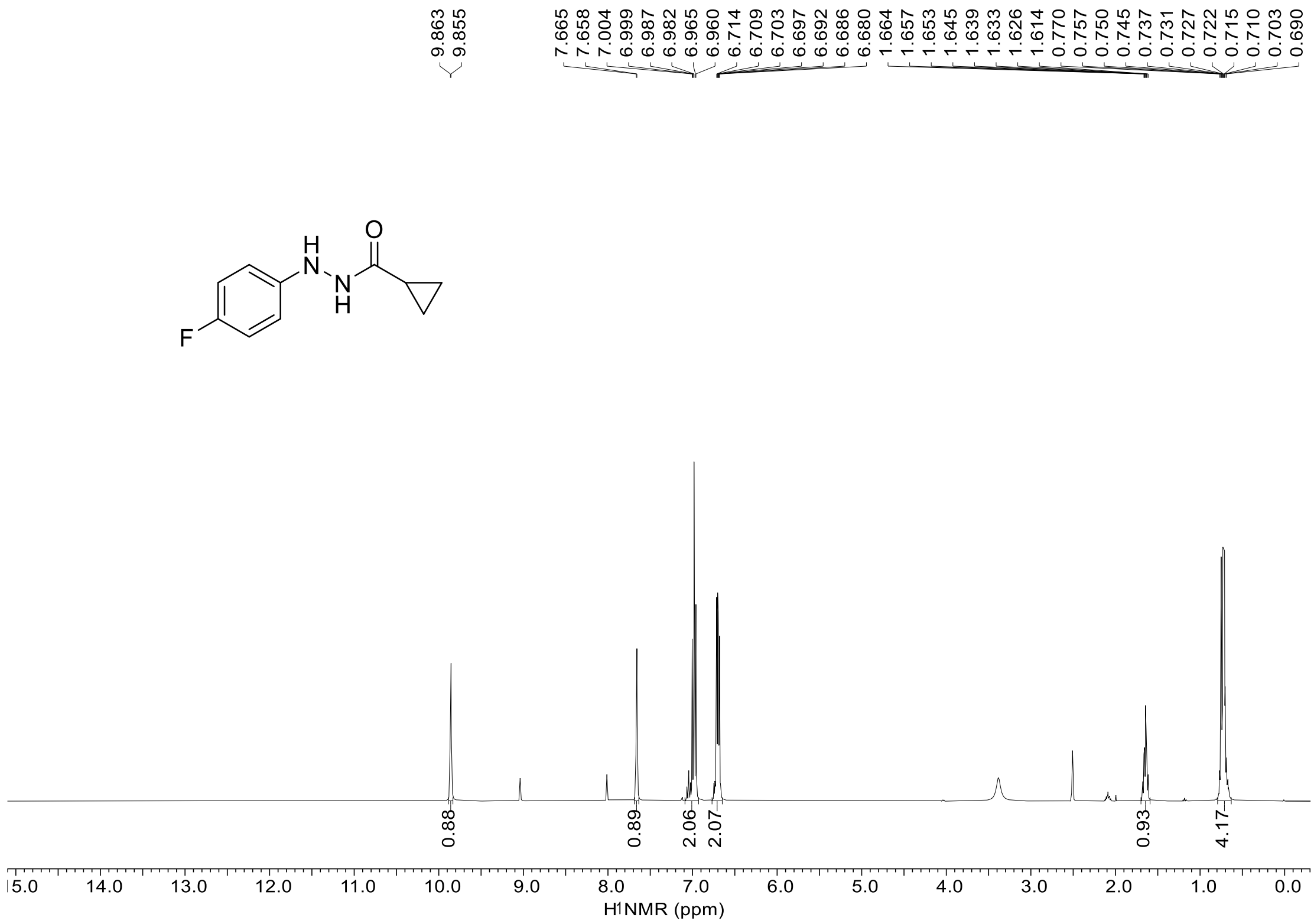

Figure S7 ^{1}H NMR spectrum of compound **4** (400 MHz, DMSO-d_6)

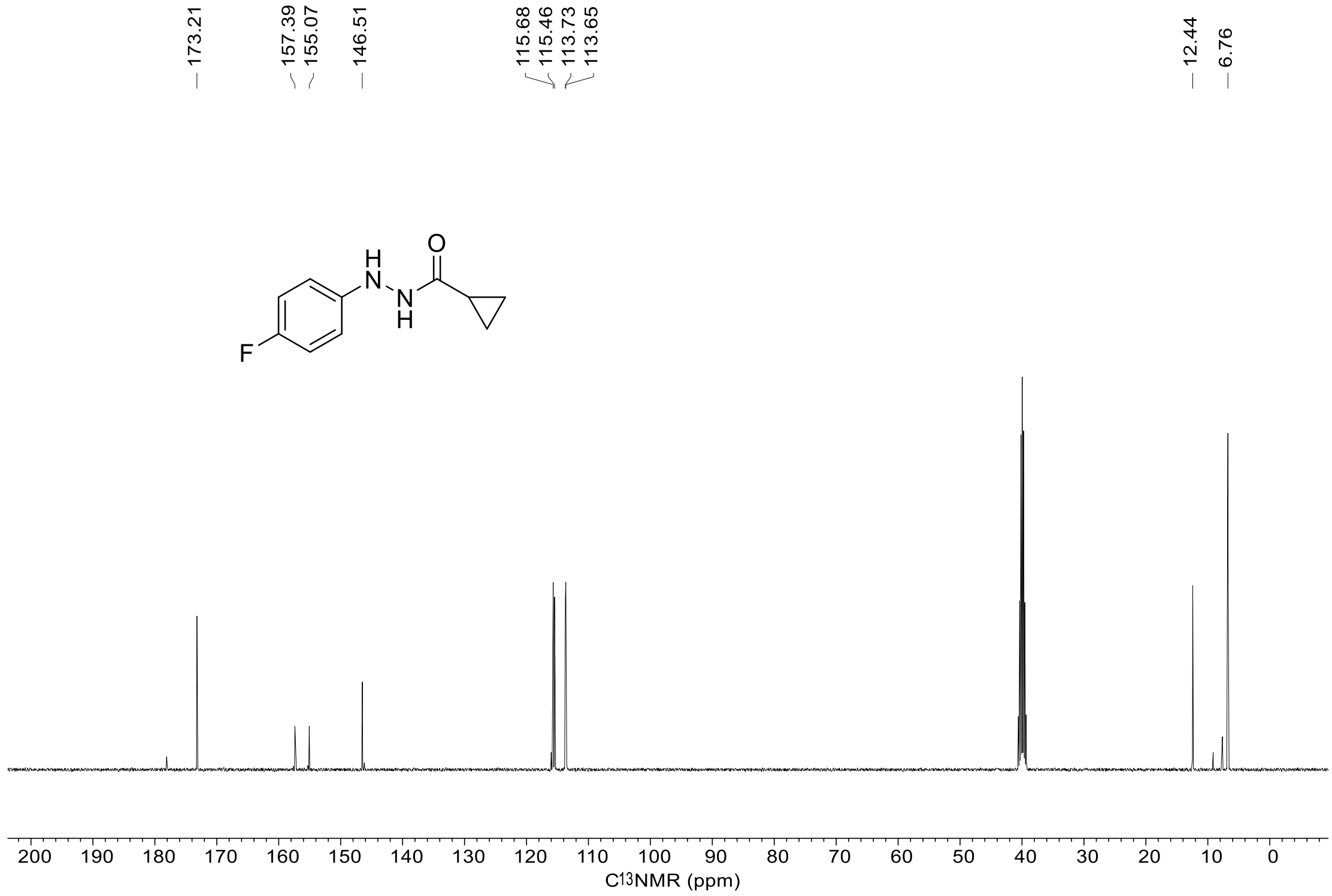

Figure S8 ^{13}C NMR spectrum of compound **4** (101 MHz, DMSO-d_6)

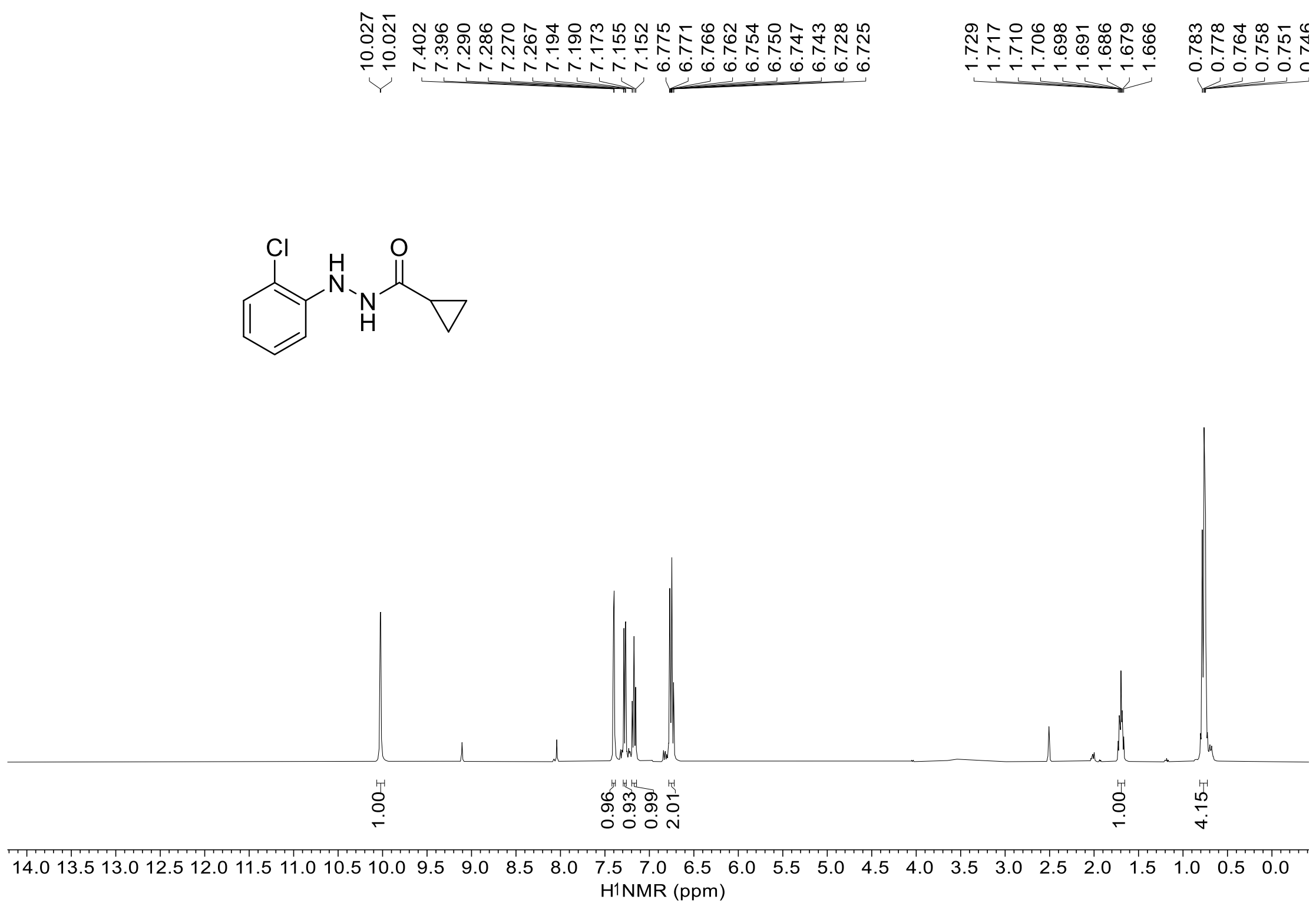

Figure S9 ^{1}H NMR spectrum of compound **5** (400 MHz, DMSO-d_6)

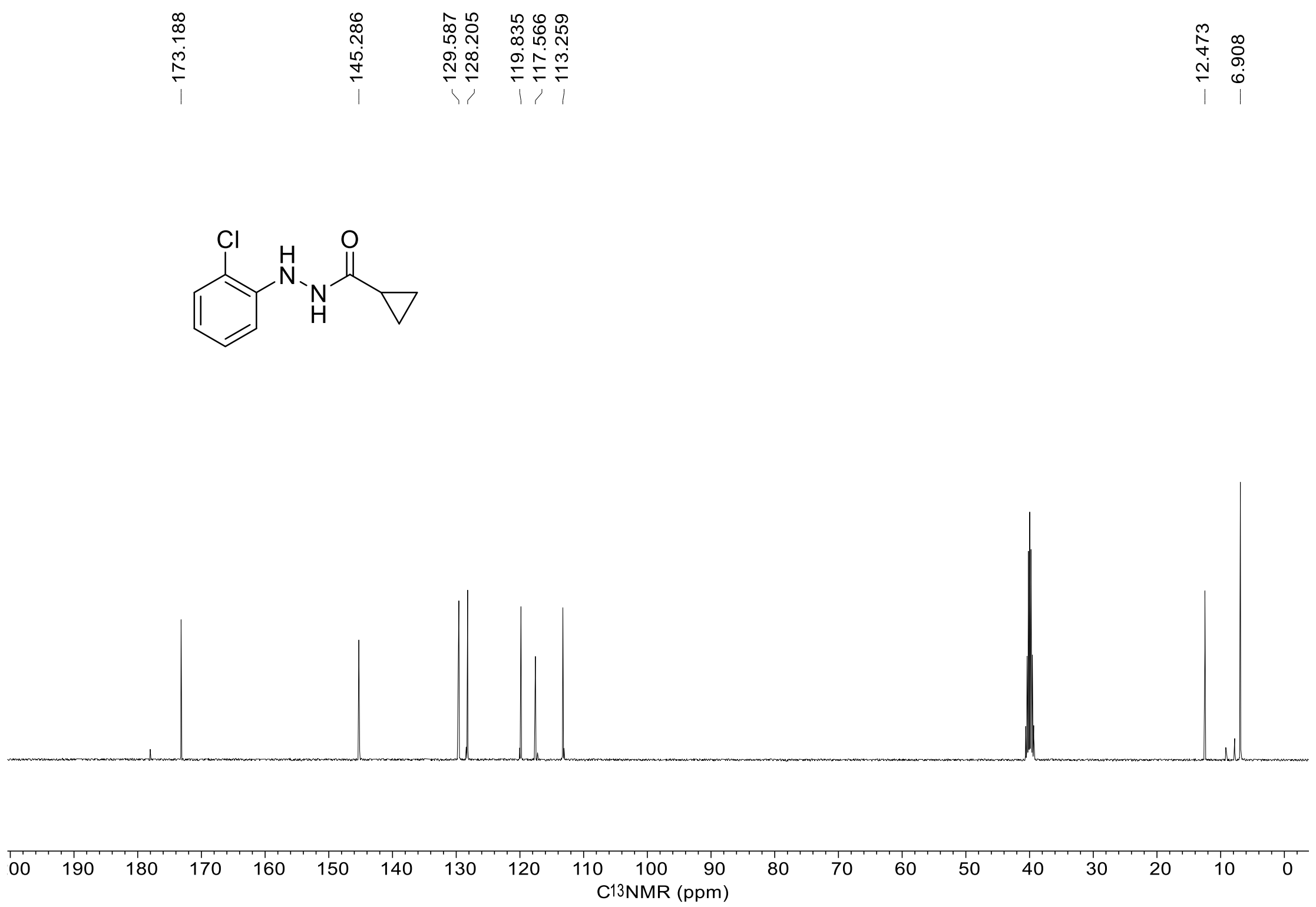

Figure S10 ^{13}C NMR spectrum of compound **5** (101 MHz, DMSO-d_6)

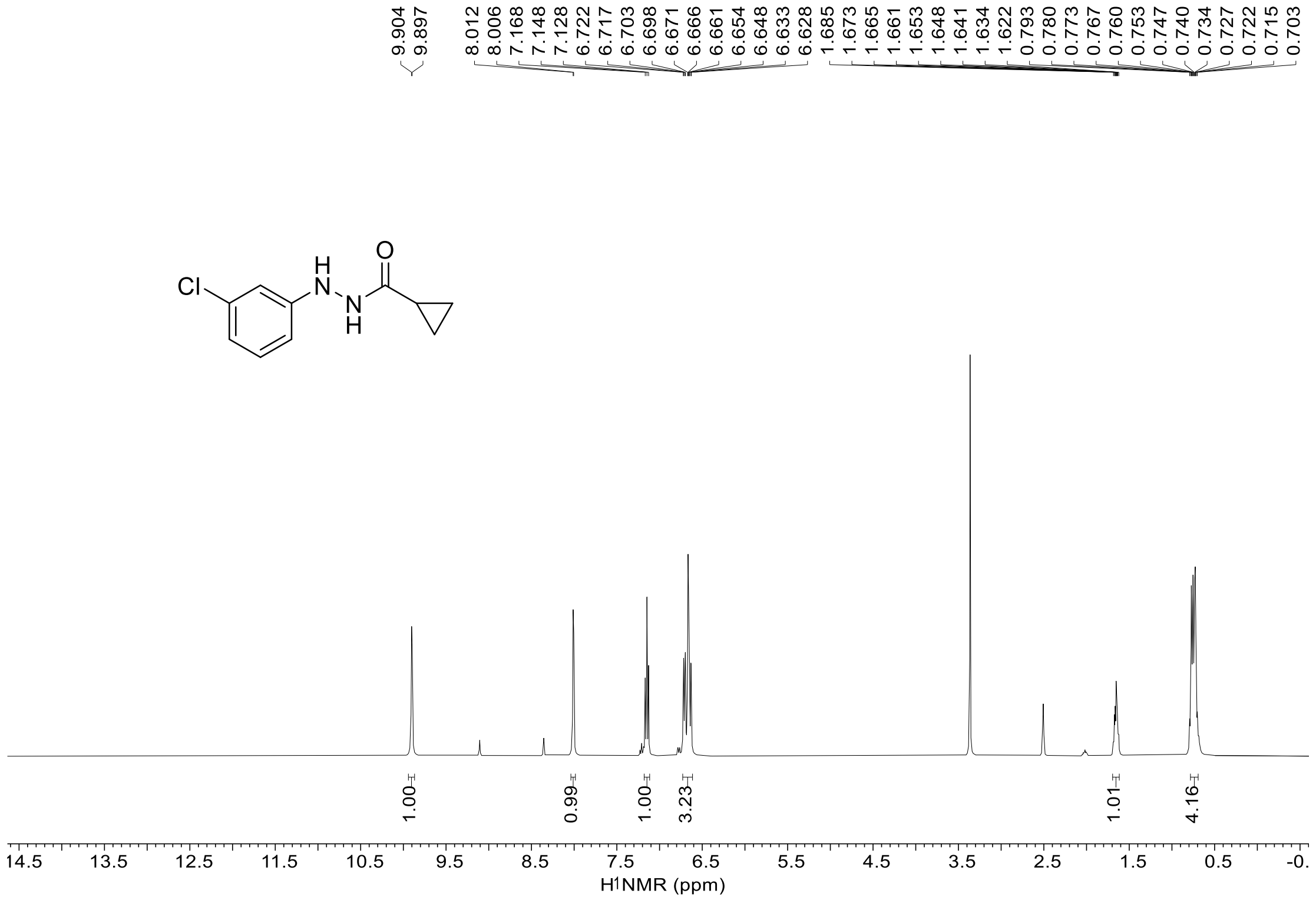

Figure S11 ^{1}H NMR spectrum of compound **6** (400 MHz, DMSO-d_6)

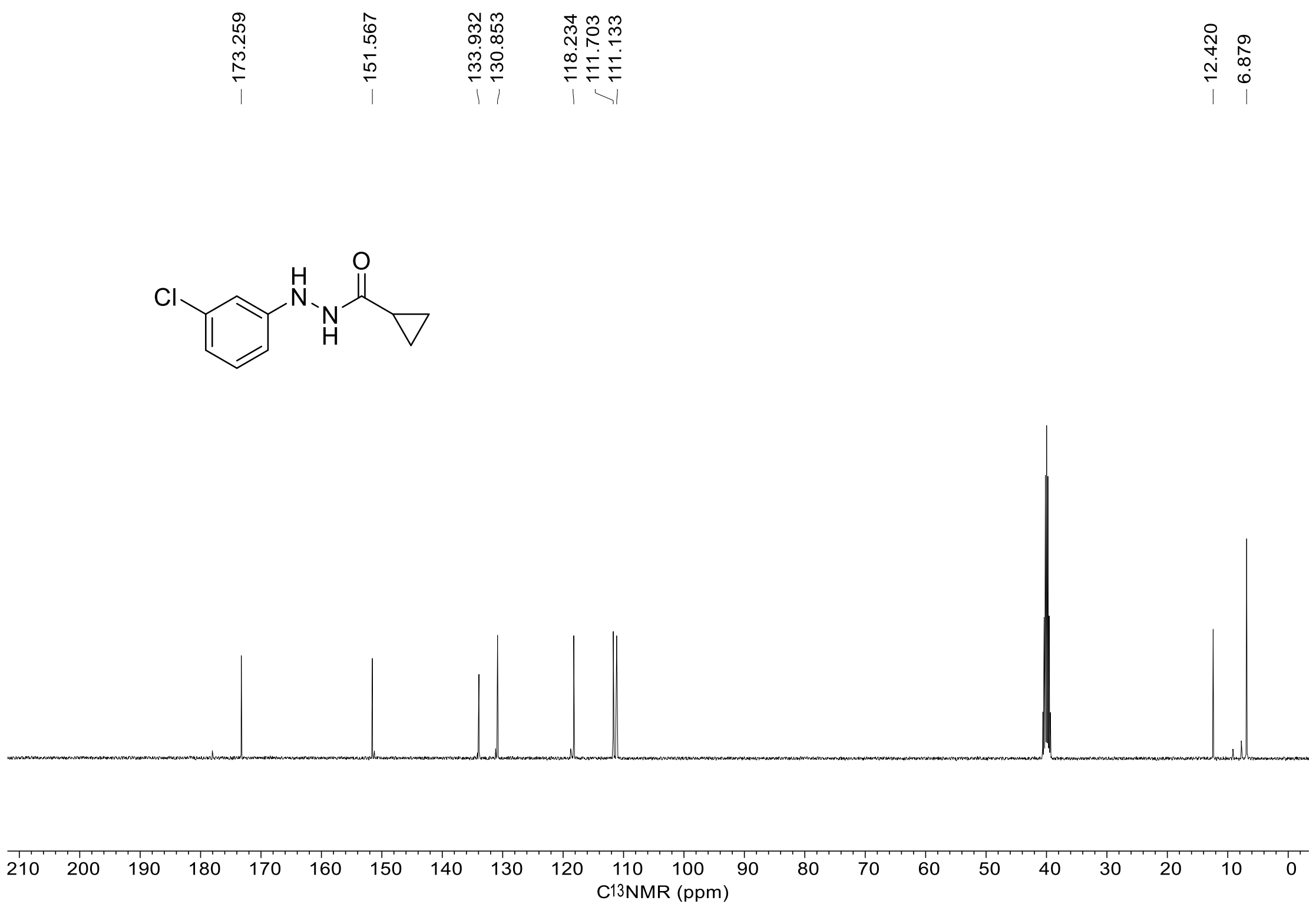

Figure S12 ^{13}C NMR spectrum of compound **6** (101 MHz, DMSO-d_6)

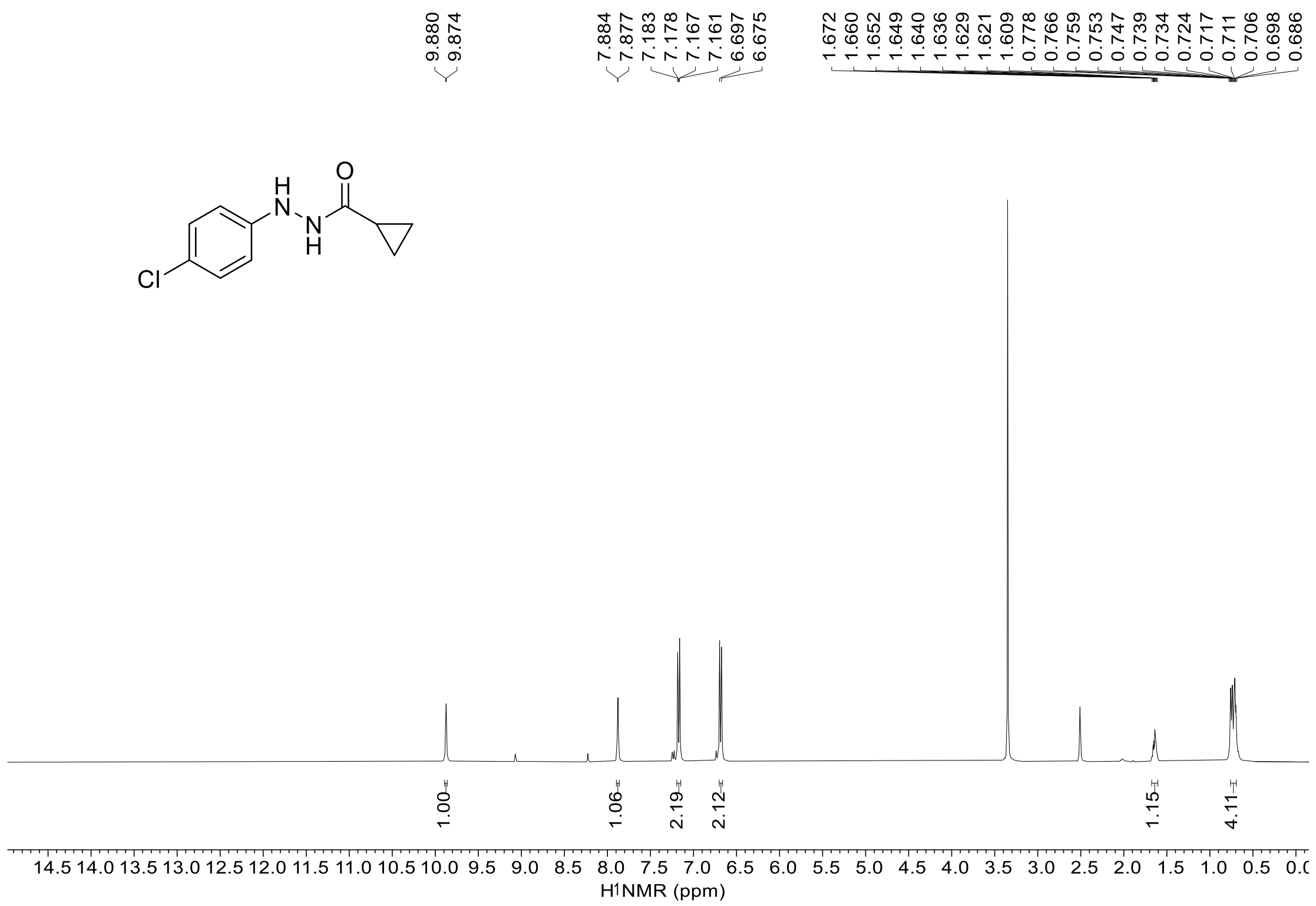

Figure S13 ^{1}H NMR spectrum of compound **7** (400 MHz, DMSO-d_6)

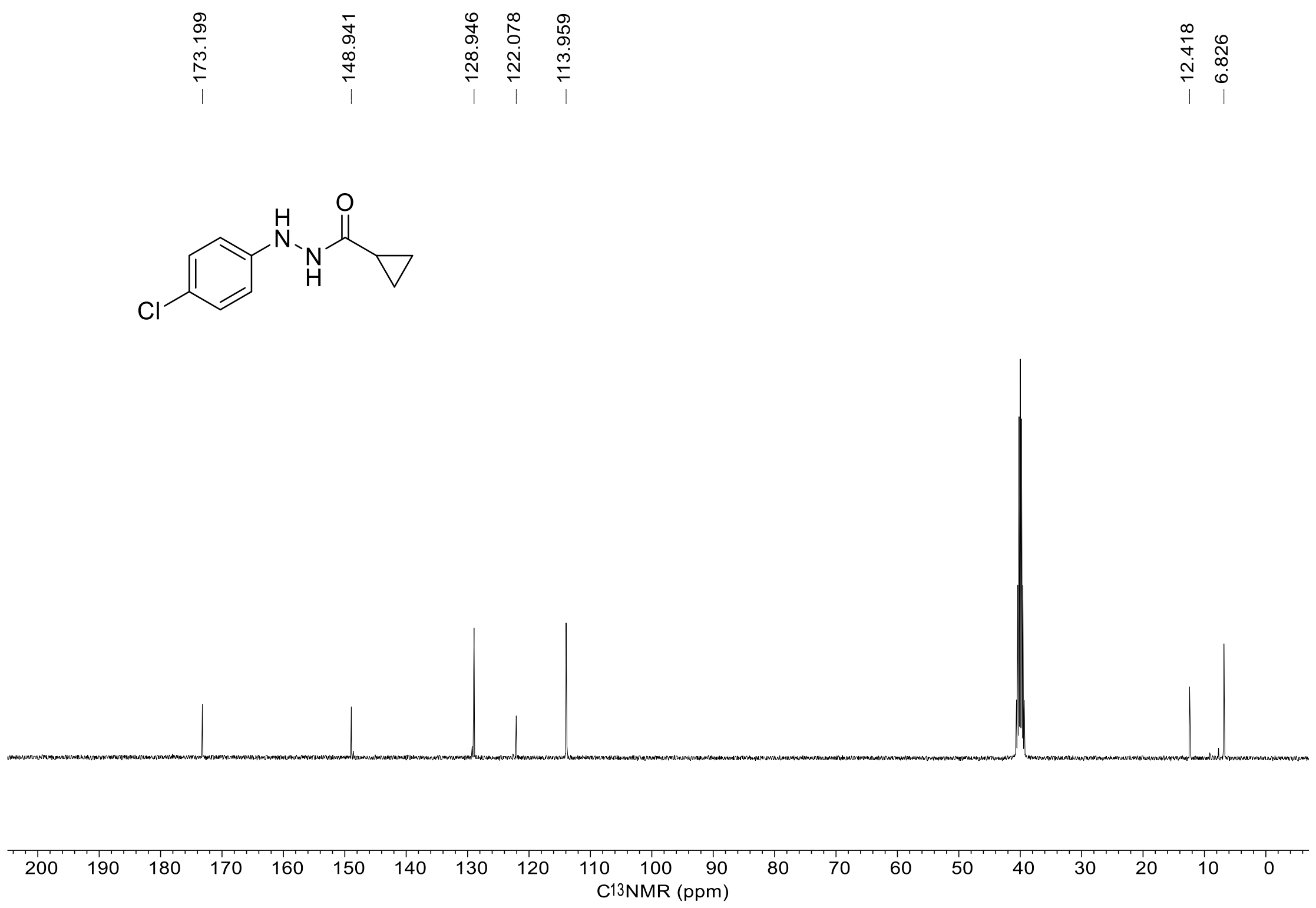

Figure S14 ^{13}C NMR spectrum of compound **7** (101 MHz, DMSO-d_6)

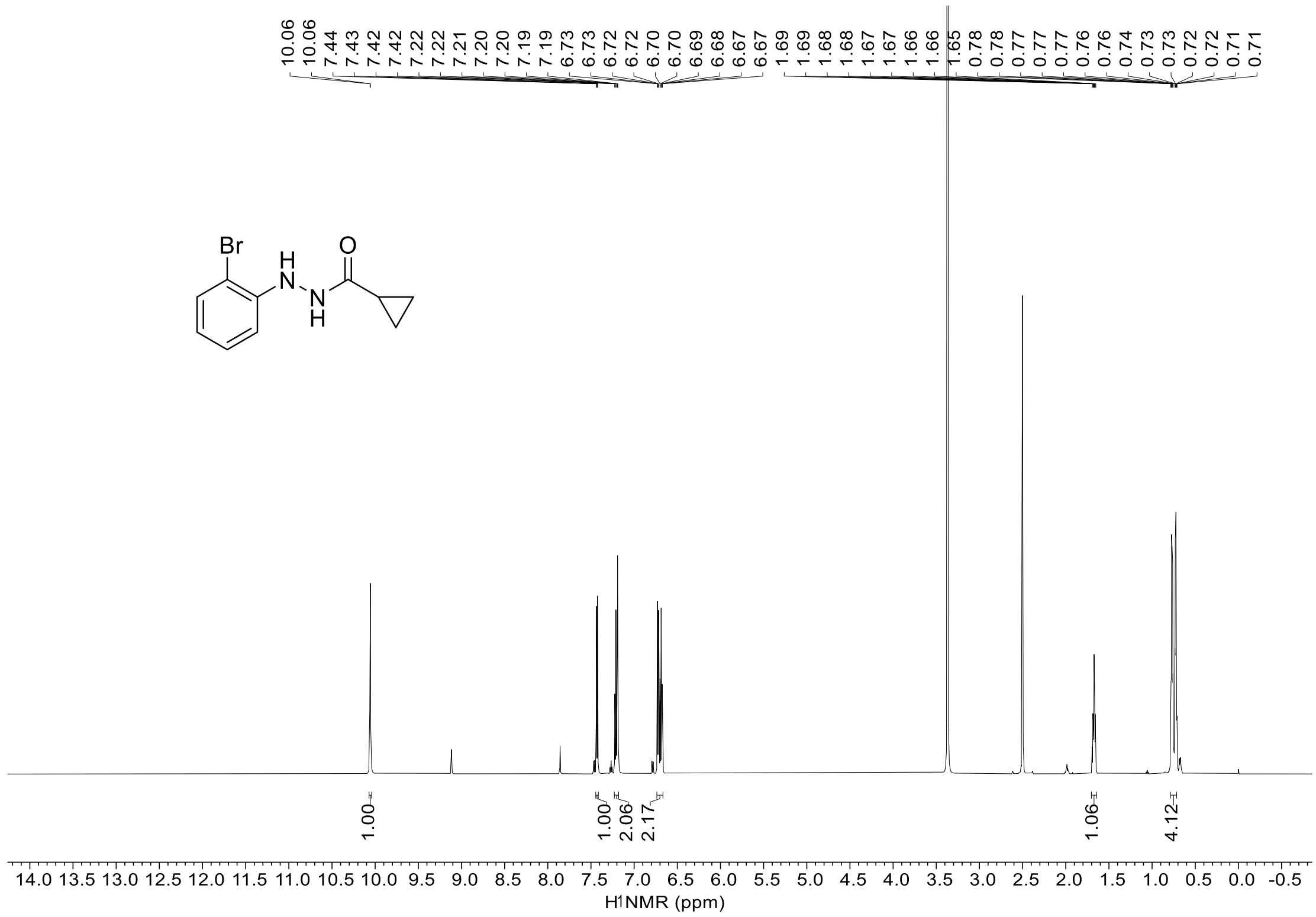

Figure S15 ^{1}H NMR spectrum of compound **8** (600 MHz, DMSO-d_6)

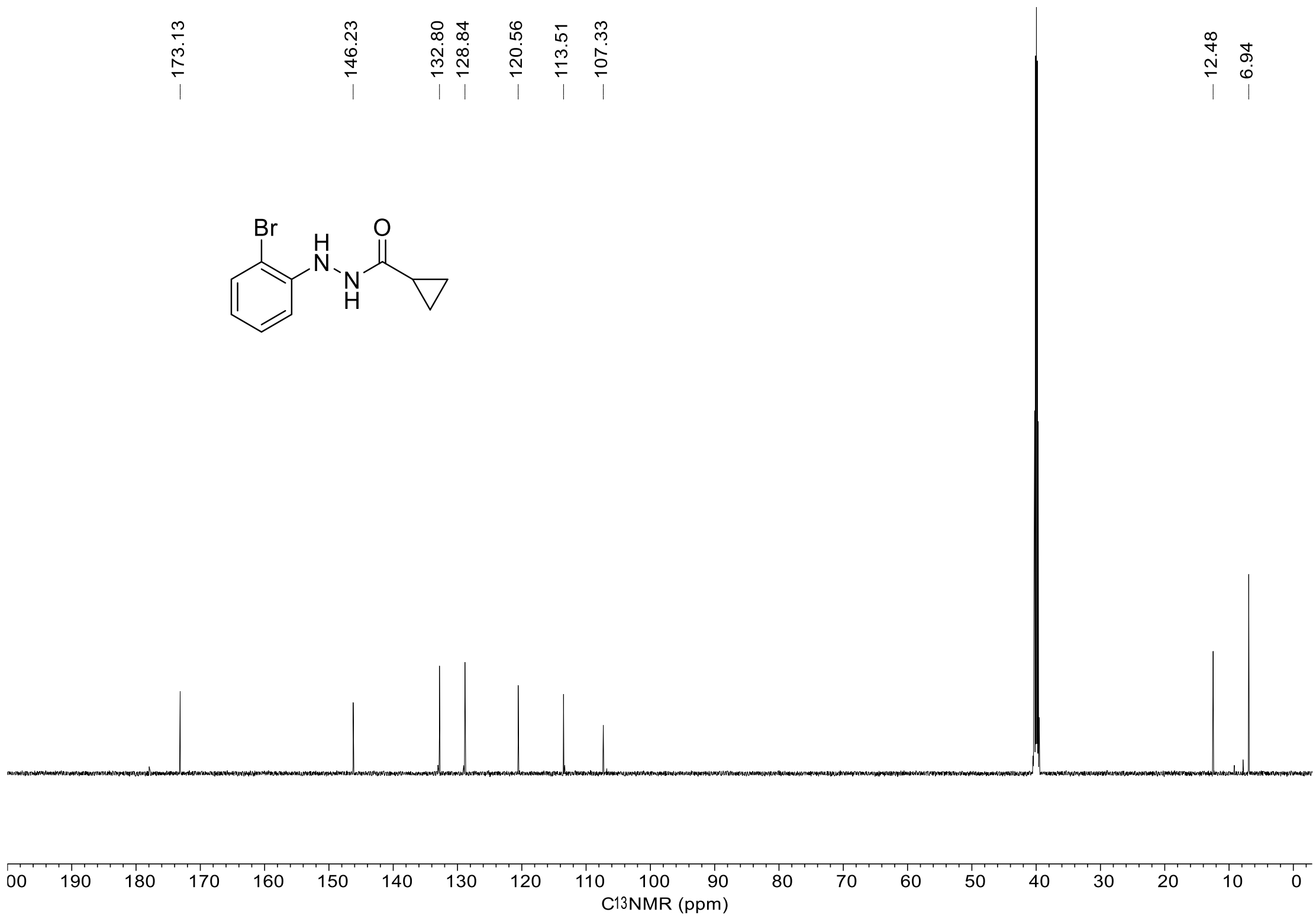

Figure S16 ^{13}C NMR spectrum of compound **8** (151 MHz, DMSO-d_6)

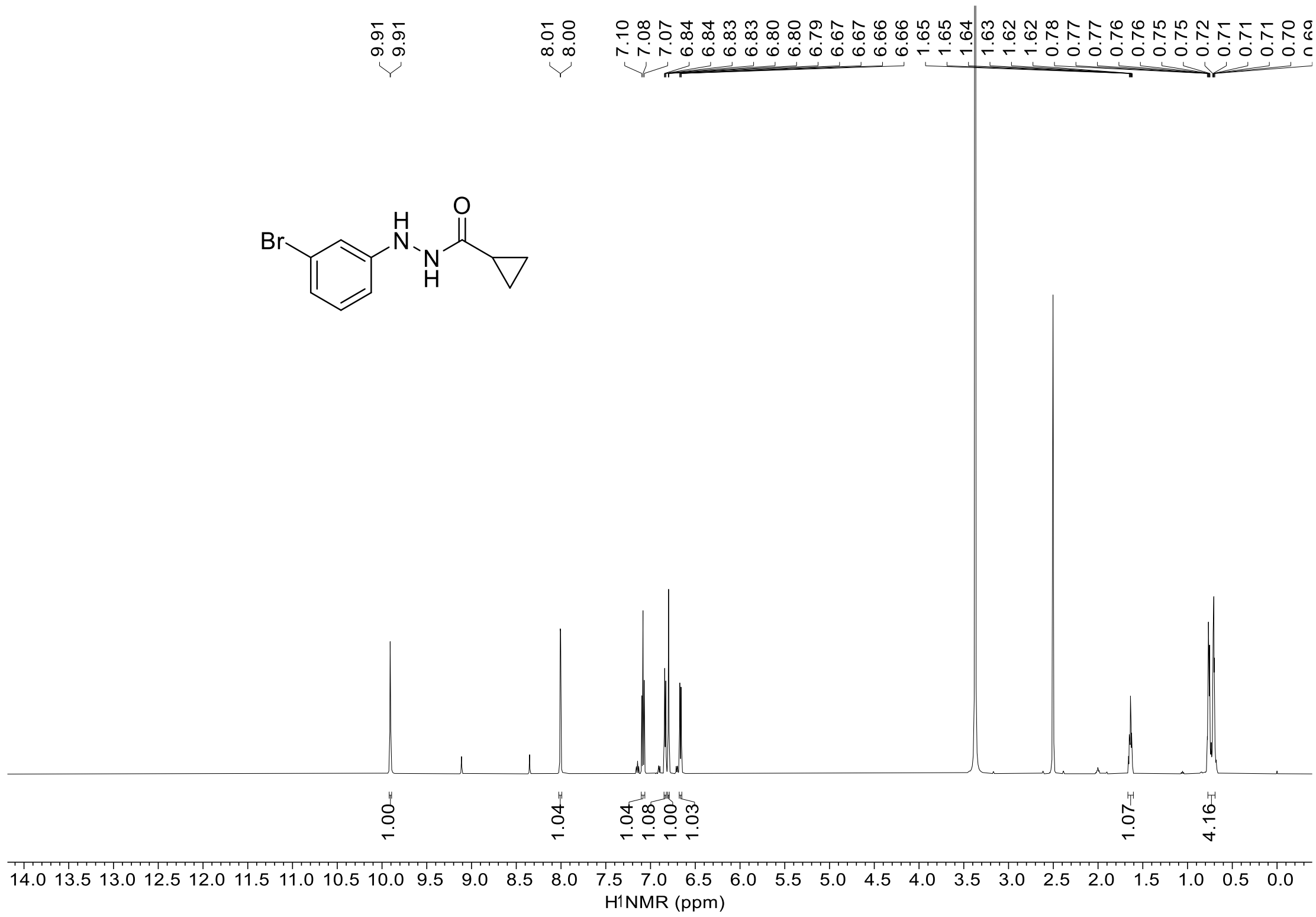

Figure S17 ^{1}H NMR spectrum of compound **9** (600 MHz, DMSO-d_6)

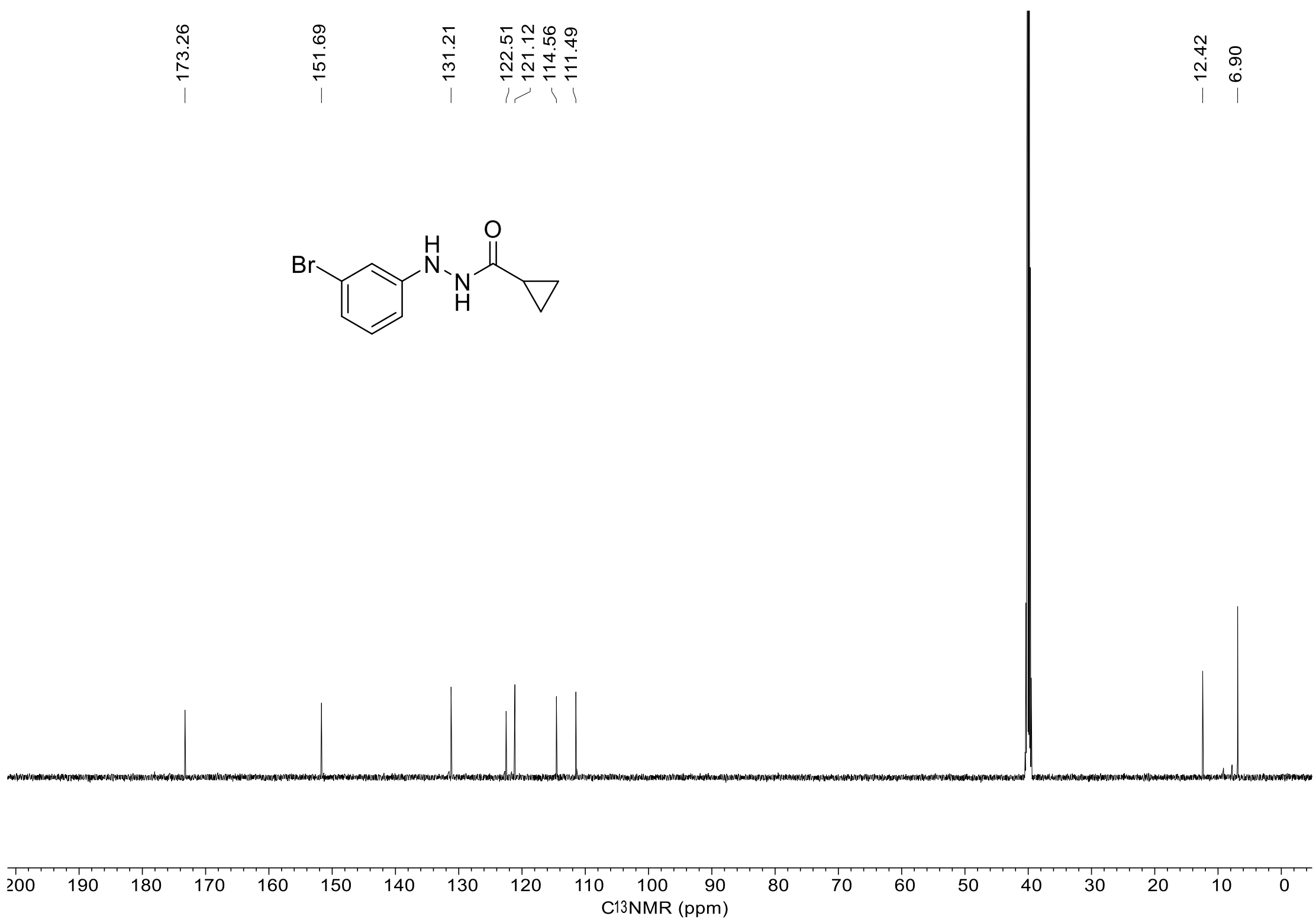

Figure S18 ^{13}C NMR spectrum of compound **9** (151 MHz, DMSO-d_6)

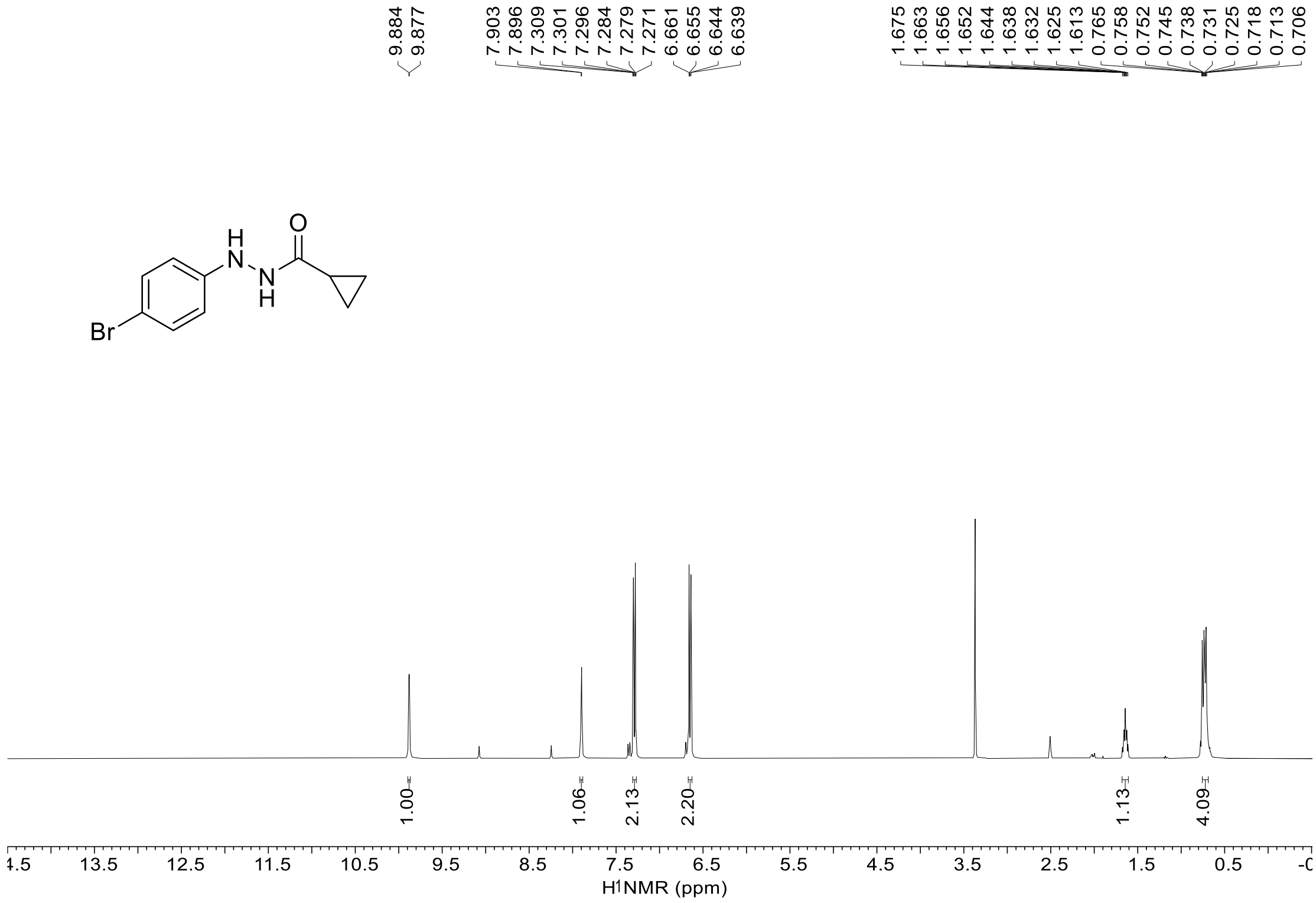

Figure S19 ^{1}H NMR spectrum of compound **10** (400 MHz, DMSO-d_6)

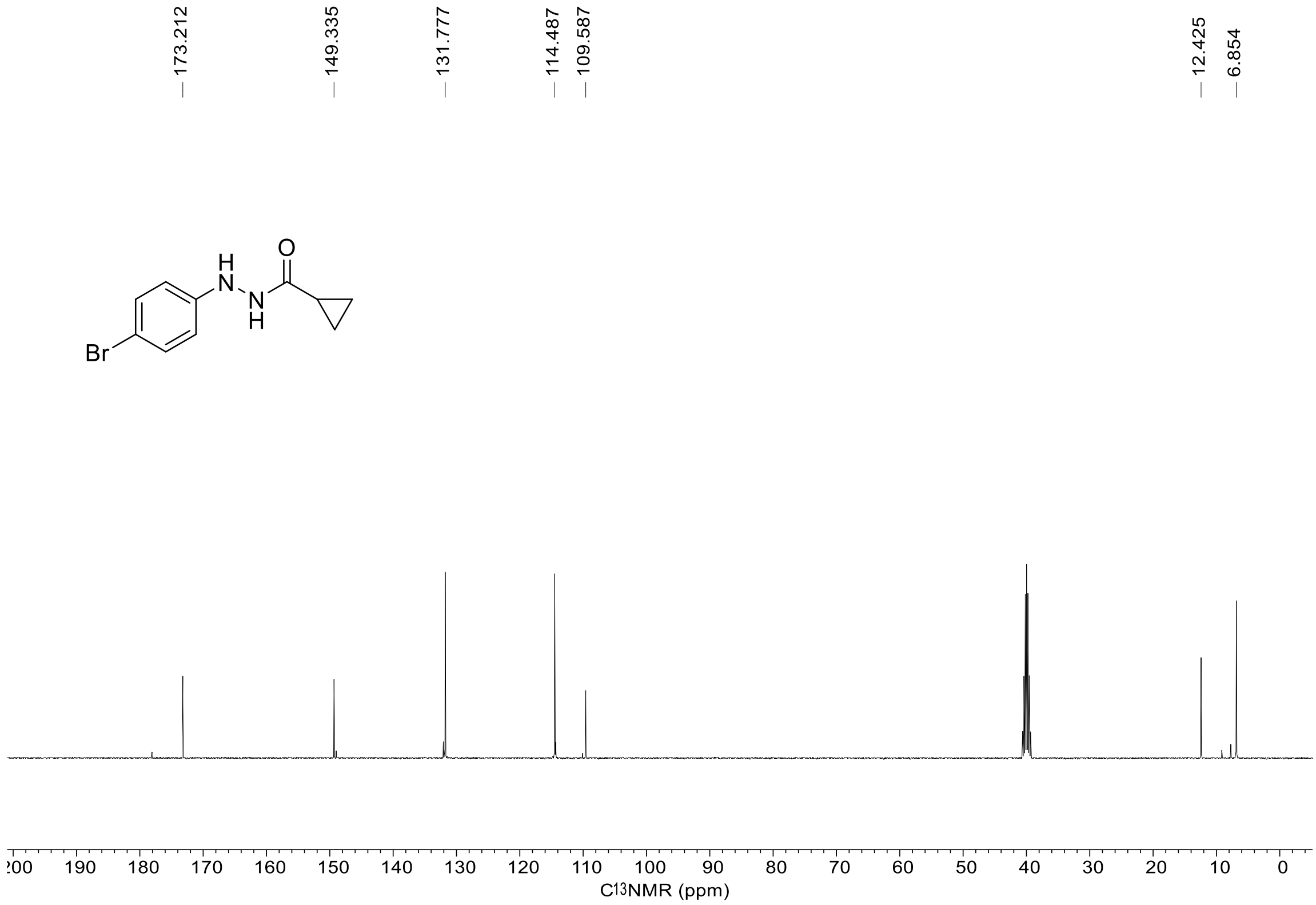

Figure S20 ^{13}C NMR spectrum of compound **10** (101 MHz, DMSO-d_6)

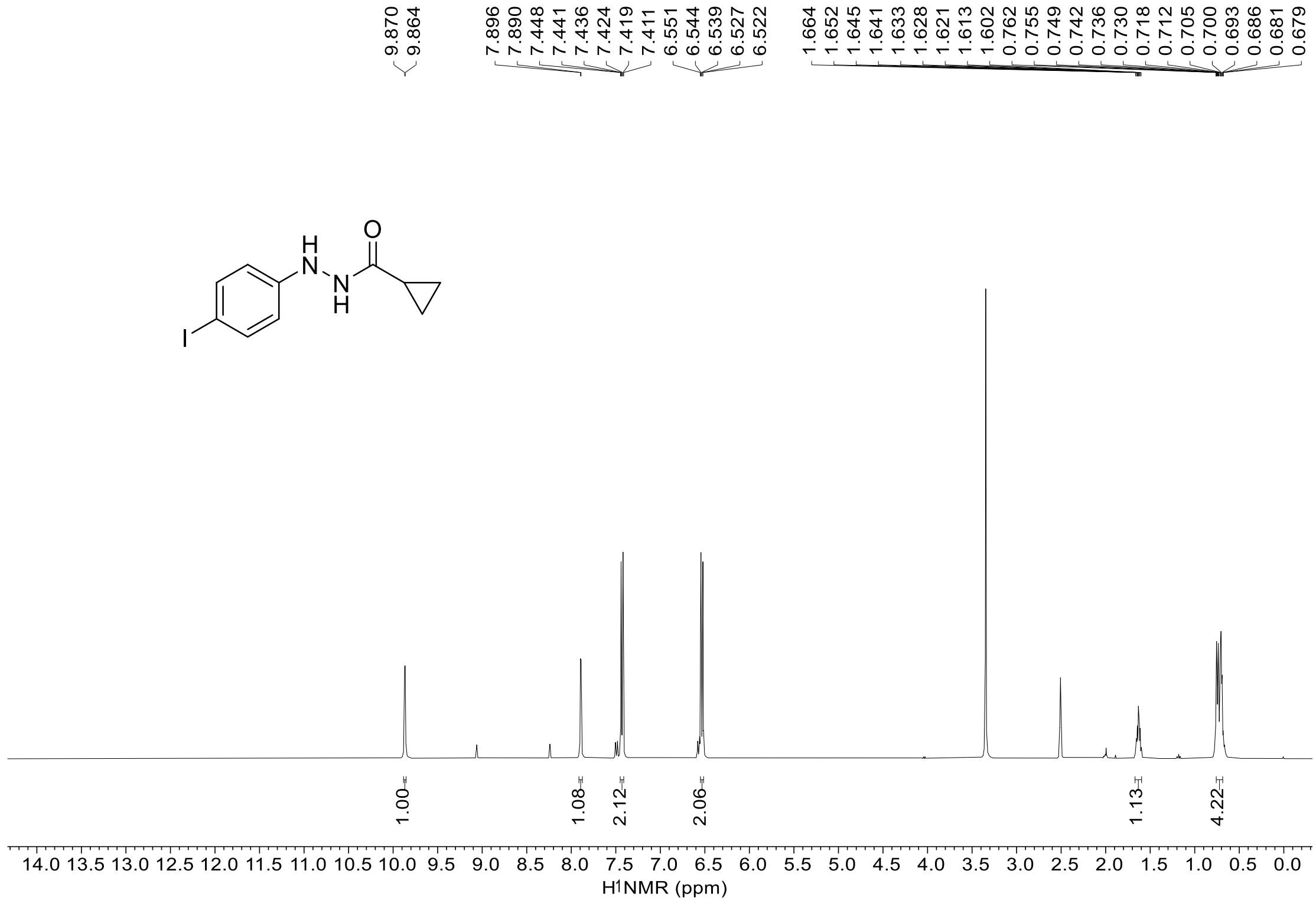

Figure S21 ^{1}H NMR spectrum of compound **11** (400 MHz, DMSO-d_6)

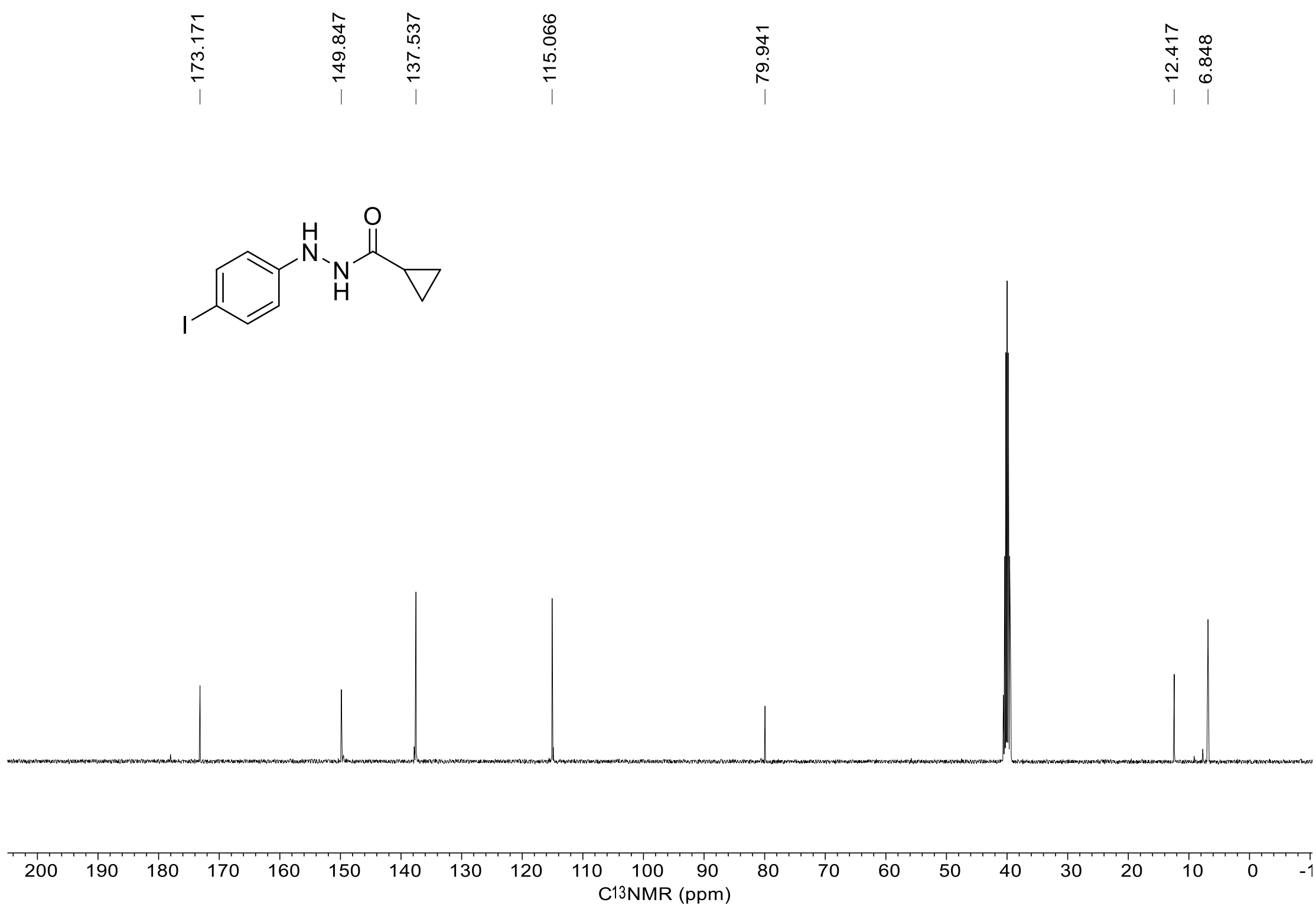

Figure S22 ^{13}C NMR spectrum of compound **11** (101 MHz, DMSO-d_6)

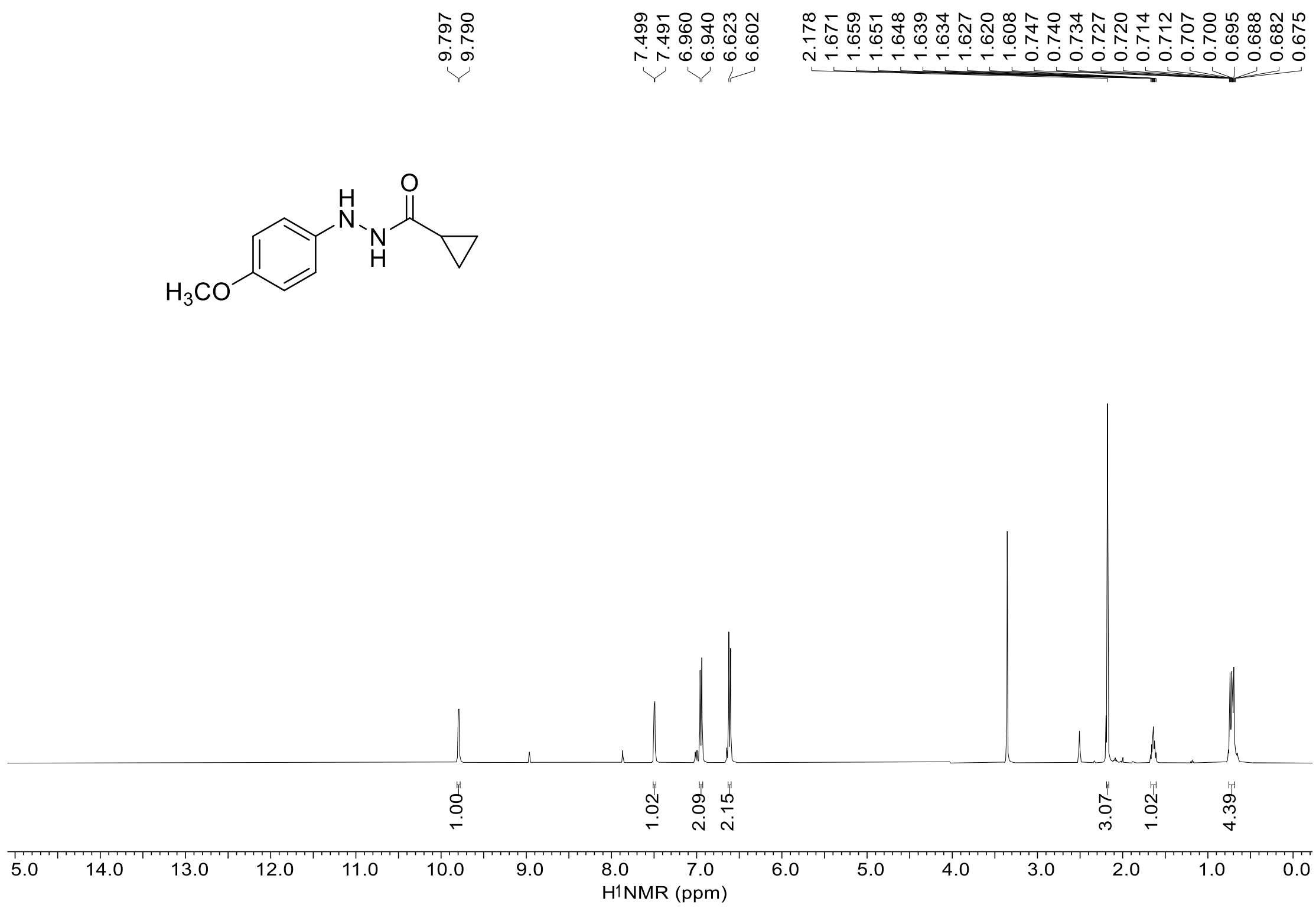

Figure S23 ^{1}H NMR spectrum of compound **12** (400 MHz, DMSO-d_6)

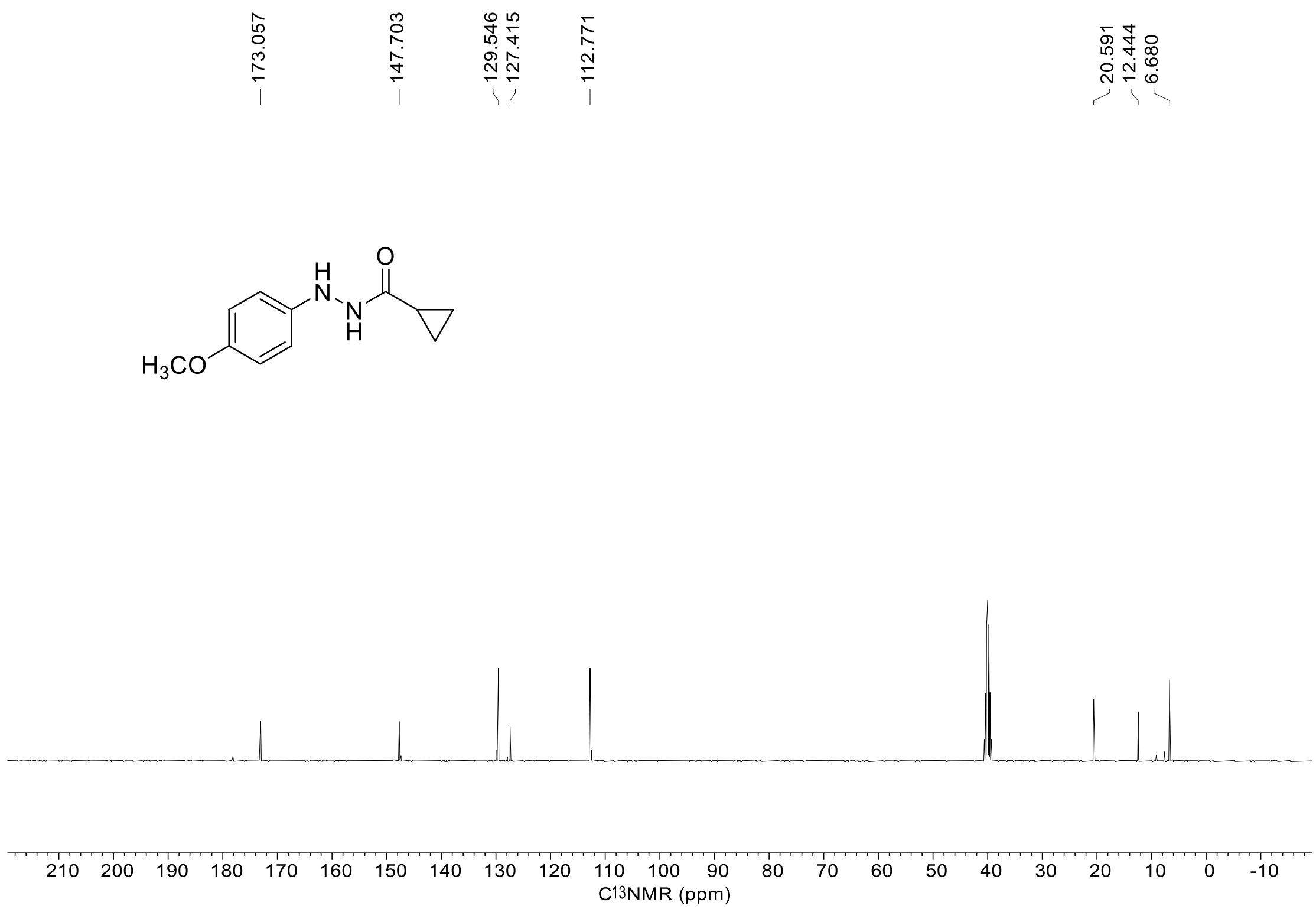

Figure S24 ^{13}C NMR spectrum of compound **12** (101 MHz, DMSO-d_6)

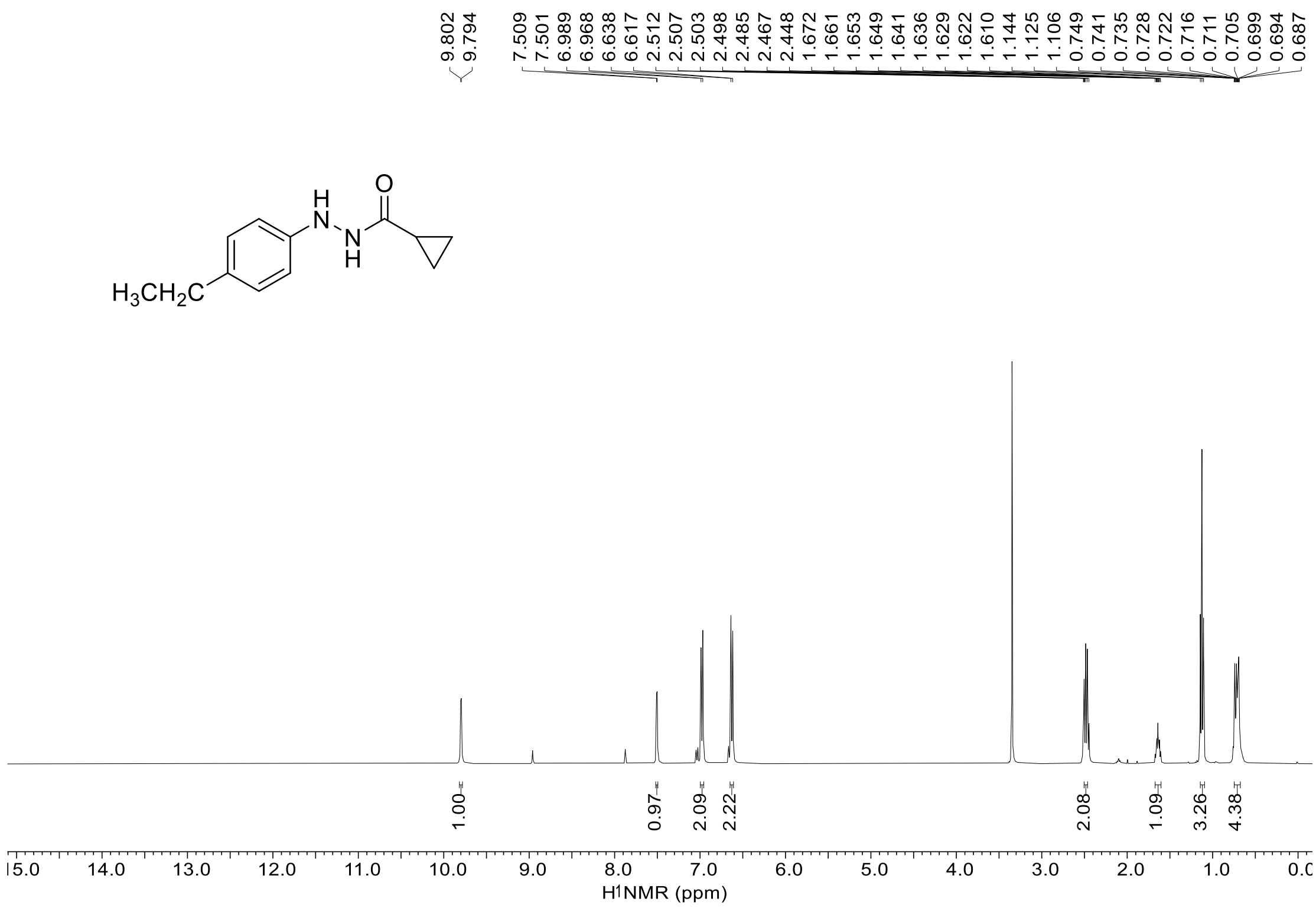

Figure S25 ^{1}H NMR spectrum of compound **13** (400 MHz, DMSO-d_6)

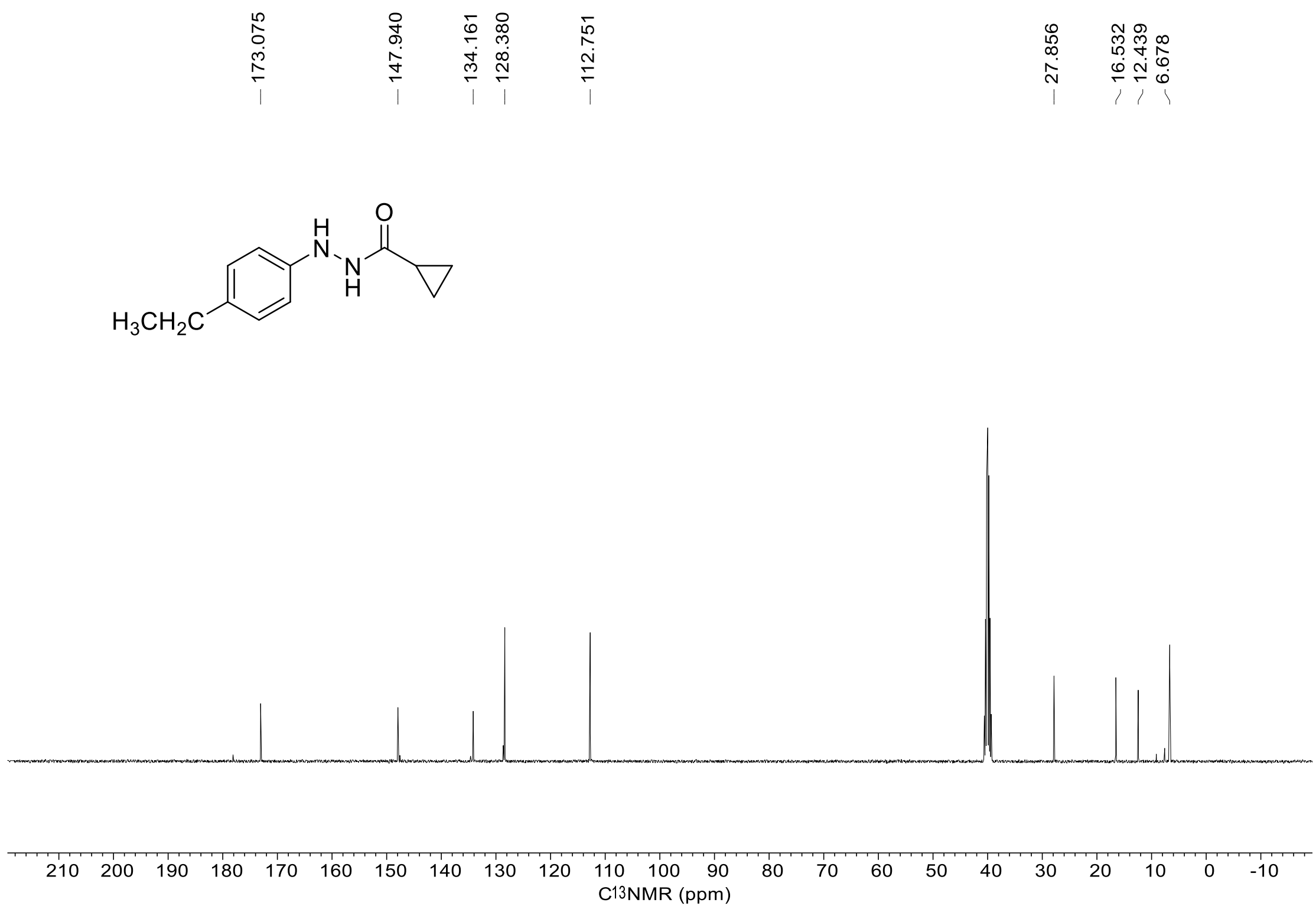

Figure S26 ^{13}C NMR spectrum of compound **13** (101 MHz, DMSO-d_6)

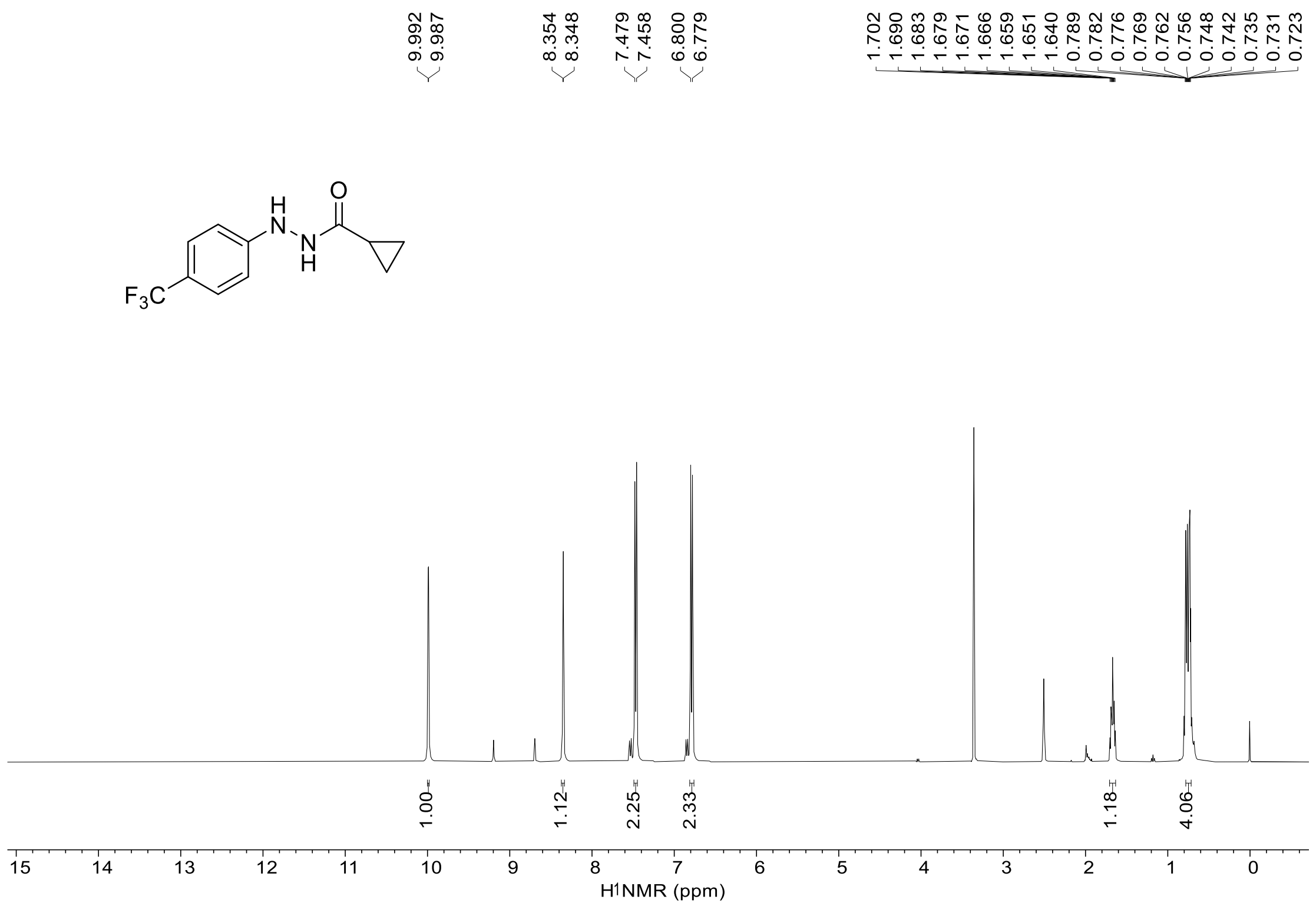

Figure S27 ^{1}H NMR spectrum of compound **14** (400 MHz, DMSO-d_6)

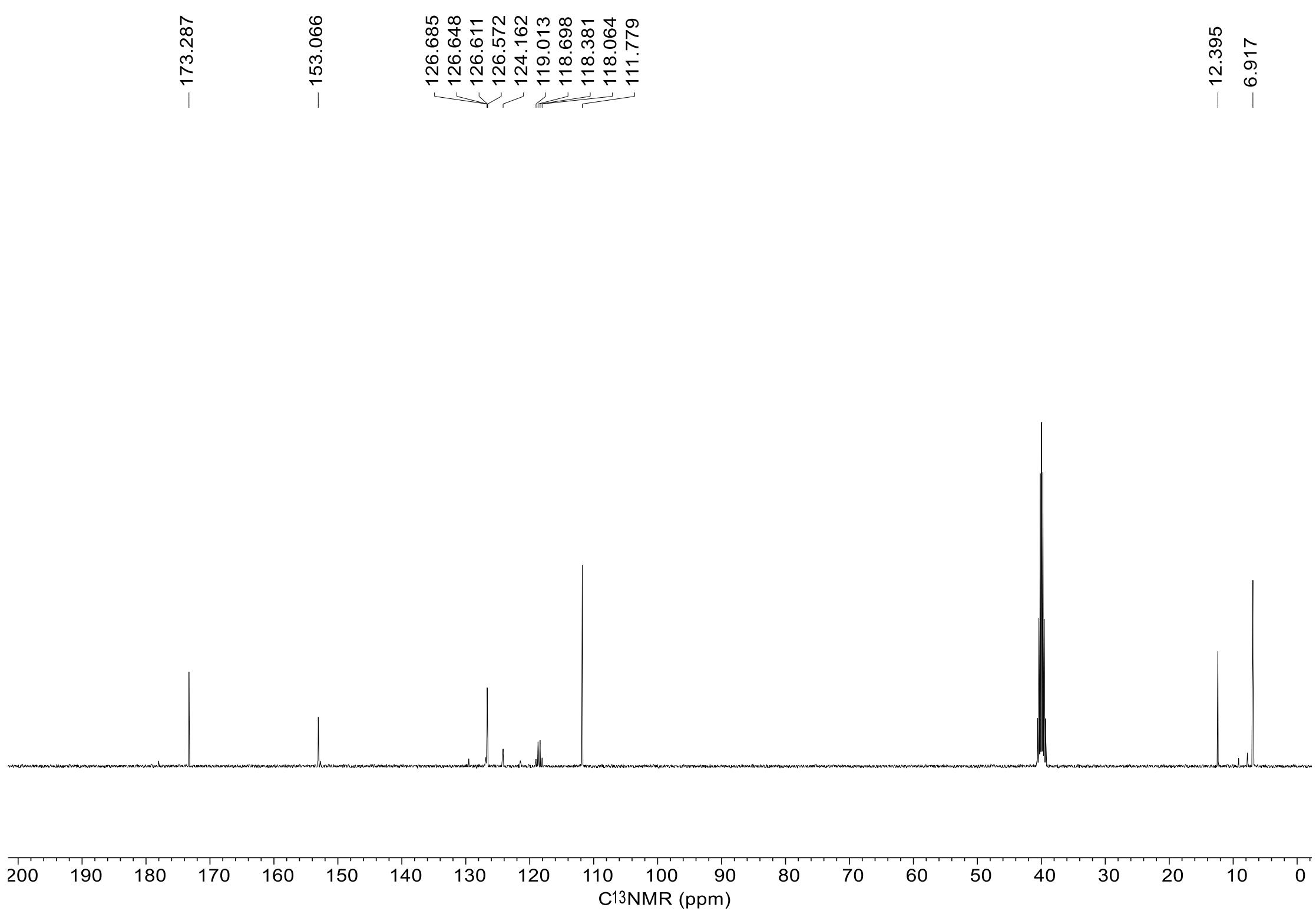

Figure S28 ^{13}C NMR spectrum of compound **14** (101 MHz, DMSO-d_6)

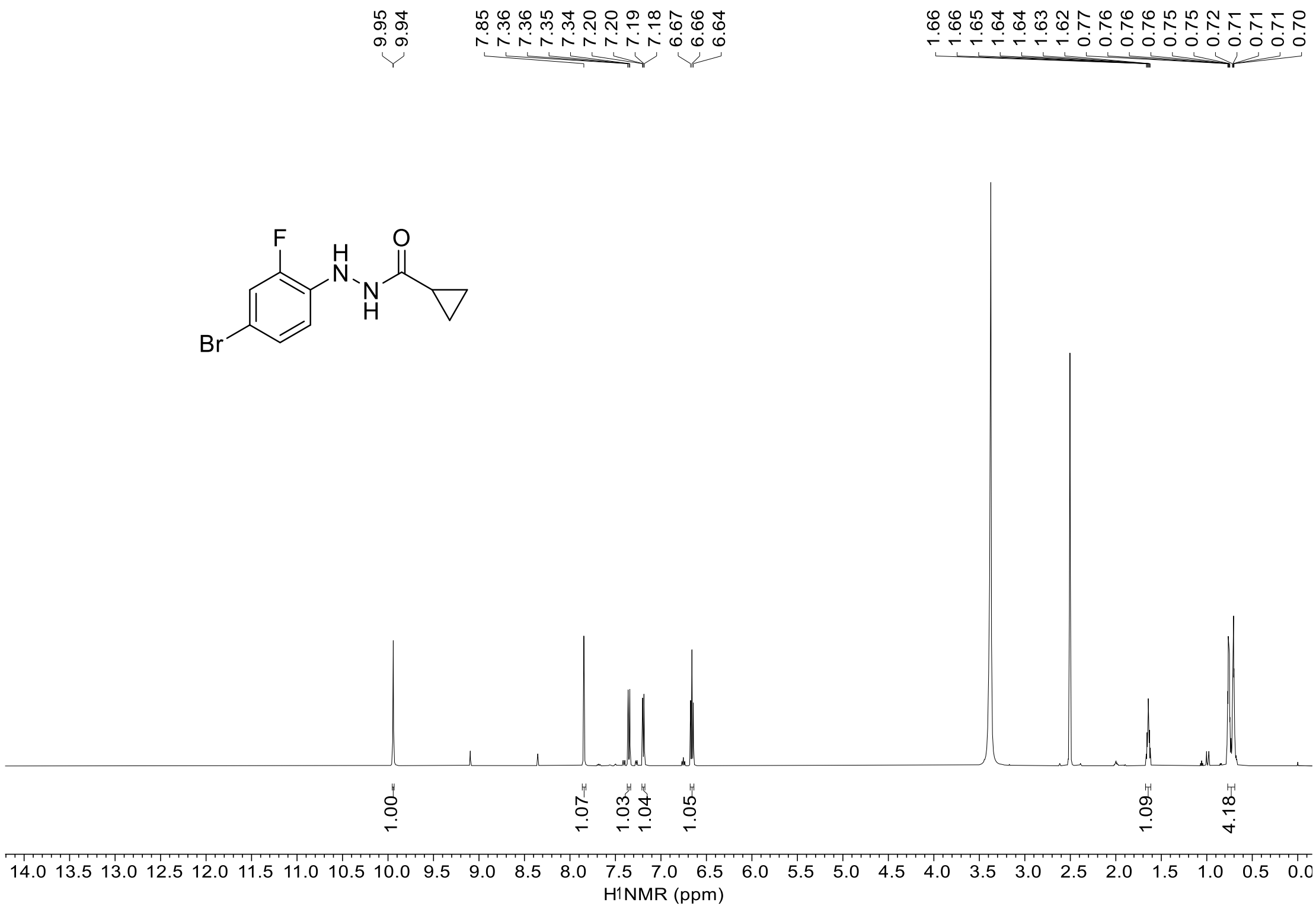

Figure S29 ^{1}H NMR spectrum of compound **15** (600 MHz, DMSO-d_6)

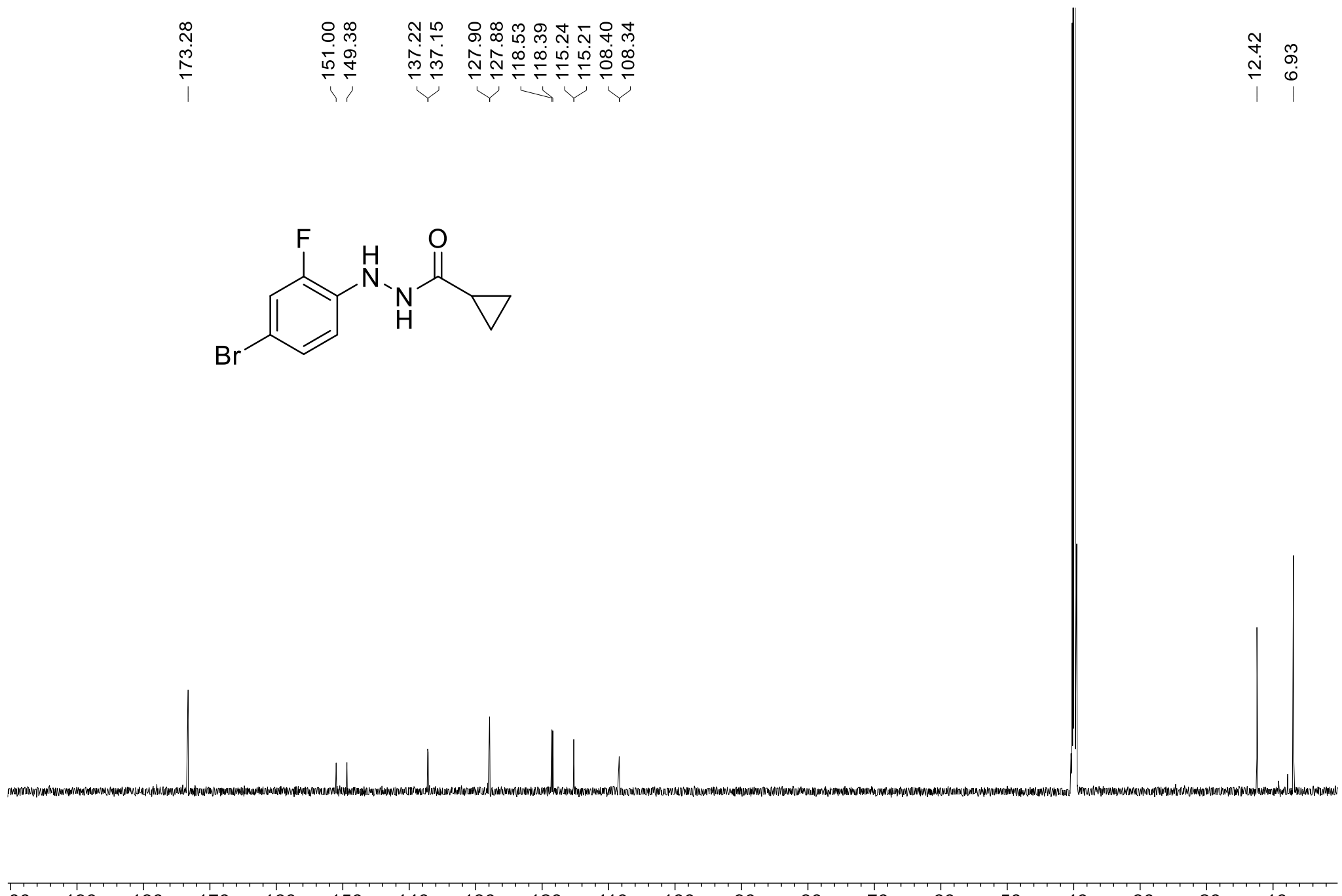

Figure S30 ^{13}C NMR spectrum of compound **15** (151 MHz, DMSO-d_6)

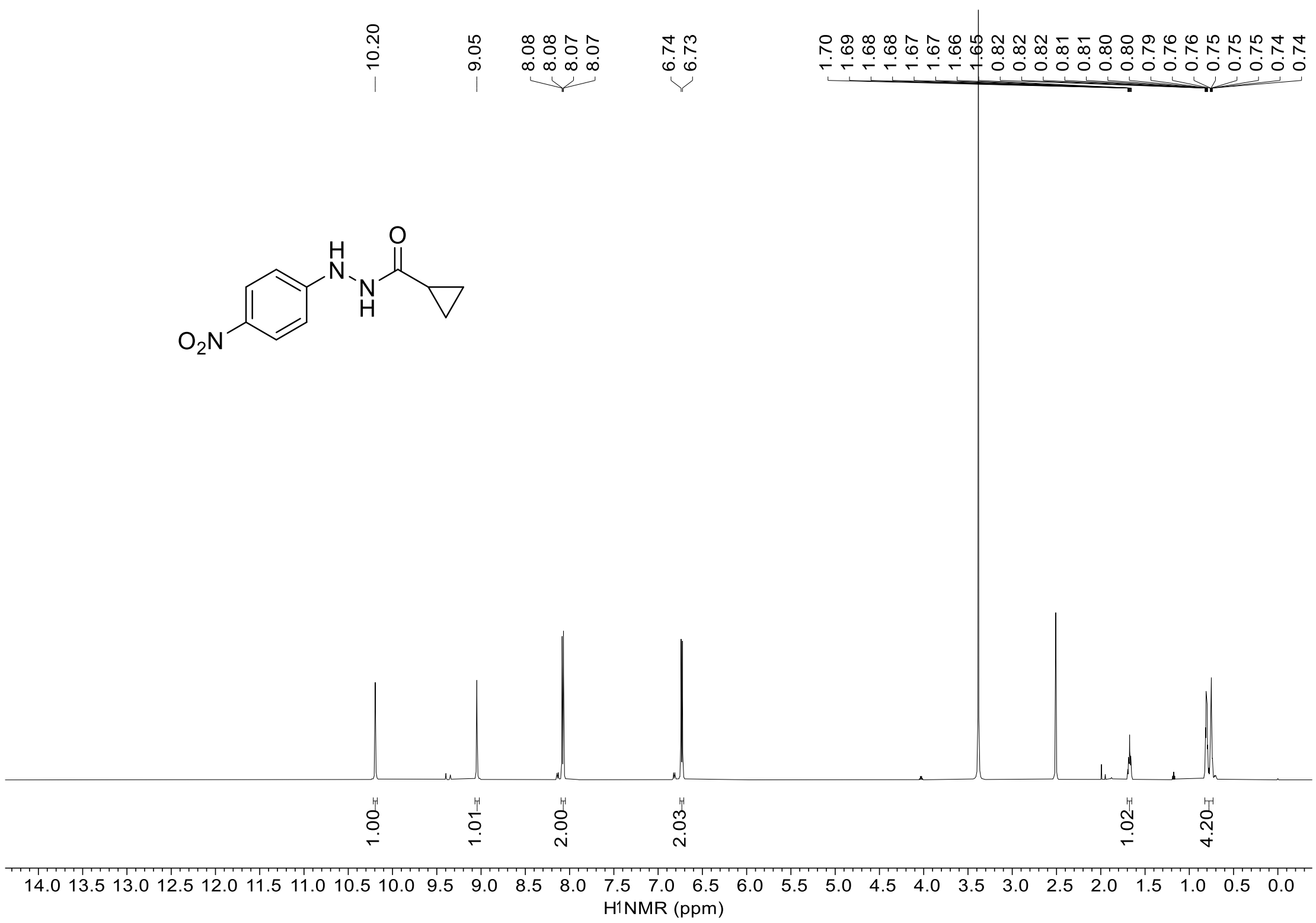

Figure S31 ^{1}H NMR spectrum of compound **16** (600 MHz, DMSO-d_6)

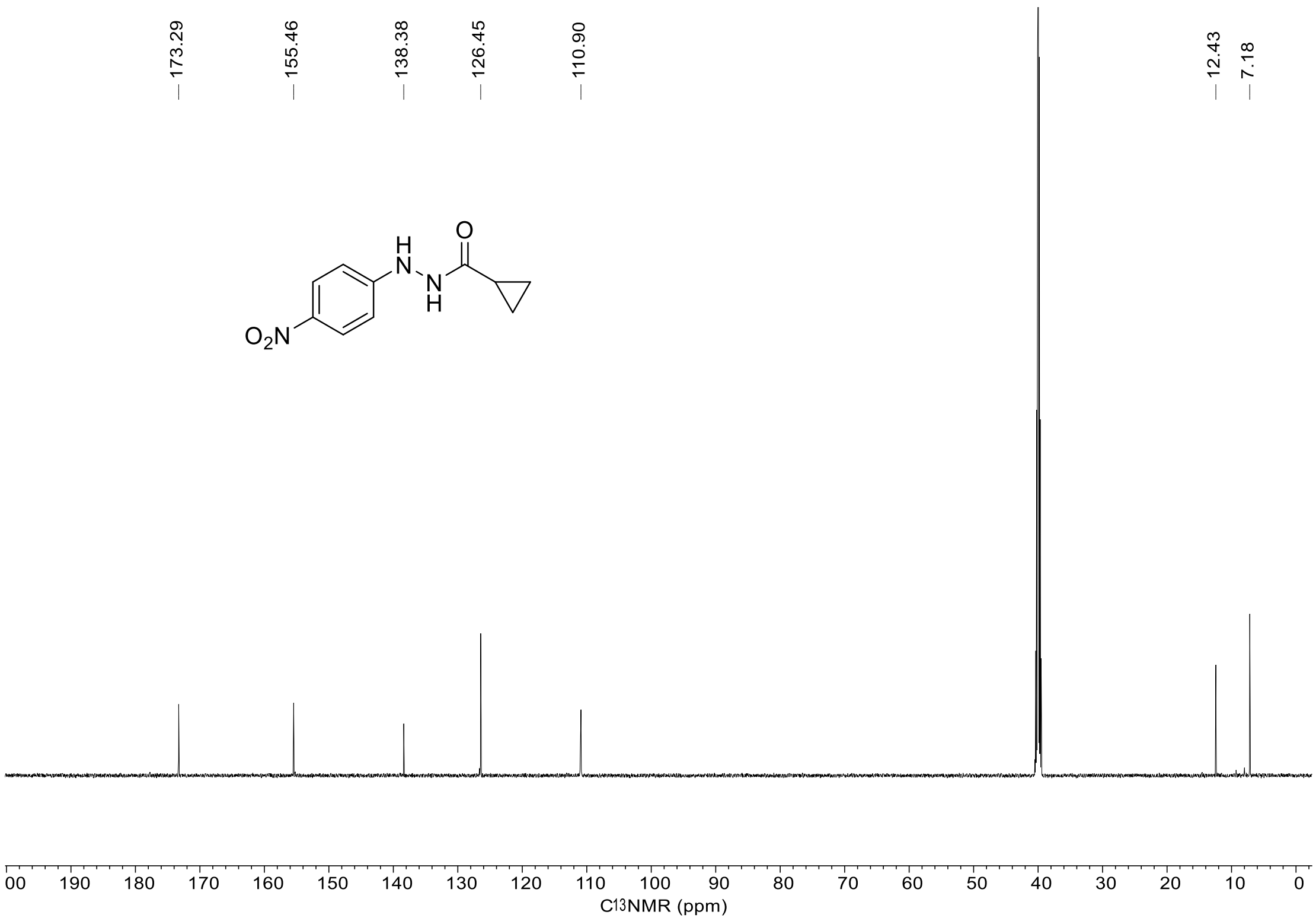

Figure S32 ^{13}C NMR spectrum of compound **16** (151 MHz, DMSO-d_6)

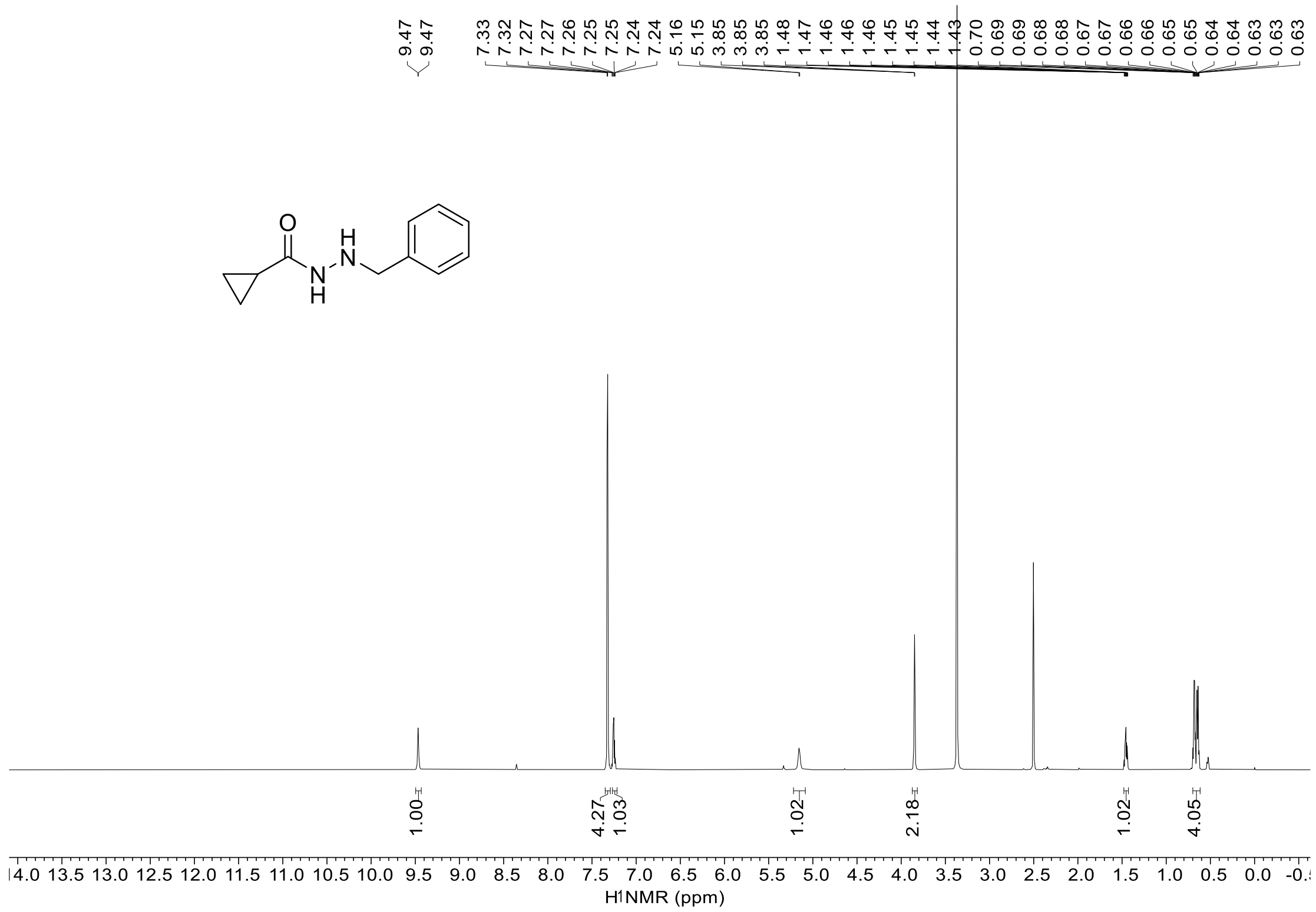

Figure S33 ^{1}H NMR spectrum of compound **17** (600 MHz, DMSO-d_6)

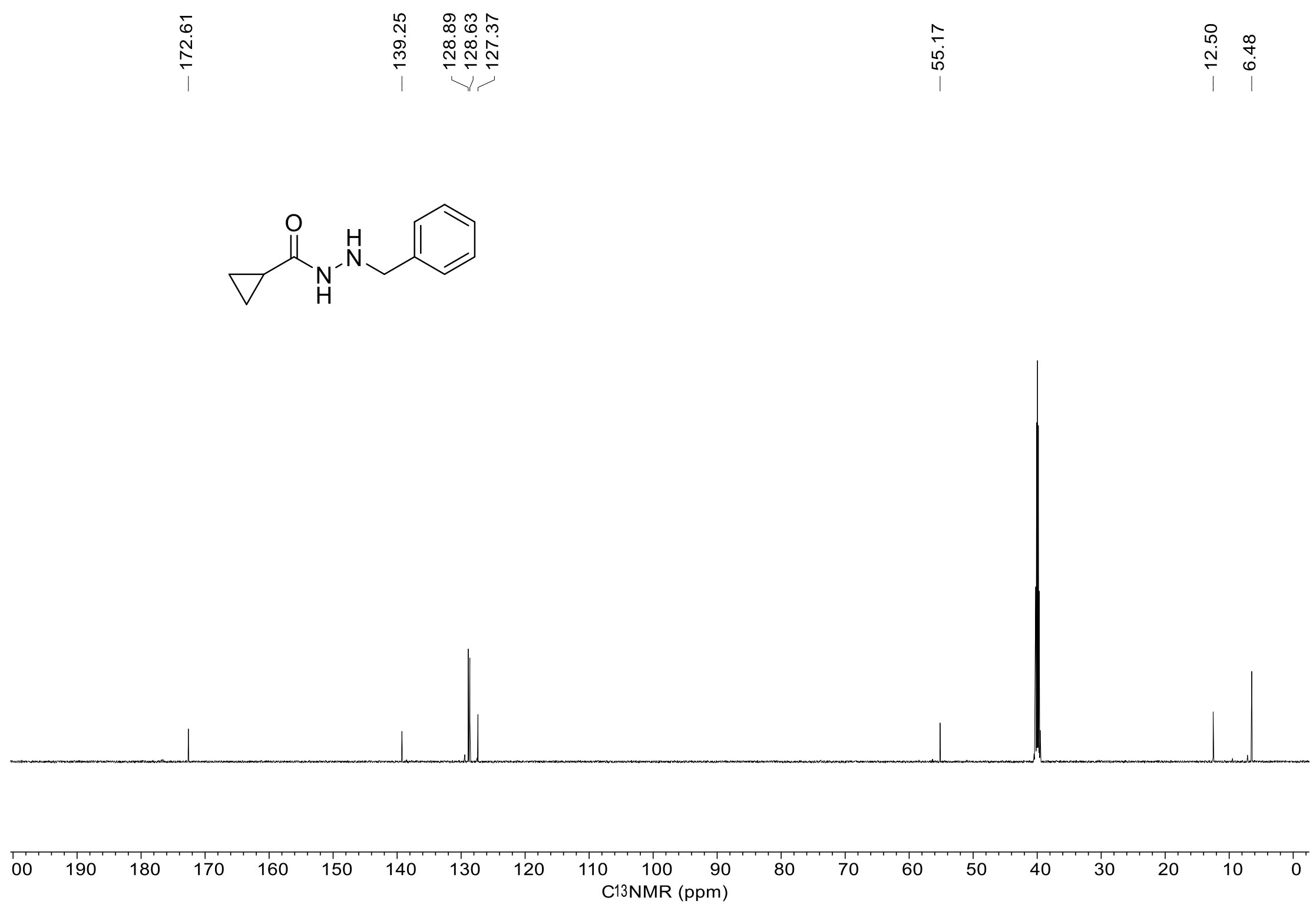

Figure S34 ^{13}C NMR spectrum of compound **17** (151 MHz, DMSO-d_6)

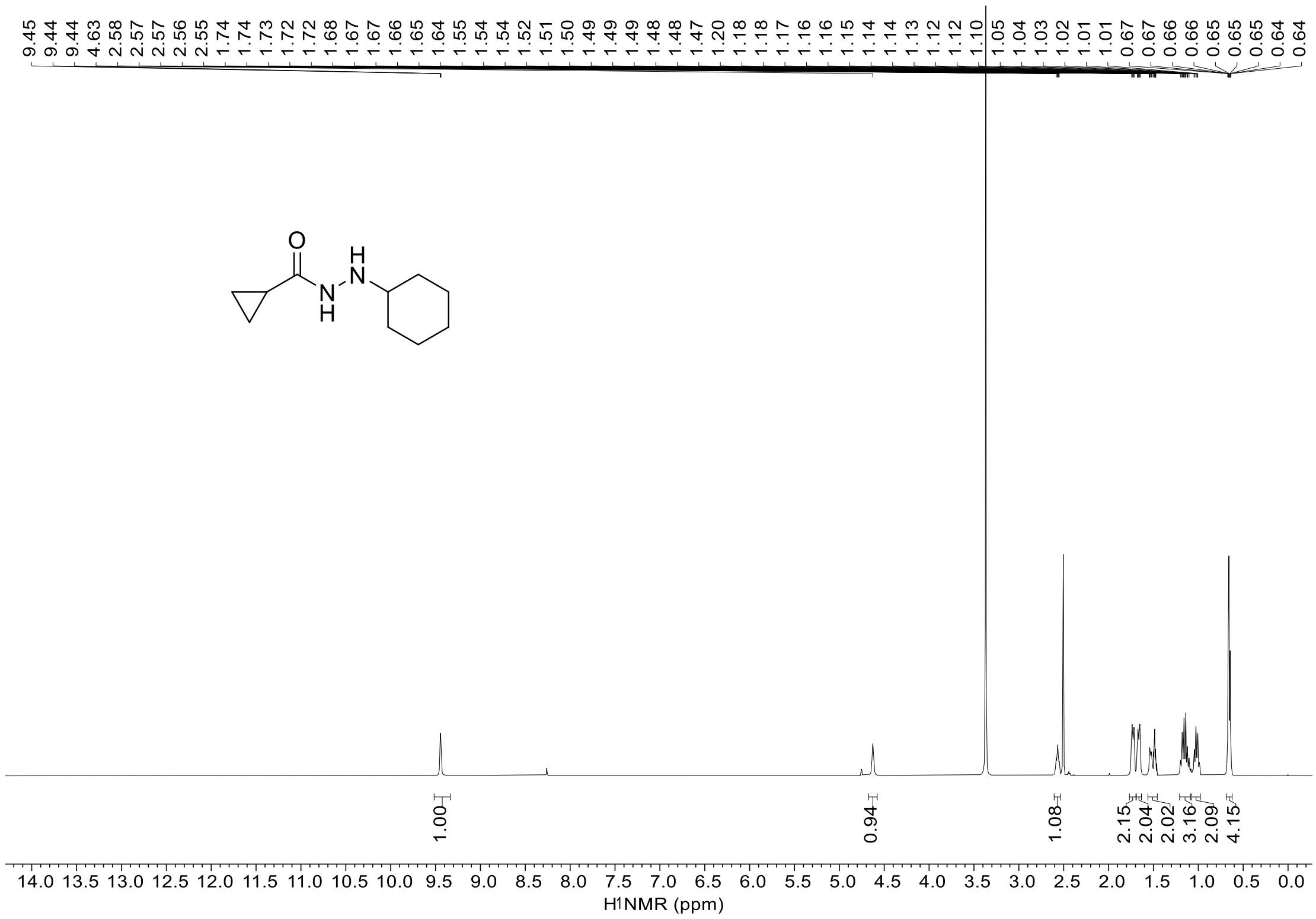

Figure S35 ^{1}H NMR spectrum of compound **18** (600 MHz, DMSO-d_6)

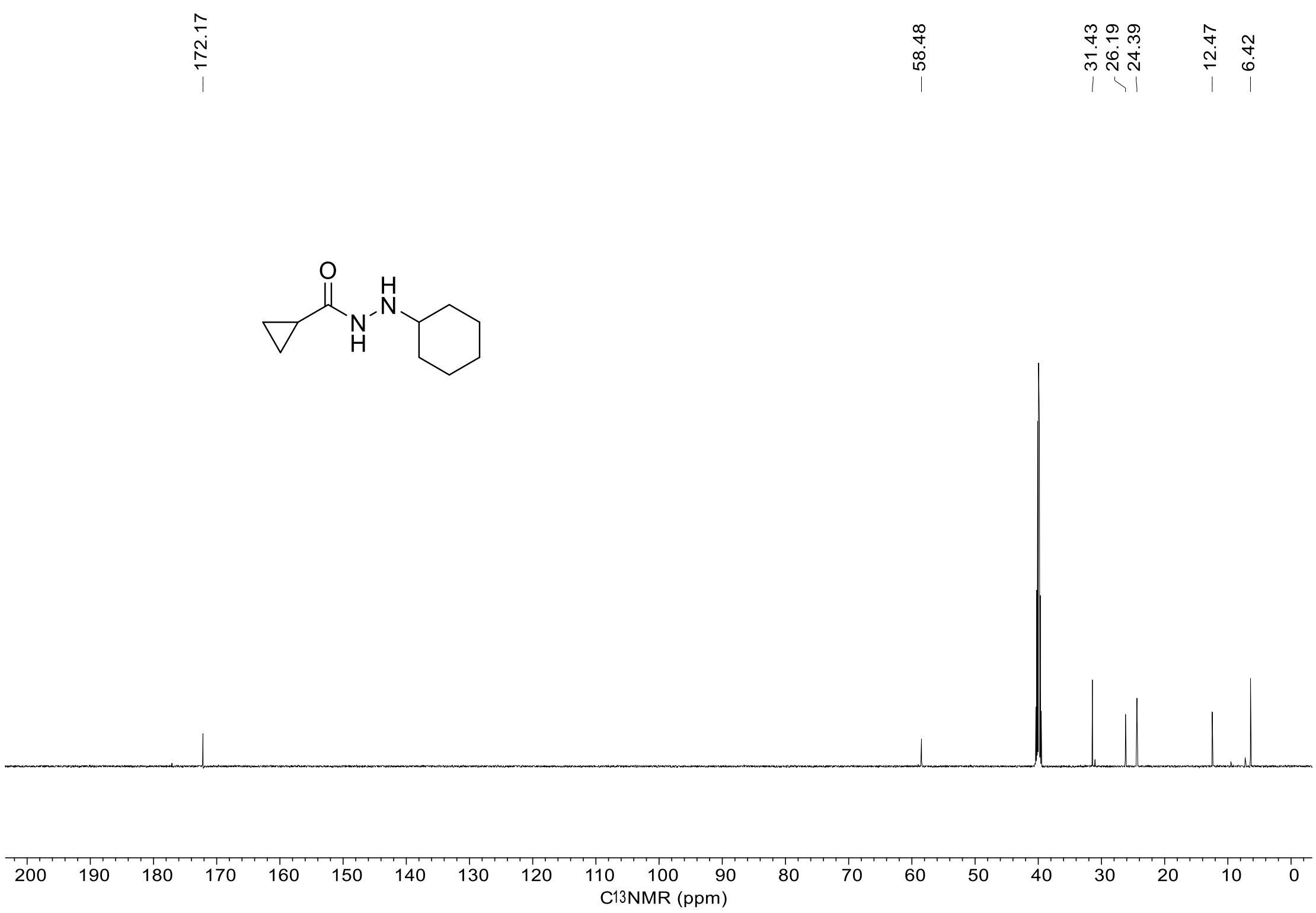

Figure S36 ^{13}C NMR spectrum of compound **18** (151 MHz, DMSO-d_6)

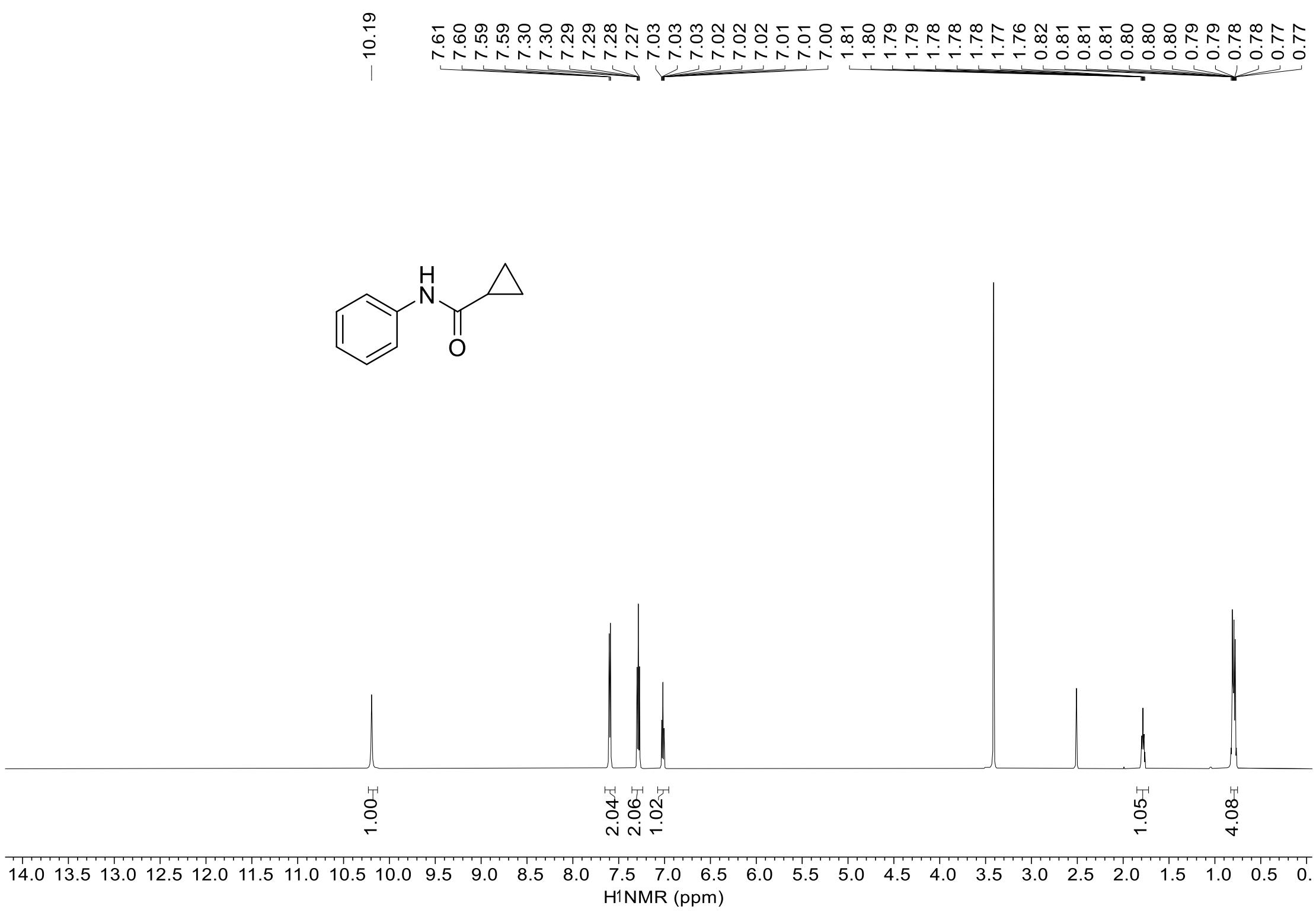

Figure S37 ^{1}H NMR spectrum of compound **19** (600 MHz, DMSO-d_6)

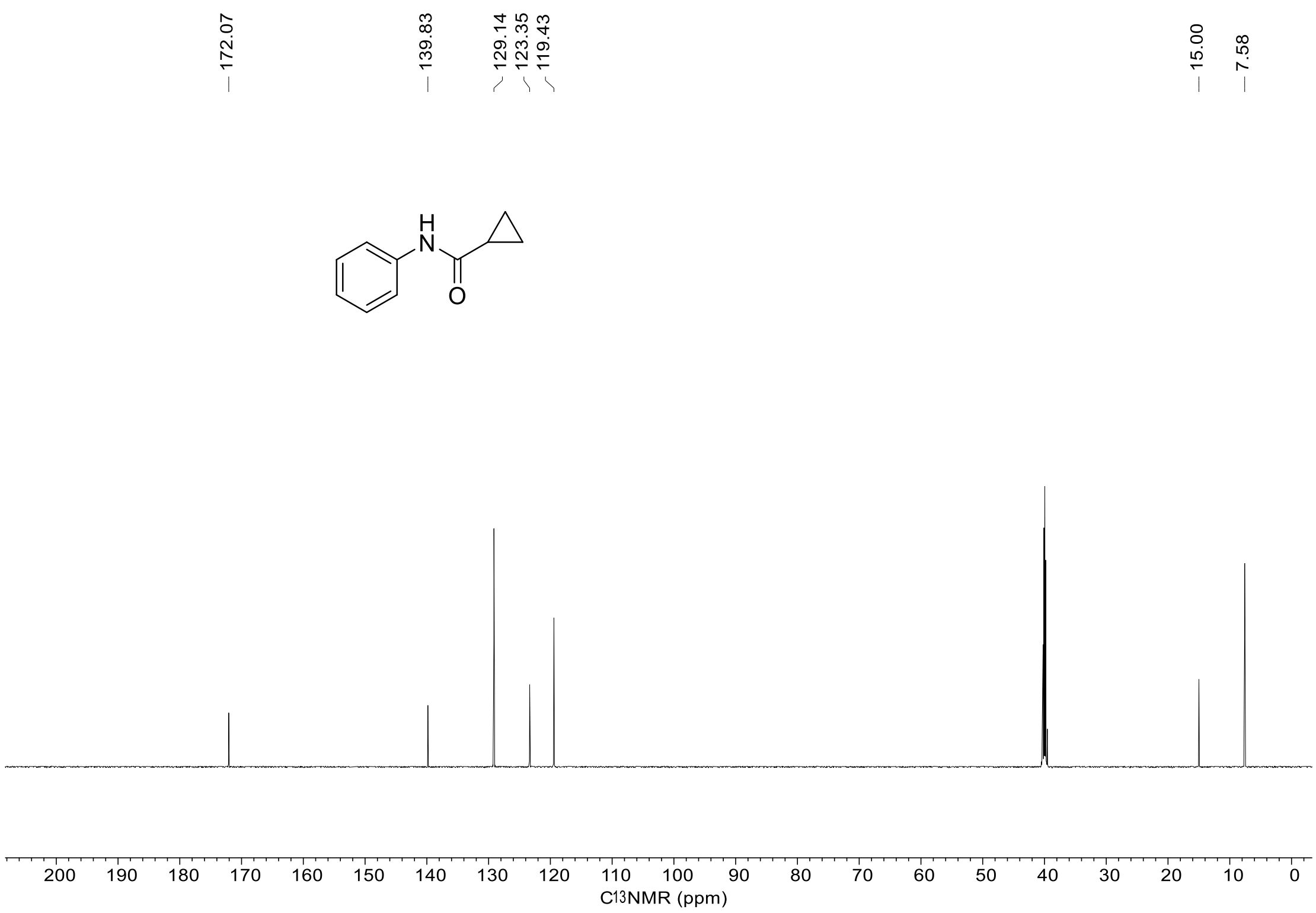

Figure S38 ^{13}C NMR spectrum of compound **19** (151 MHz, DMSO-d_6)

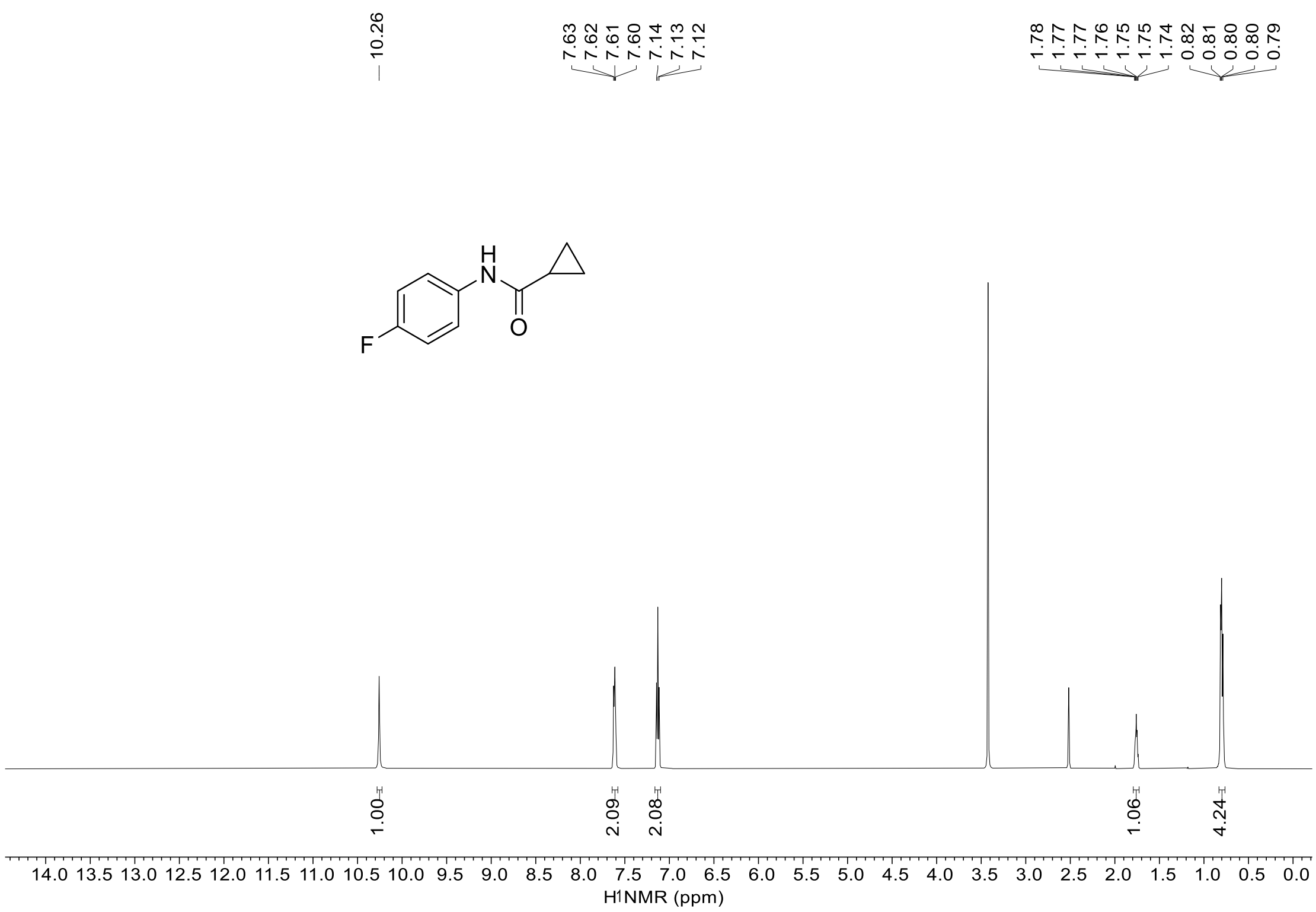

Figure S39 ^{1}H NMR spectrum of compound **20** (600 MHz, DMSO-d_6)

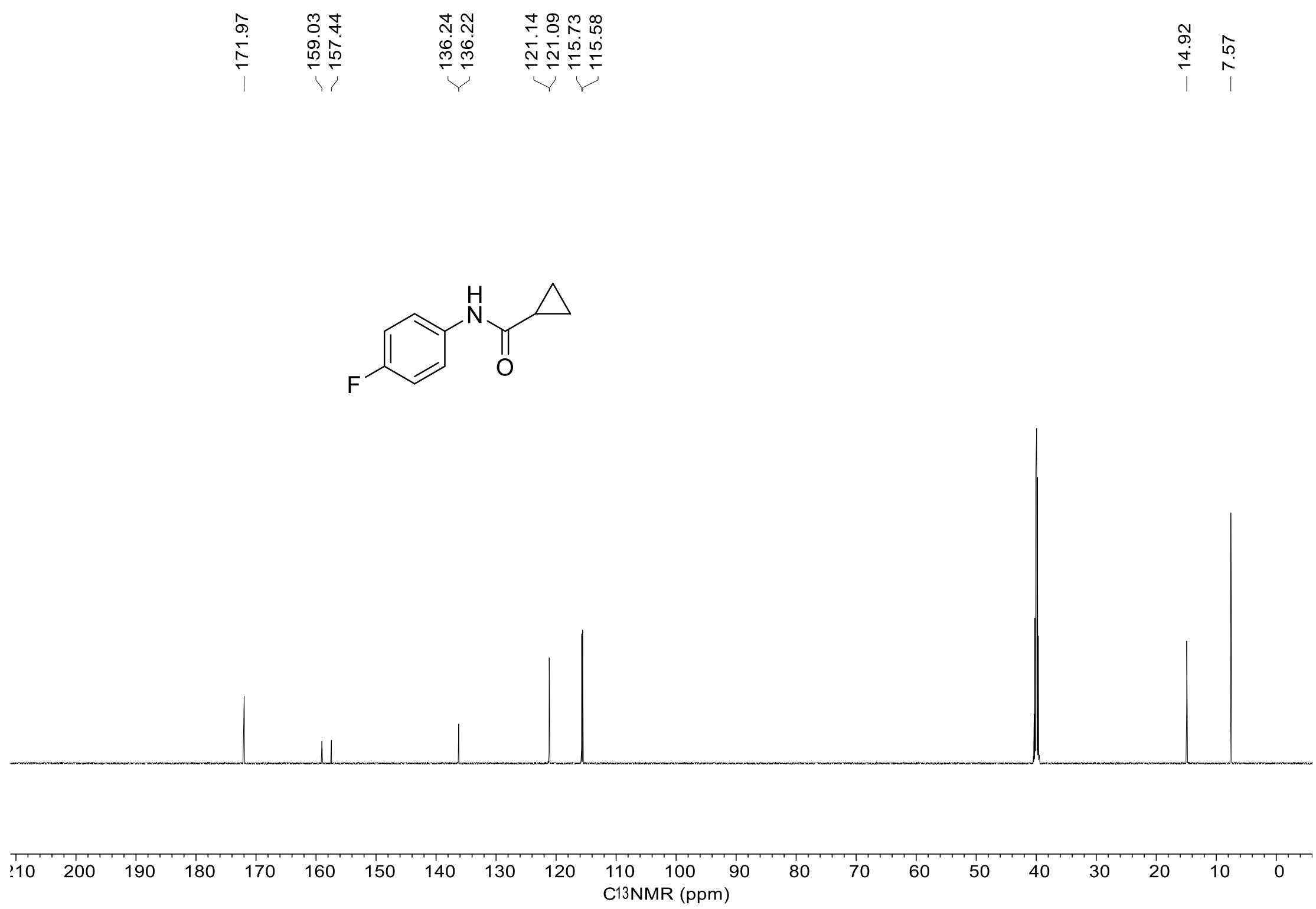

Figure S40 ^{13}C NMR spectrum of compound **20** (151 MHz, DMSO-d_6)

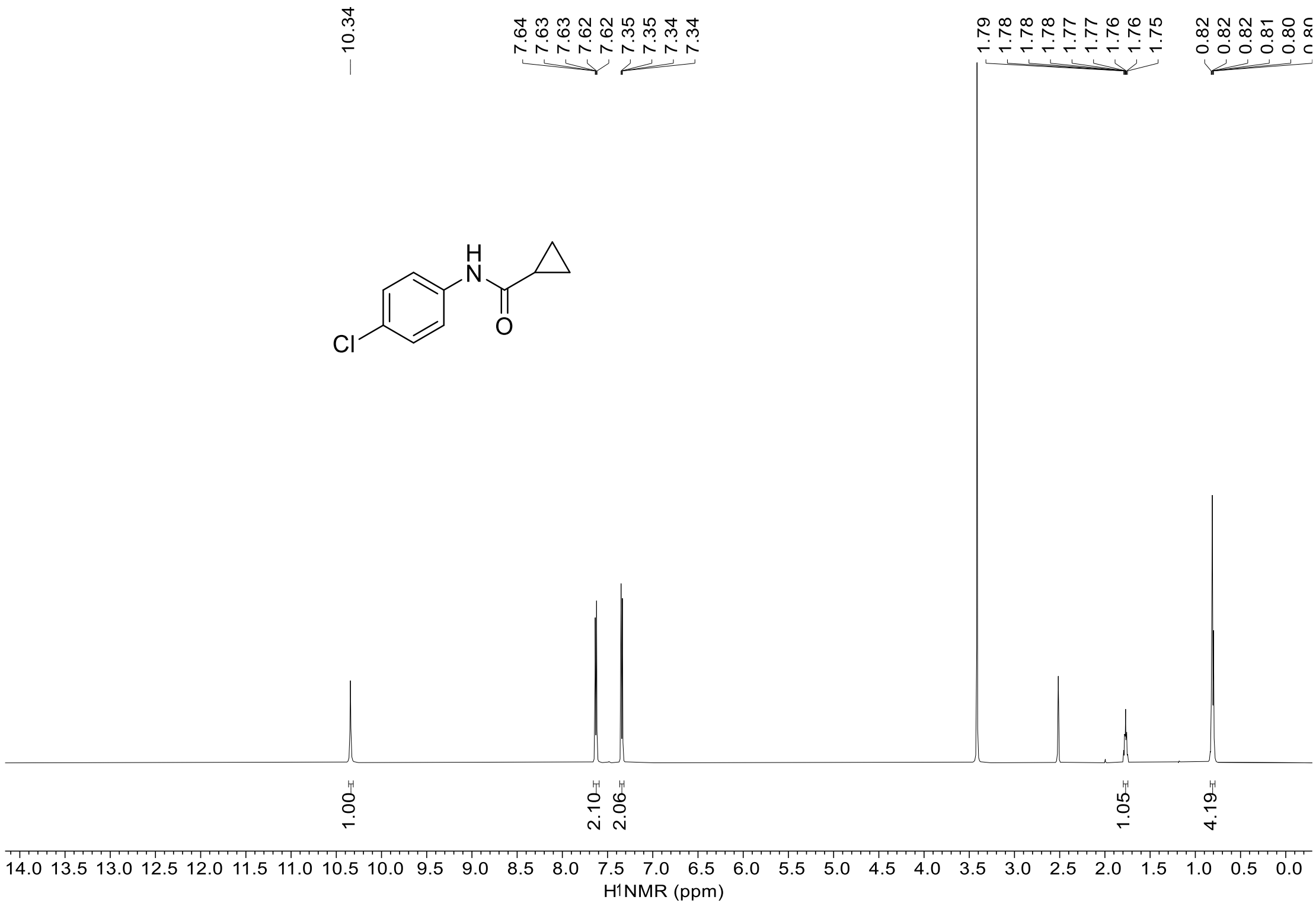

Figure S41 ^{1}H NMR spectrum of compound **21** (600 MHz, DMSO-d_6)

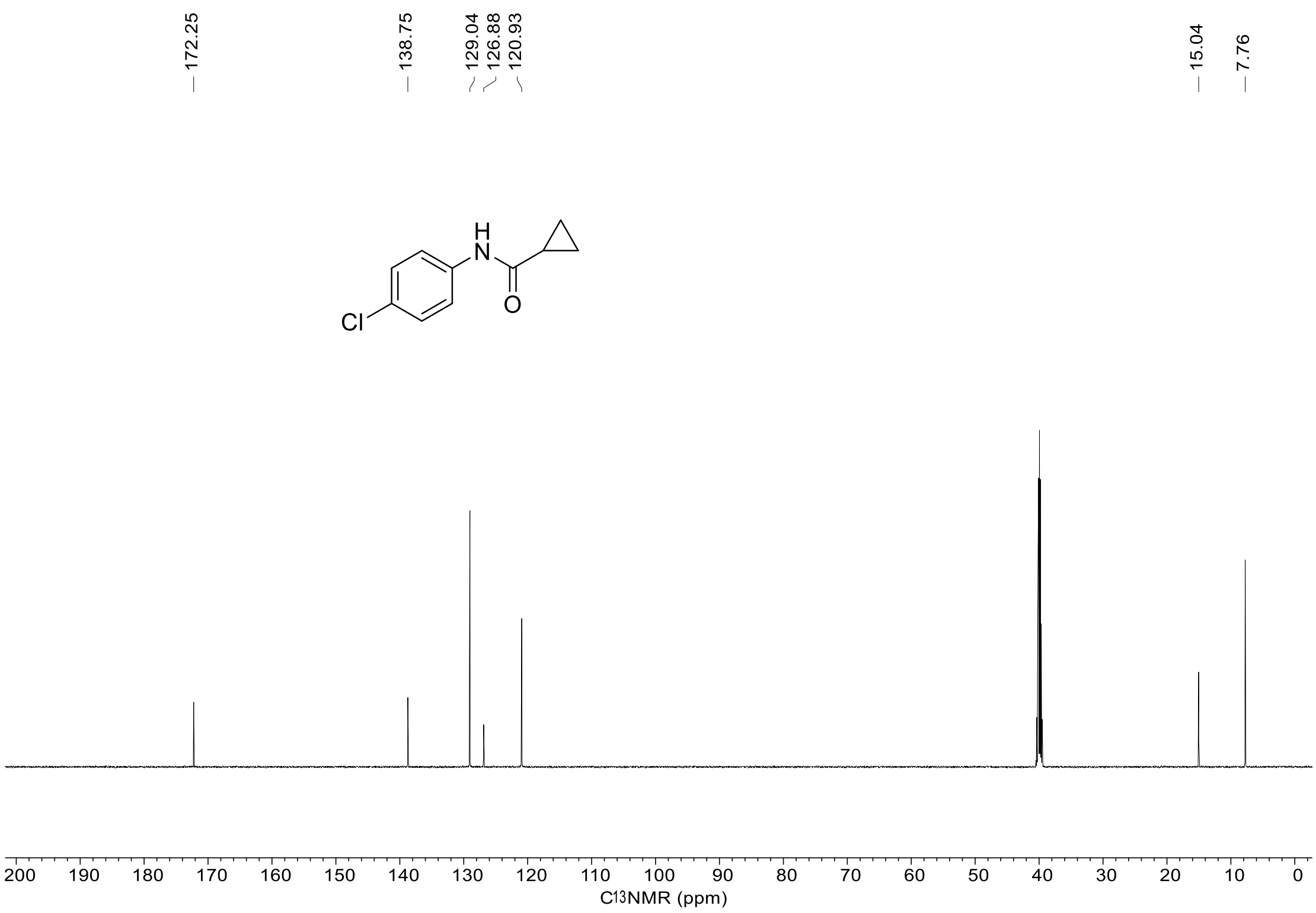

Figure S42 ^{13}C NMR spectrum of compound **21** (151 MHz, DMSO-d_6)

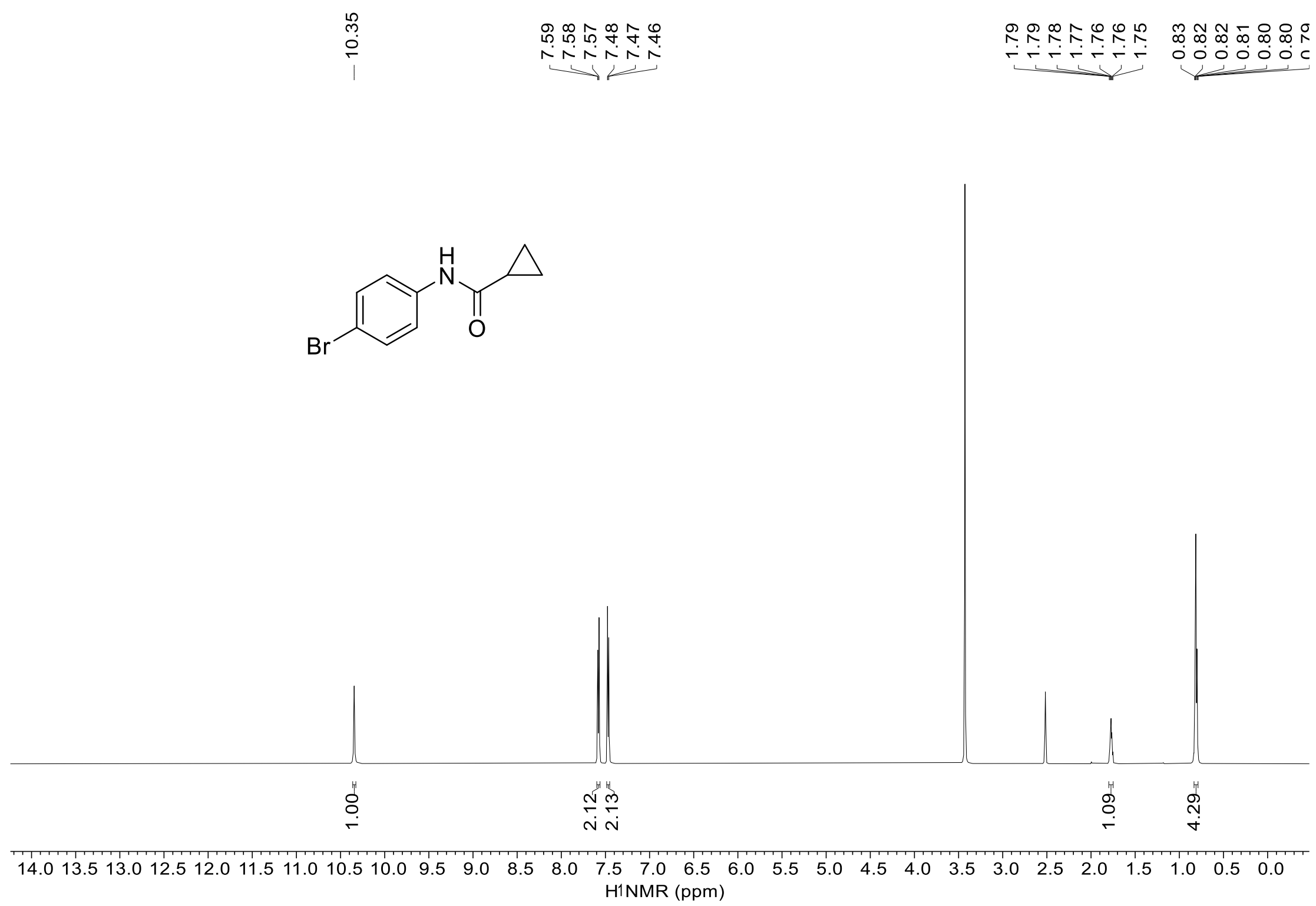

Figure S43 ^{1}H NMR spectrum of compound **22** (600 MHz, DMSO-d_6)

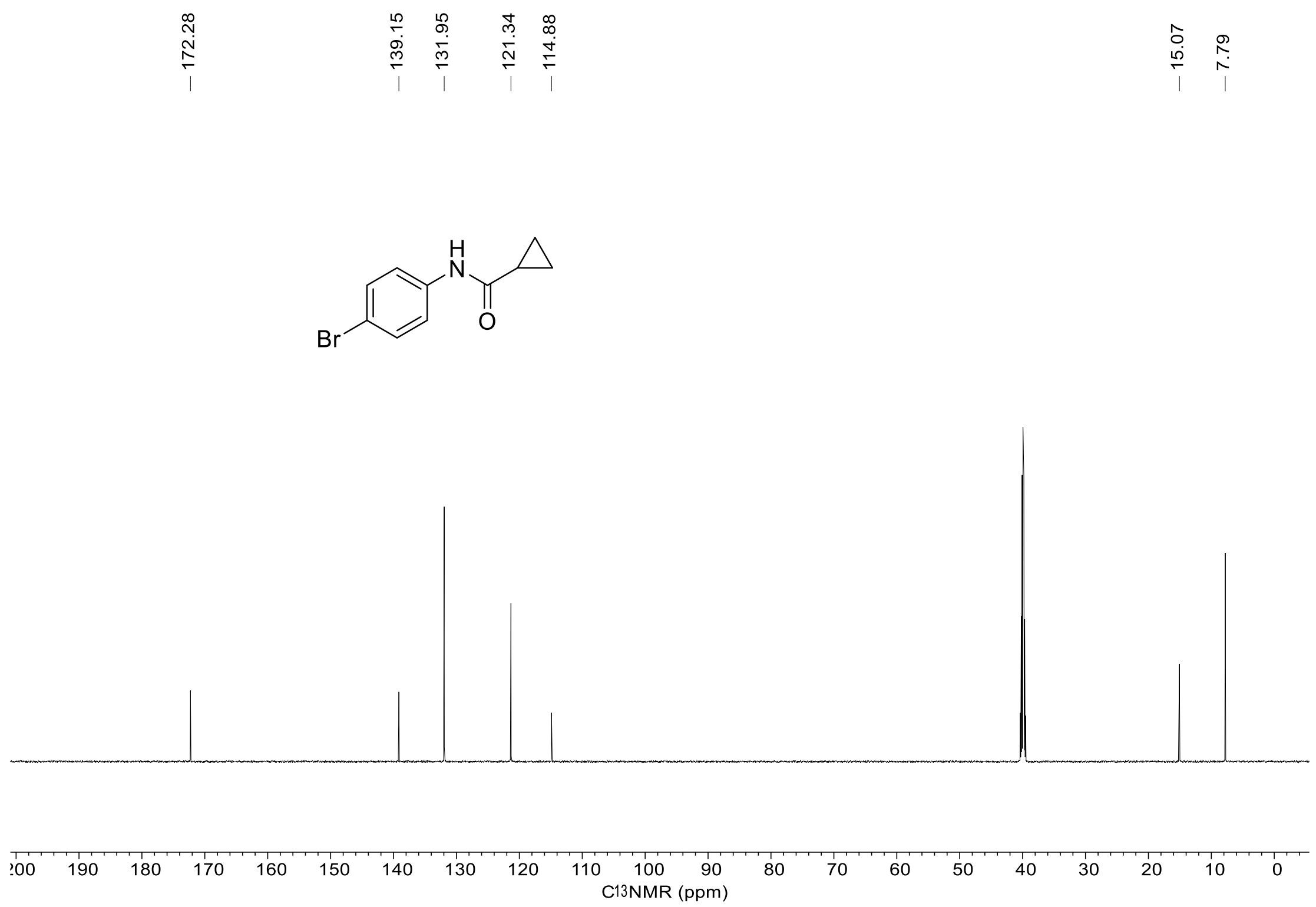

Figure S44 ^{13}C NMR spectrum of compound **22** (151 MHz, DMSO-d_6)

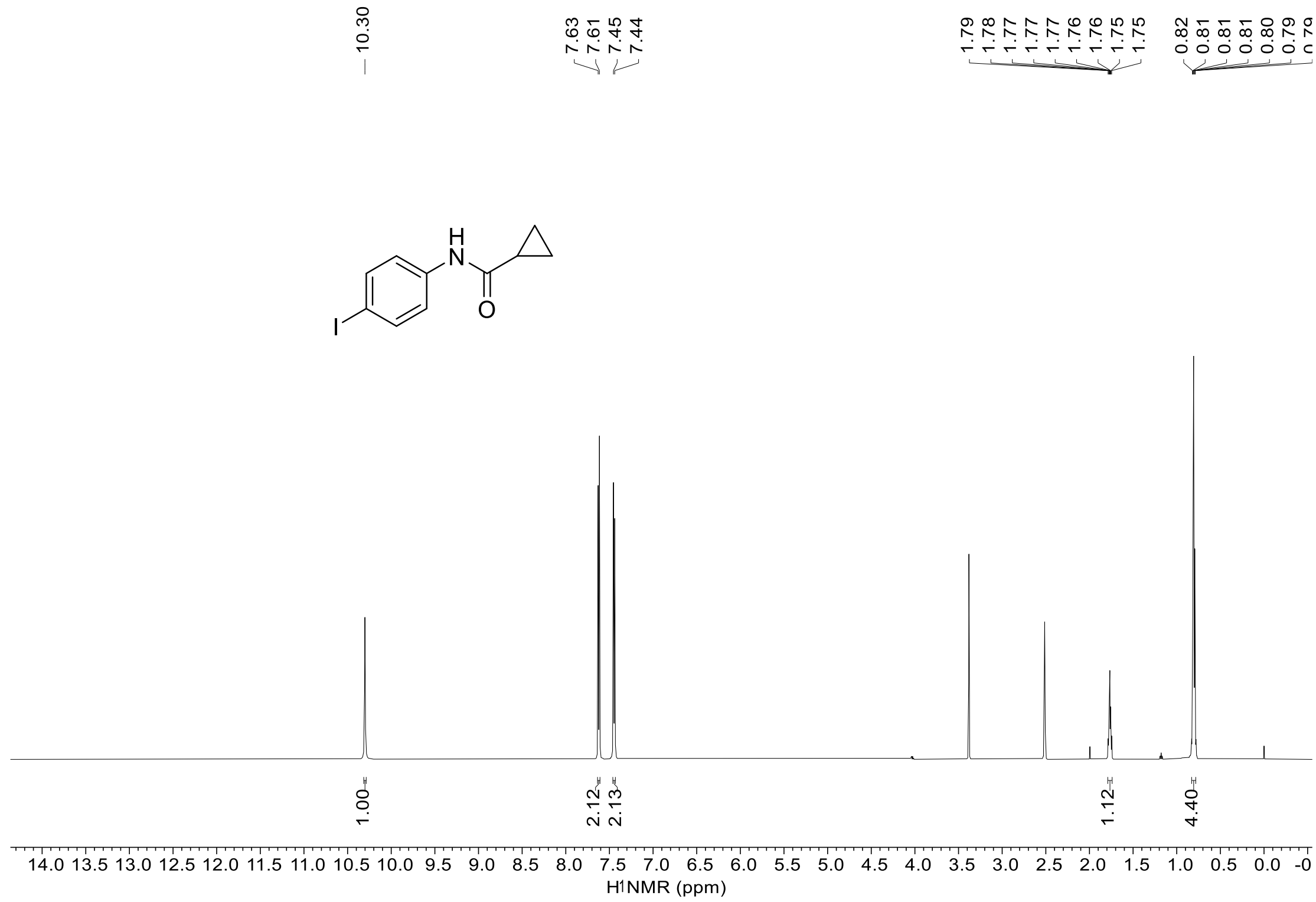

Figure S45 ^{1}H NMR spectrum of compound **23** (600 MHz, DMSO-d_6)

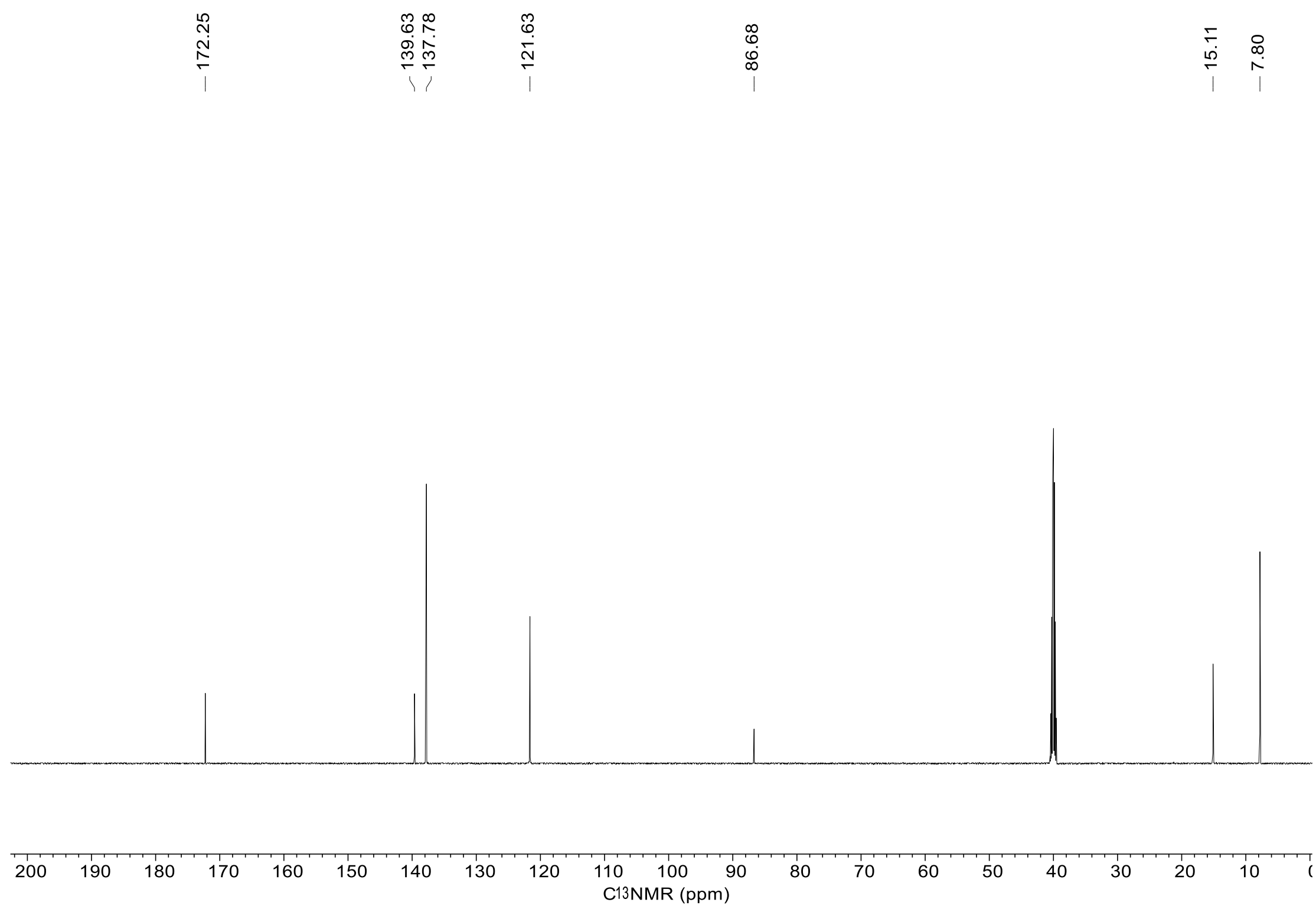

Figure S46 ^{13}C NMR spectrum of compound **23** (151 MHz, DMSO-d_6)

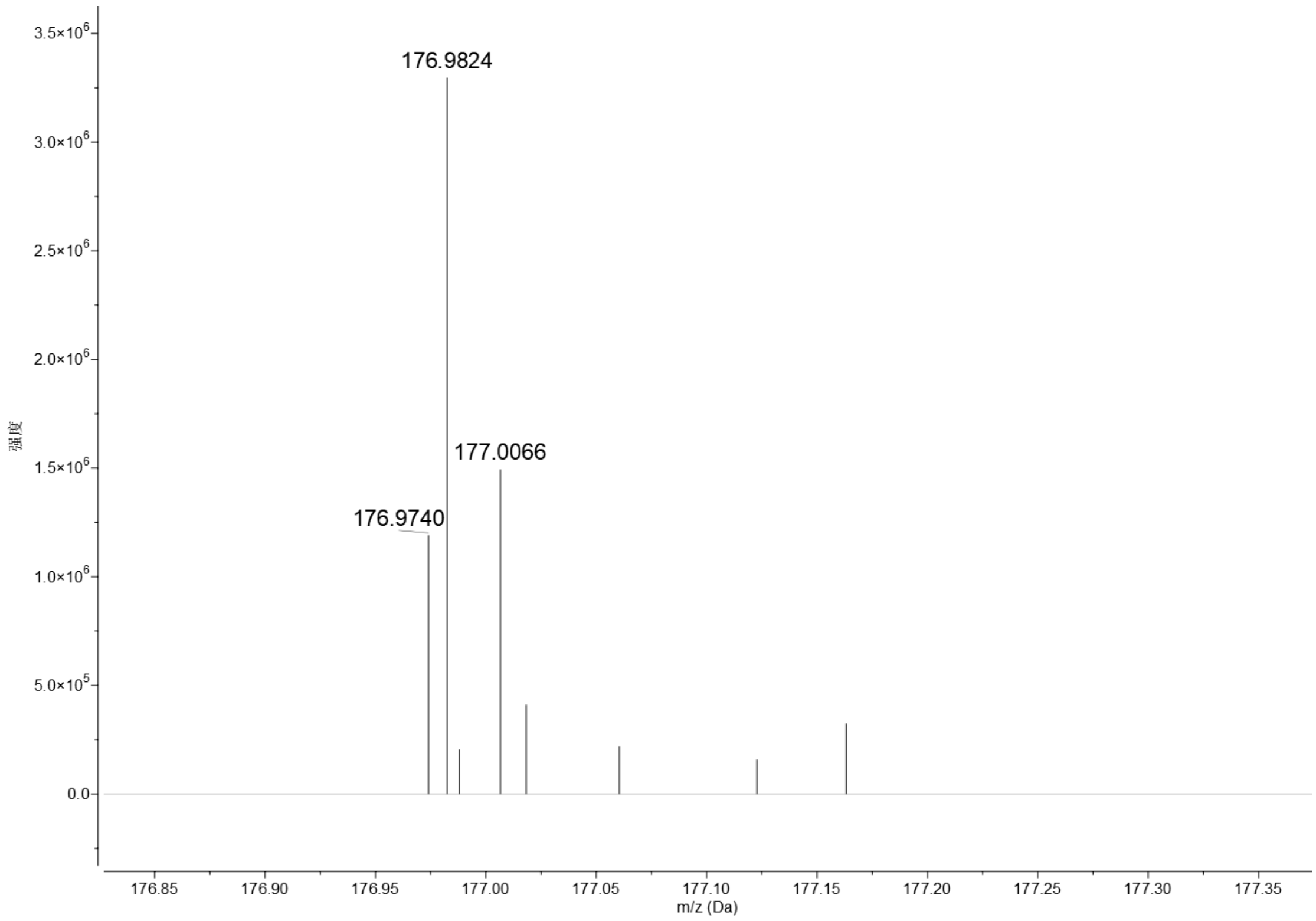

Figure S47 HRMS spectrum of compound **1**

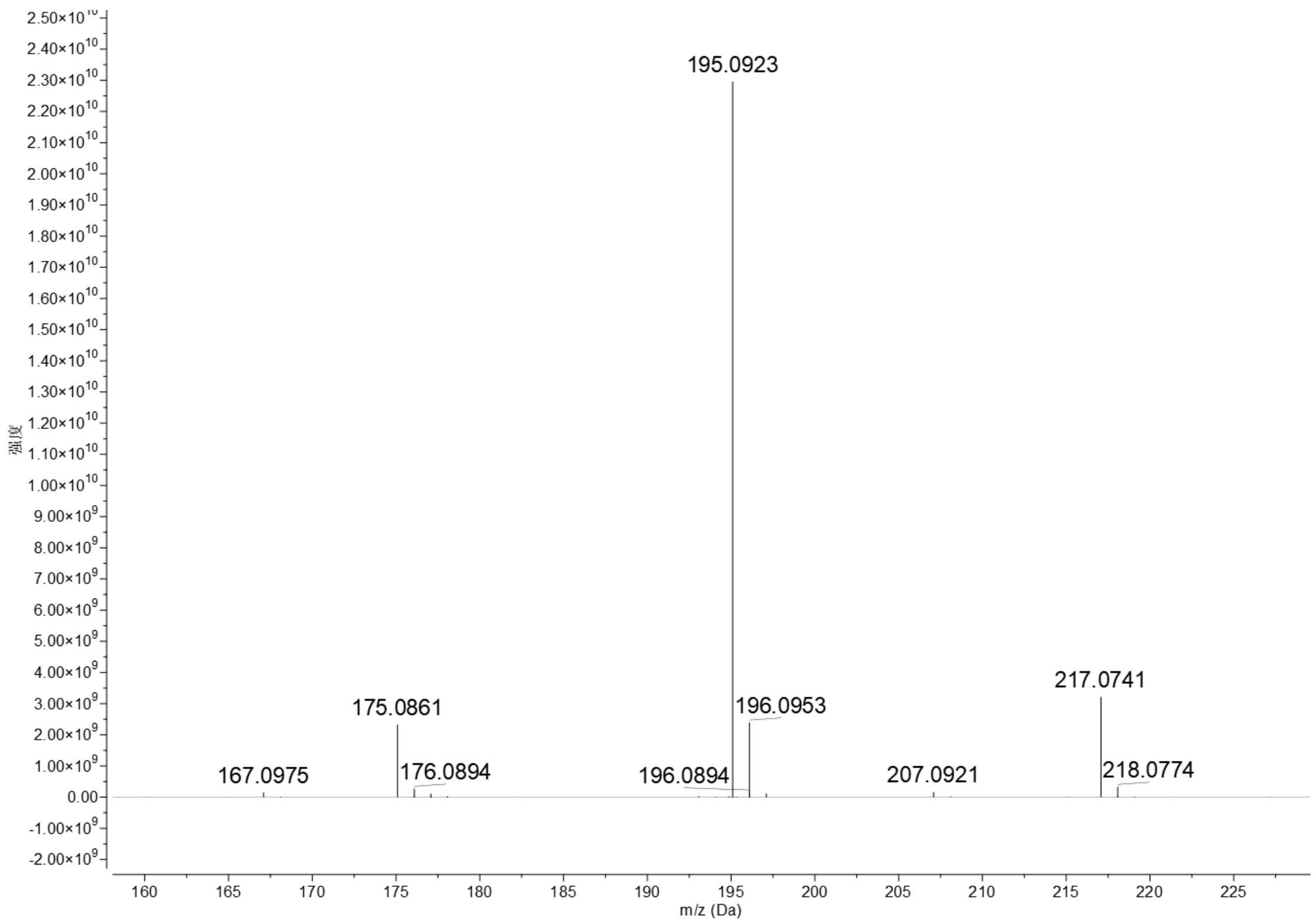

Figure S48 HRMS spectrum of compound **2**

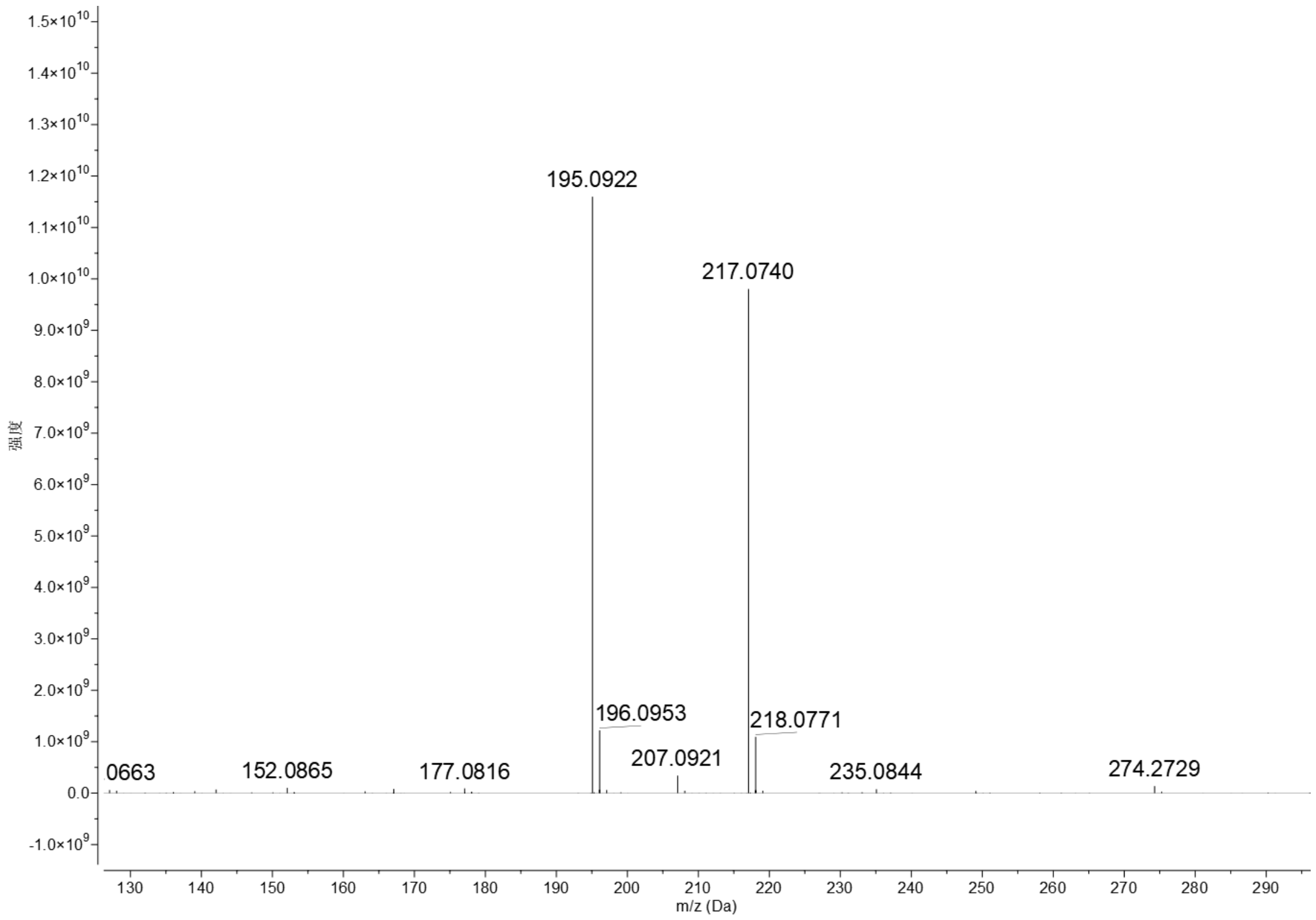

Figure S49 HRMS spectrum of compound **3**

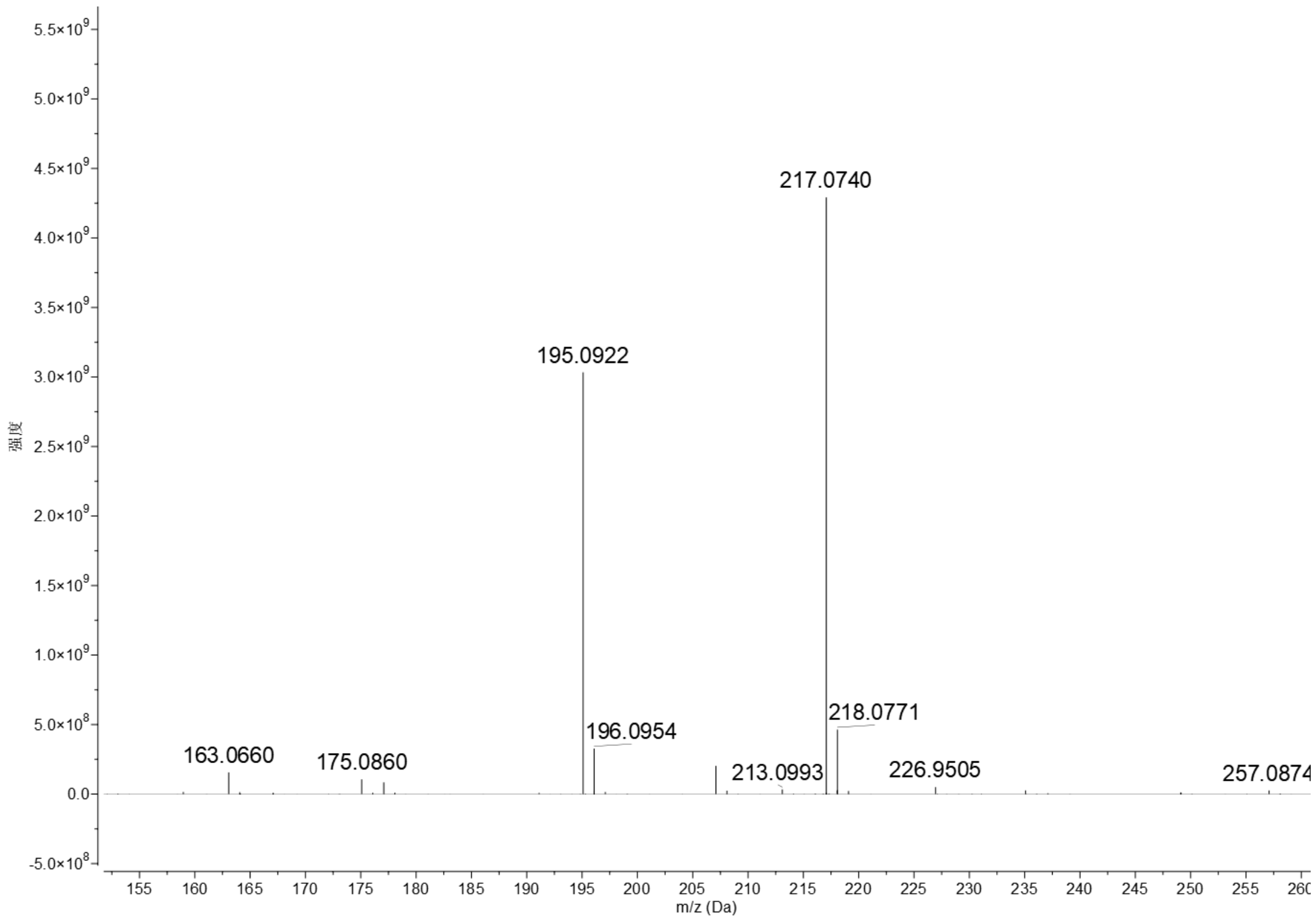

Figure S50 HRMS spectrum of compound **4**

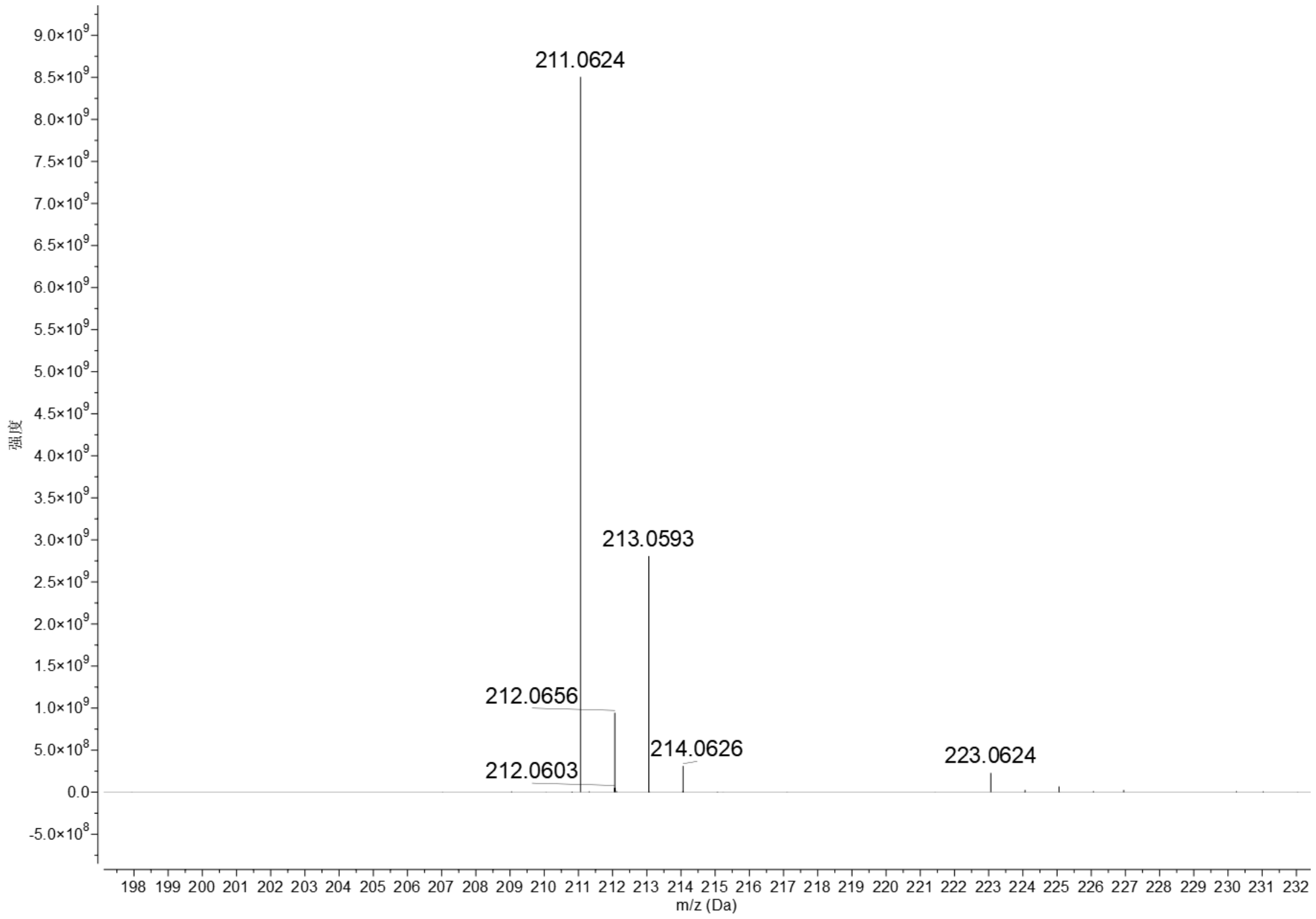

Figure S51 HRMS spectrum of compound **5**

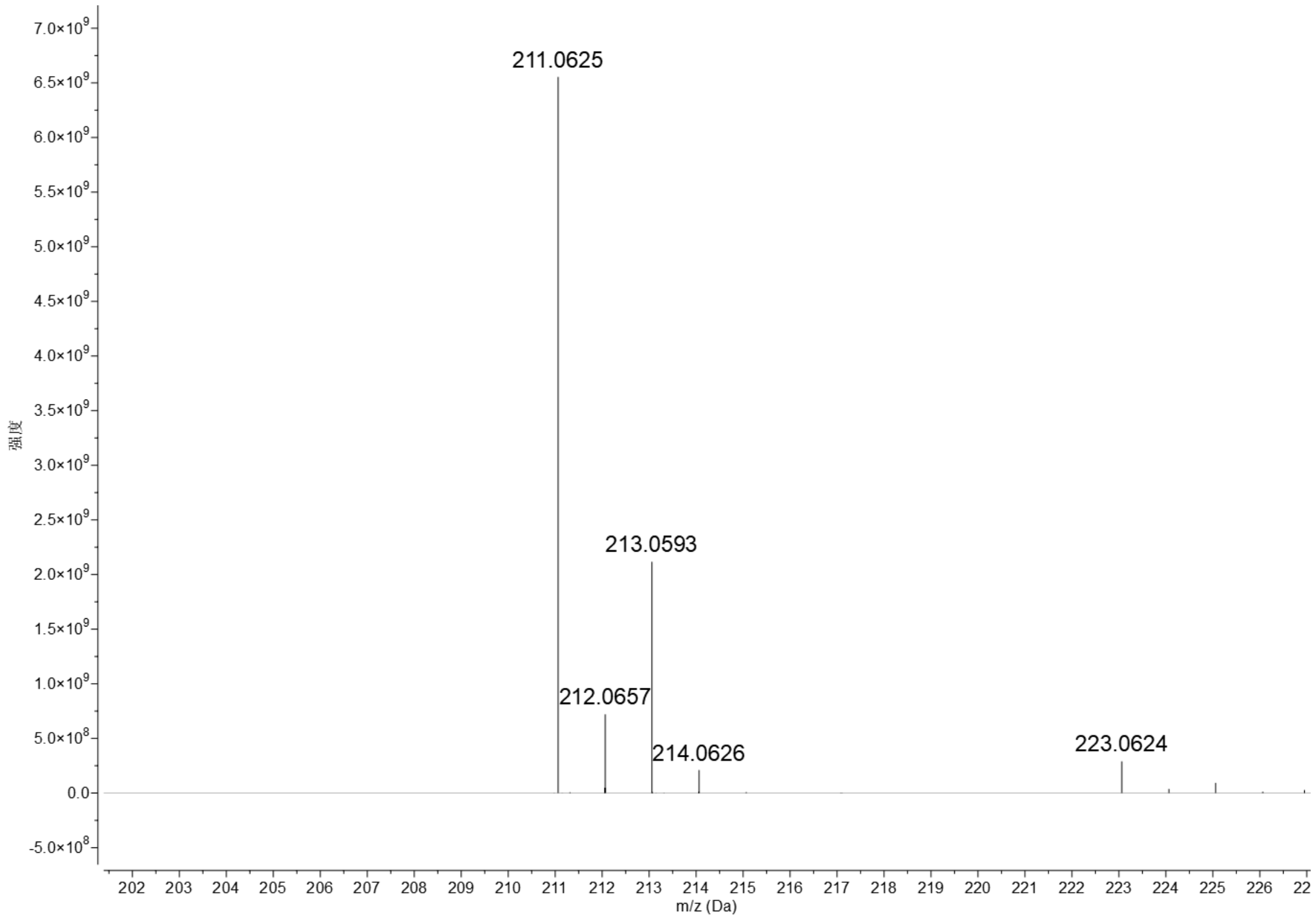

Figure S52 HRMS spectrum of compound **6**

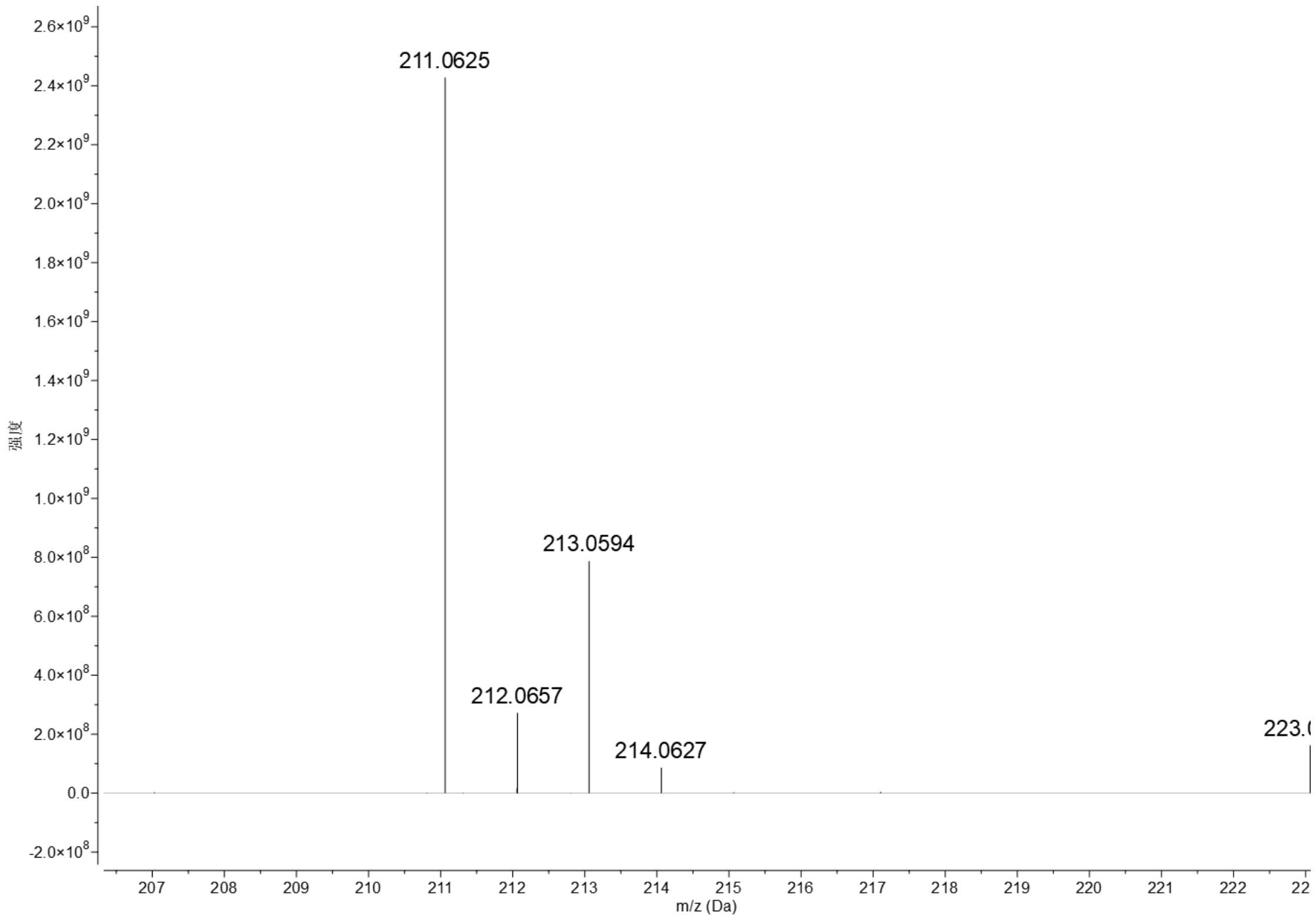

Figure S53 HRMS spectrum of compound **7**

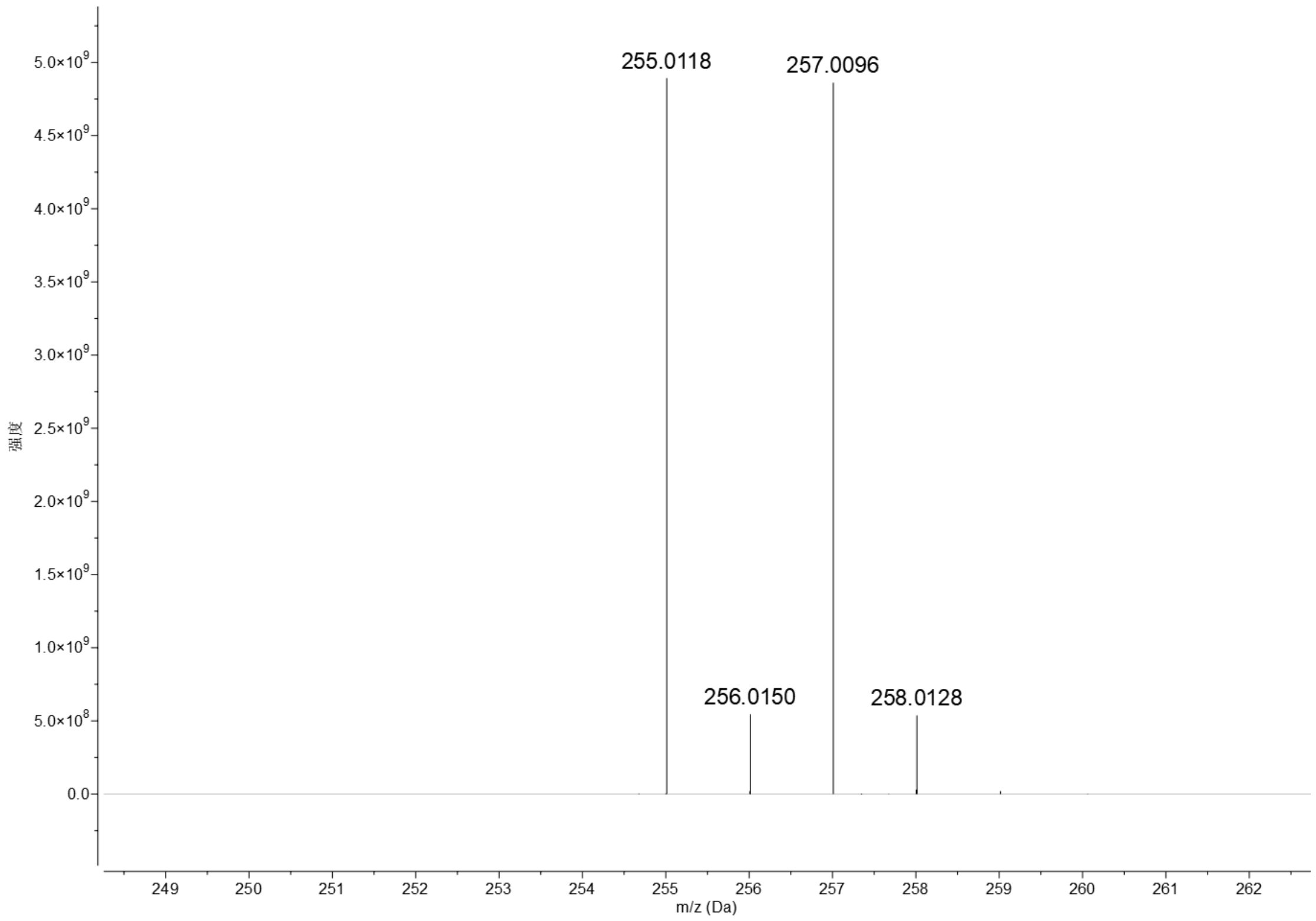

Figure S54 HRMS spectrum of compound **8**

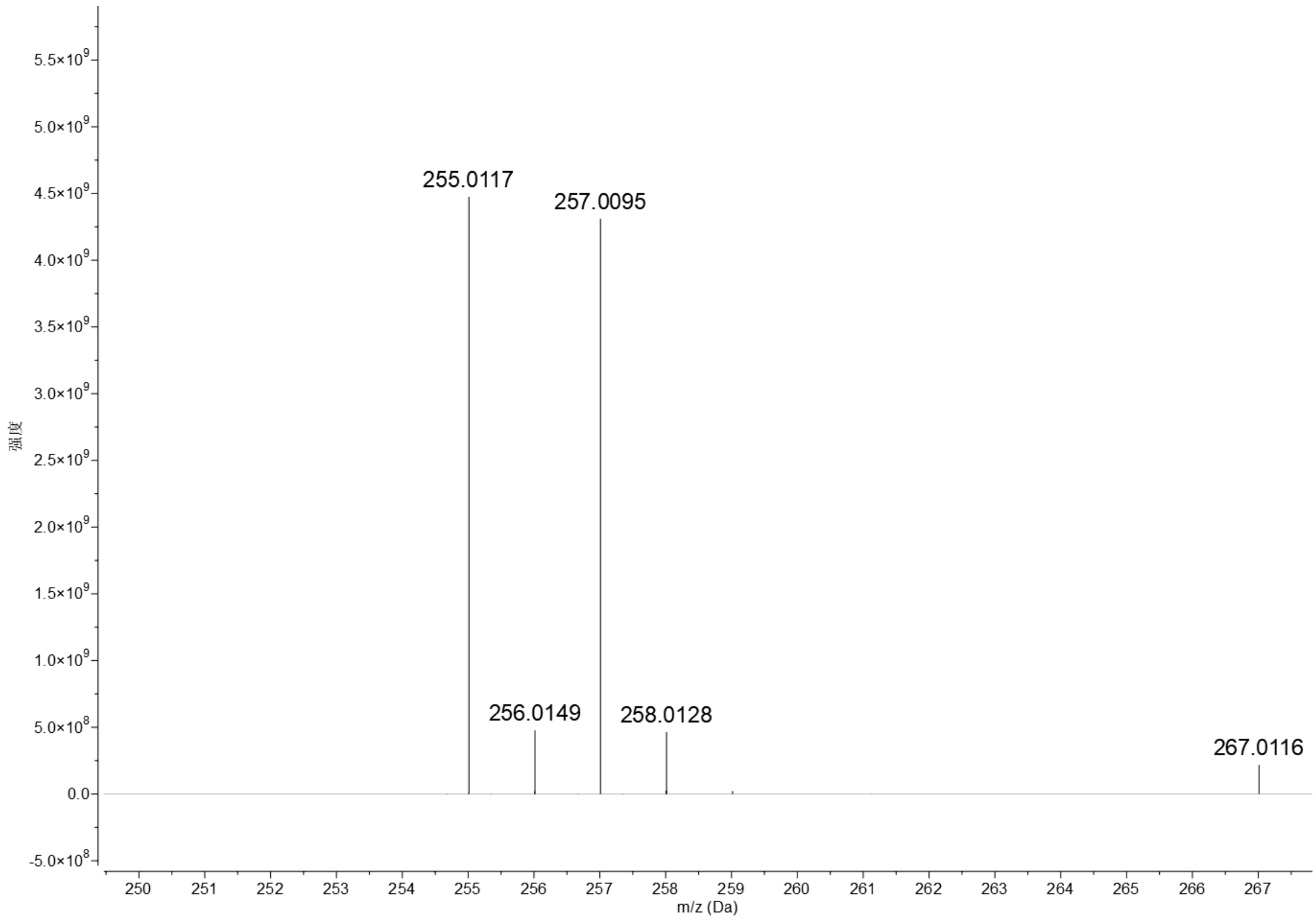

Figure S55 HRMS spectrum of compound **9**

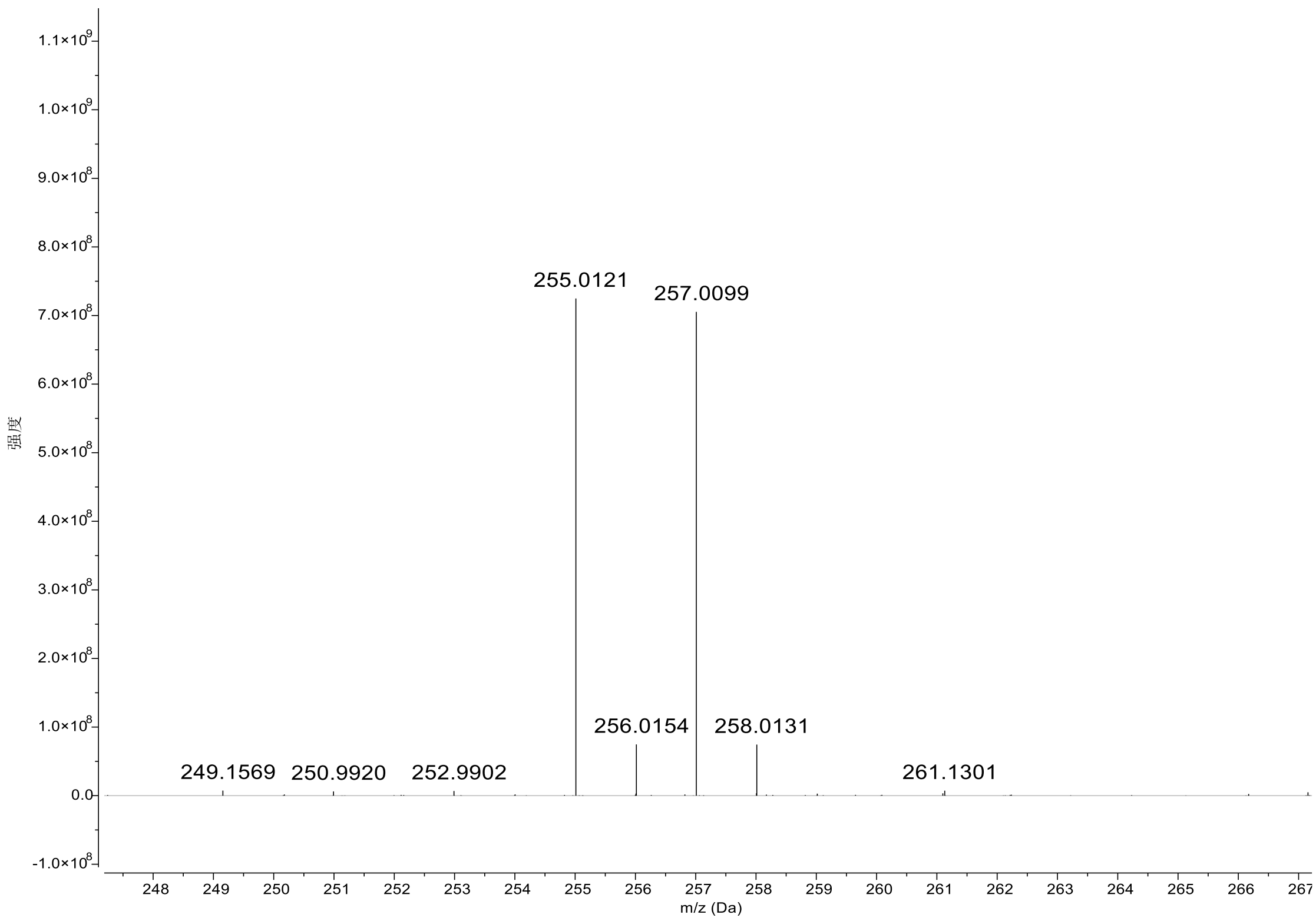

Figure S56 HRMS spectrum of compound **10**

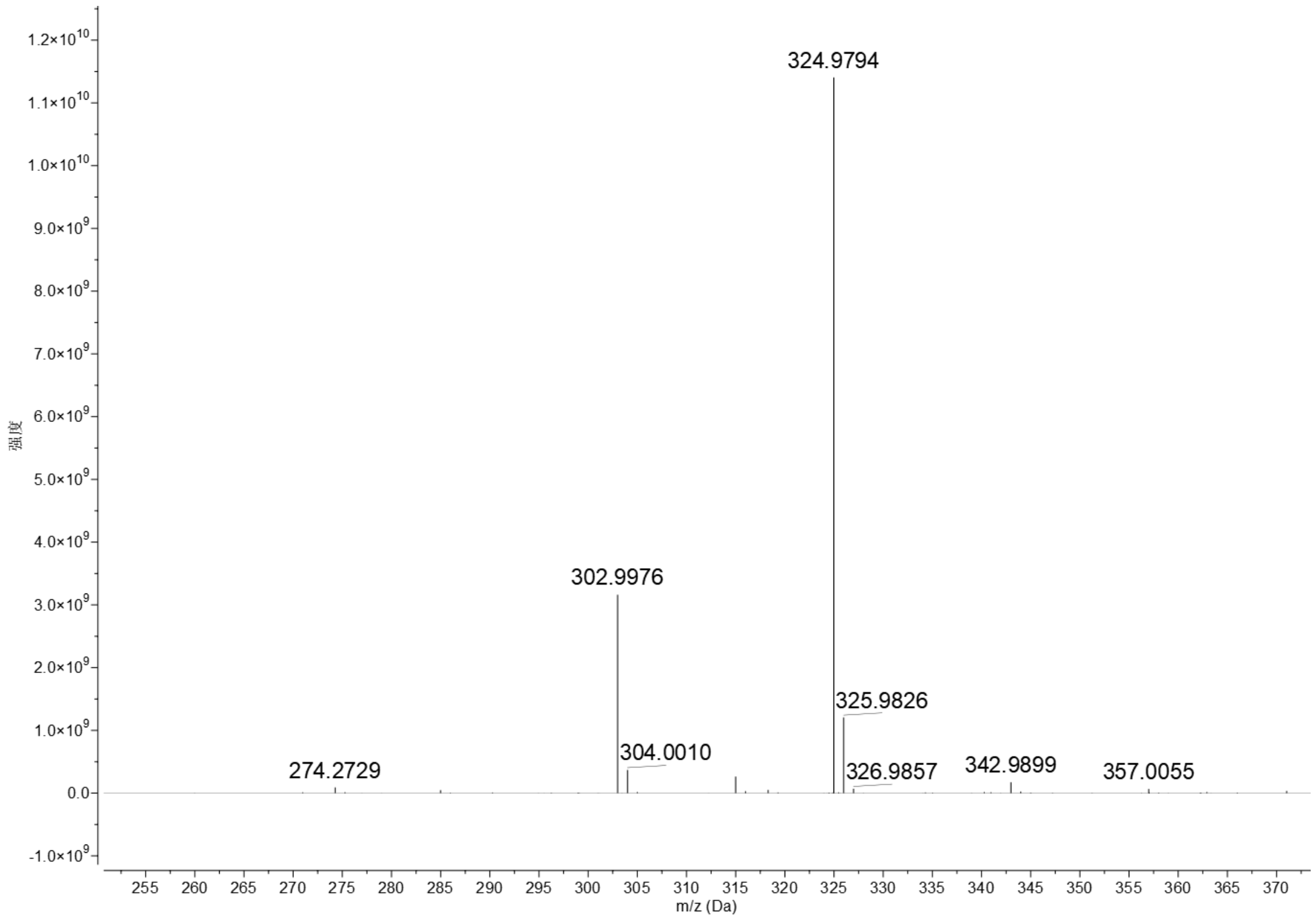

Figure S57 HRMS spectrum of compound **11**

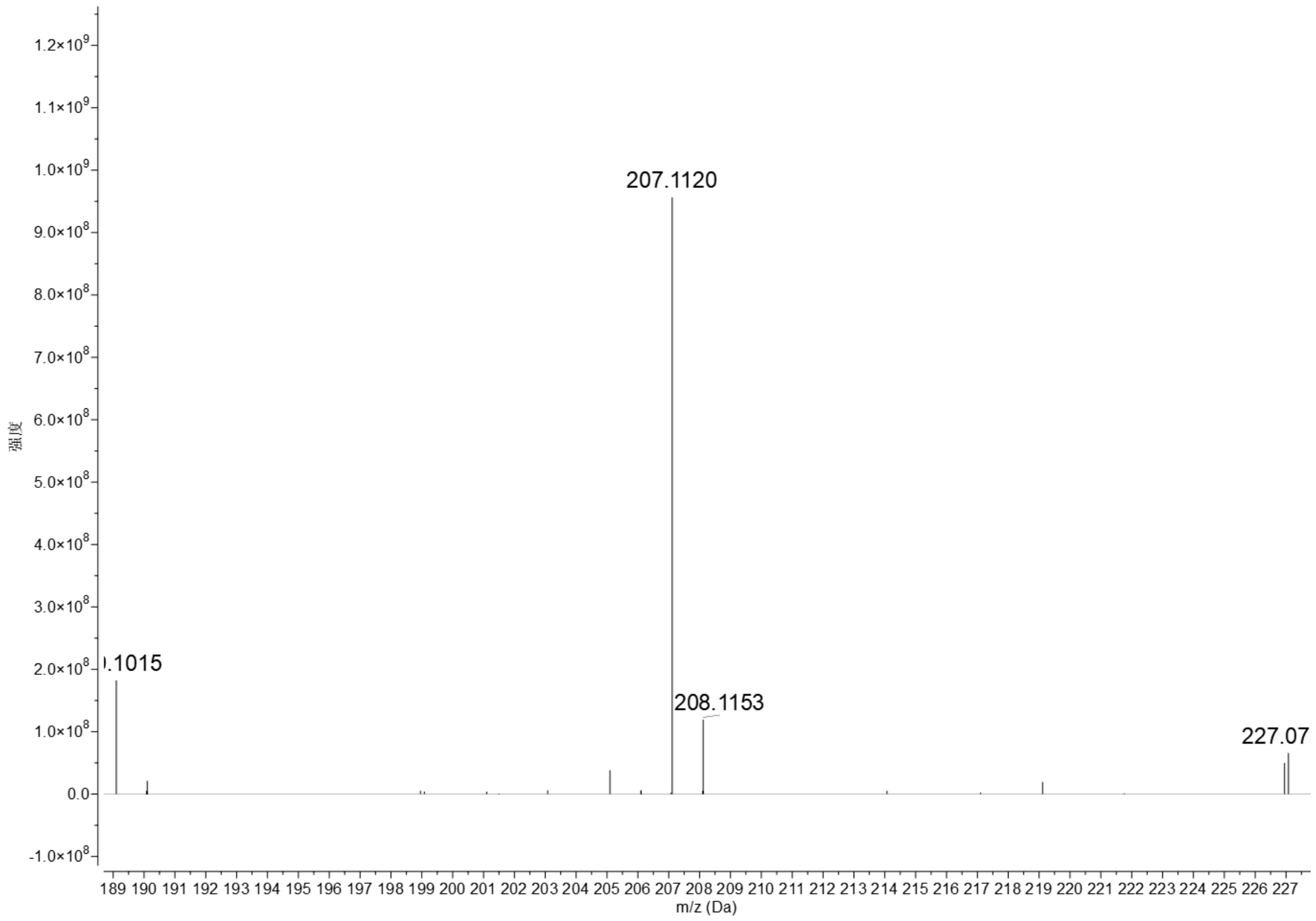

Figure S58 HRMS spectrum of compound **12**

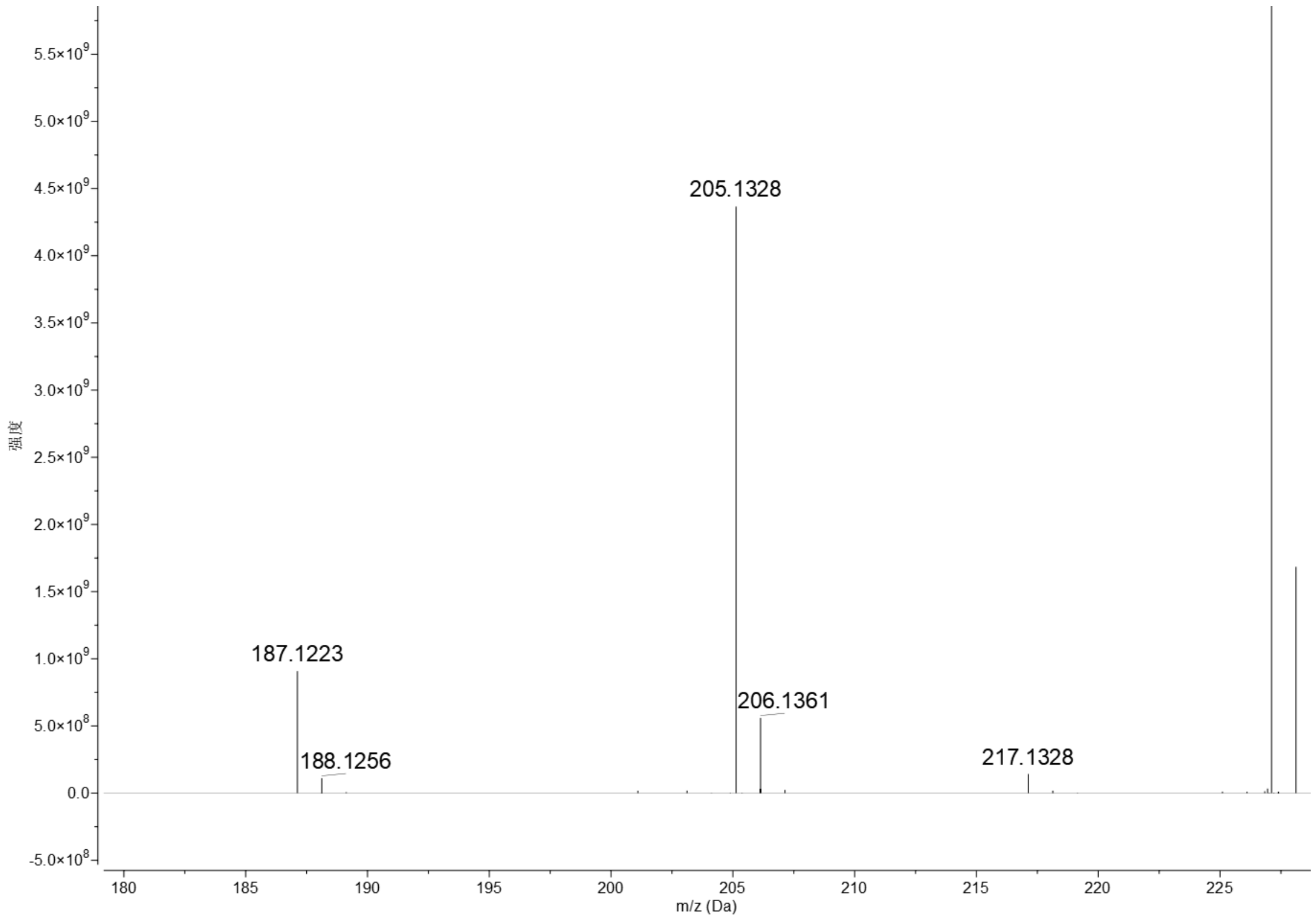

Figure S59 HRMS spectrum of compound **13**

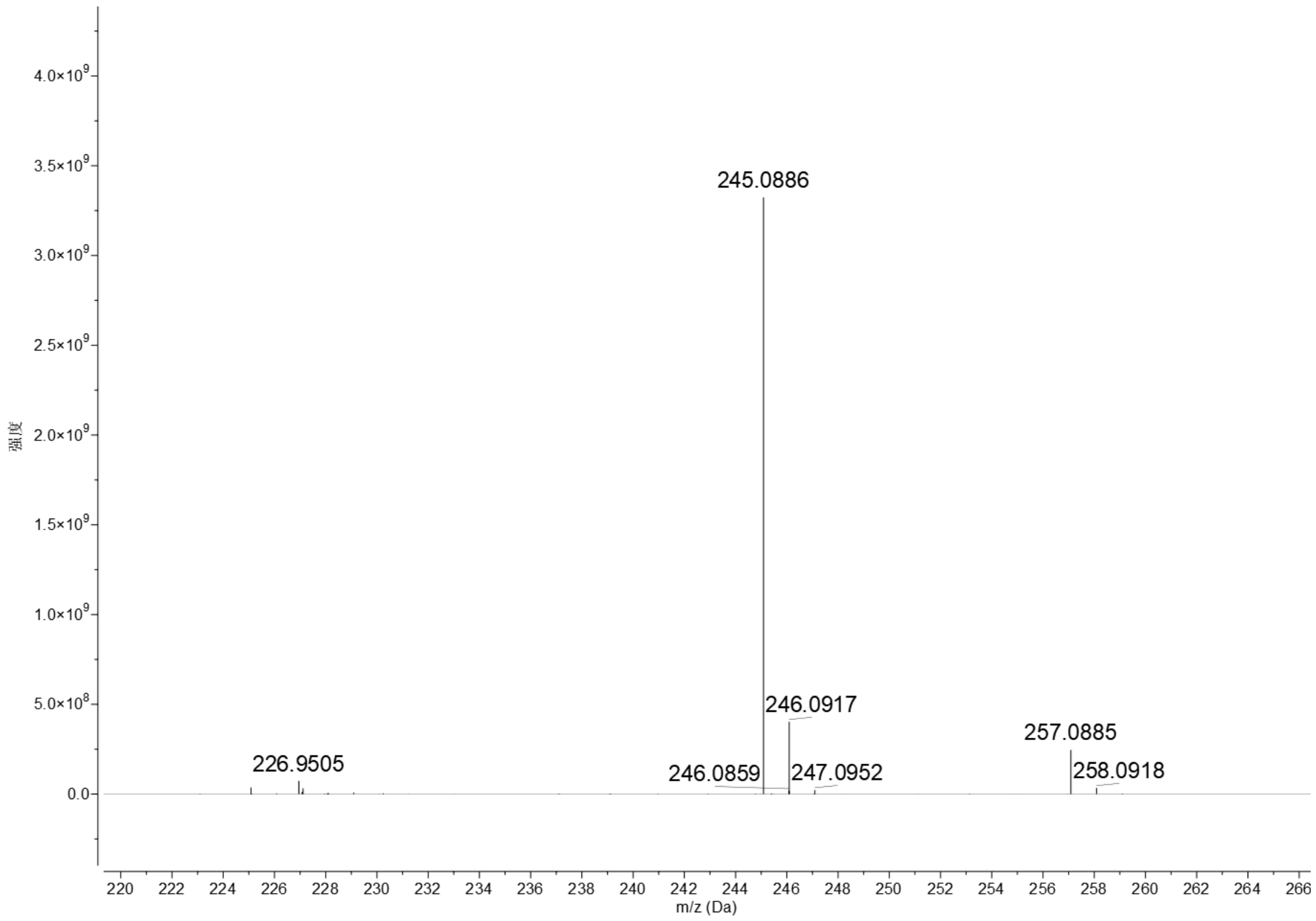

Figure S60 HRMS spectrum of compound **14**

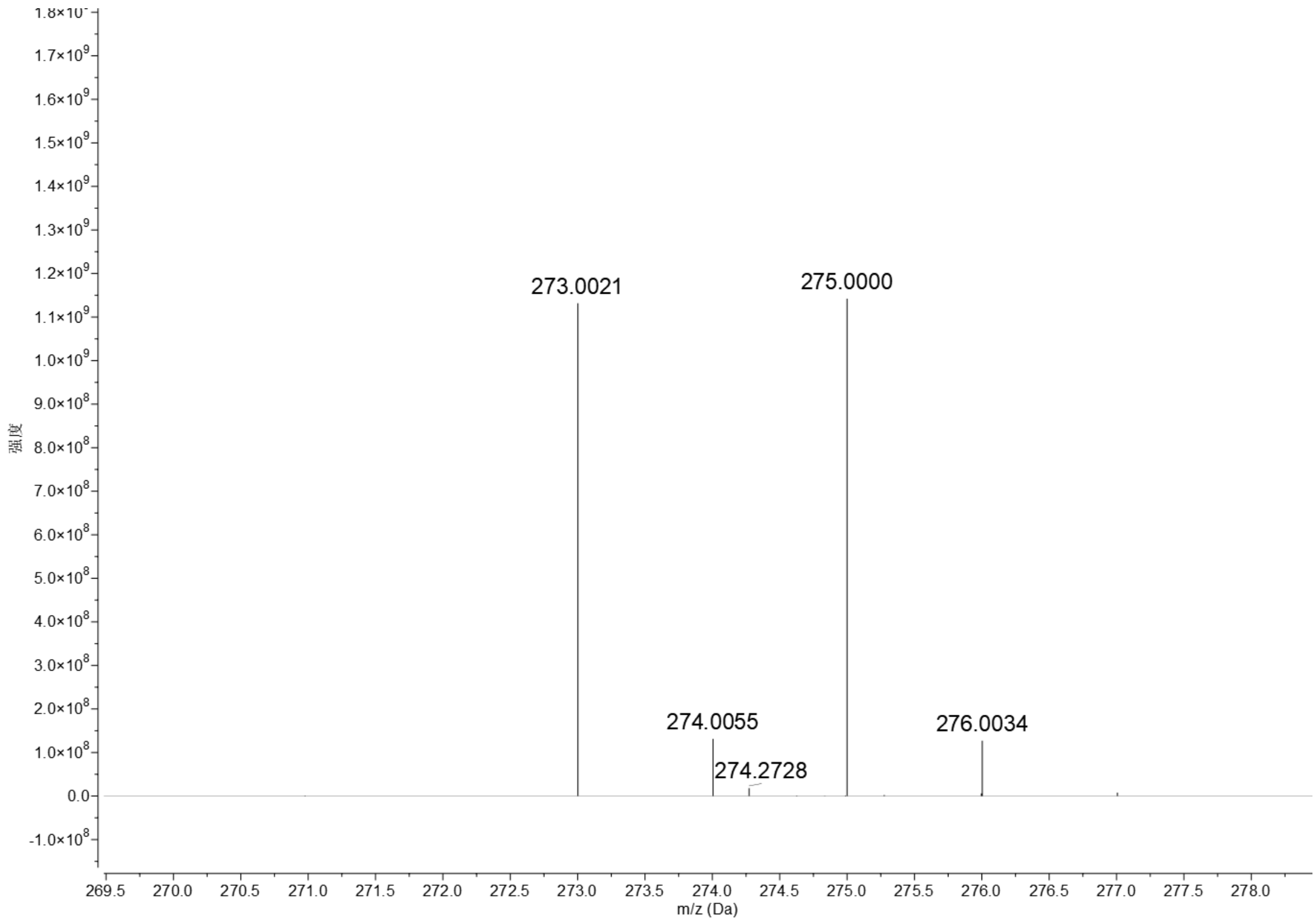

Figure S61 HRMS spectrum of compound **15**

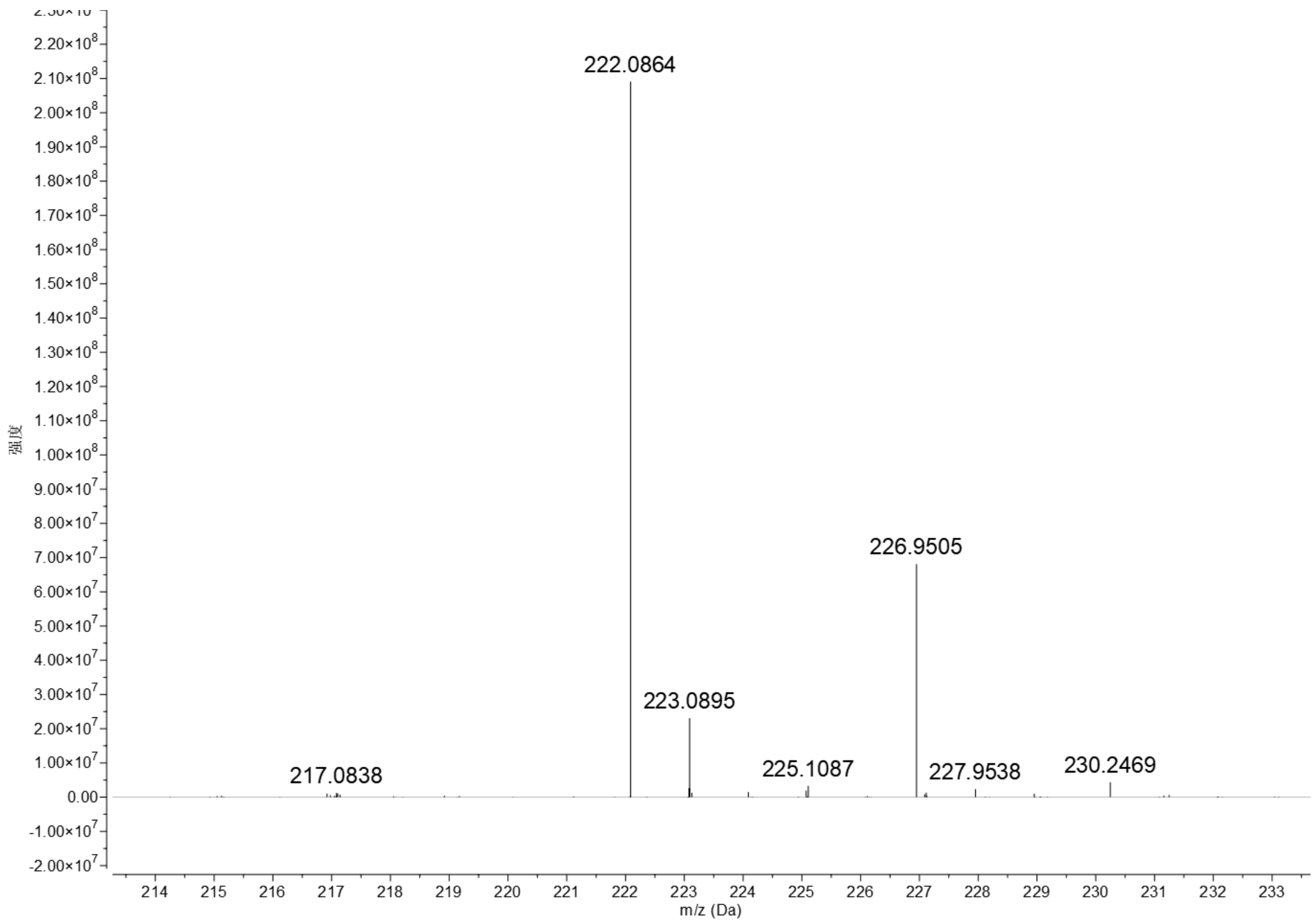

Figure S62 HRMS spectrum of compound **16**

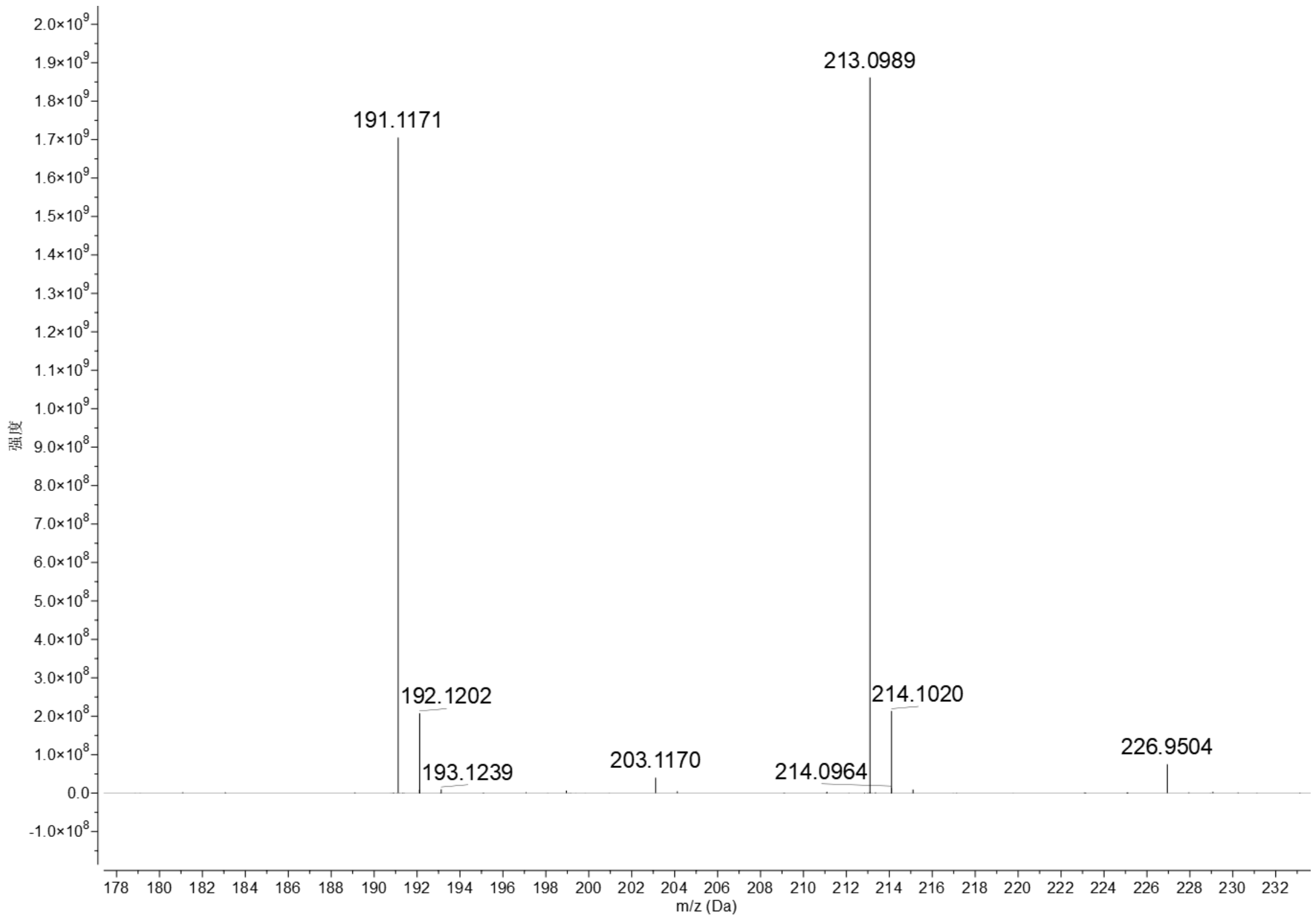

Figure S63 HRMS spectrum of compound **17**

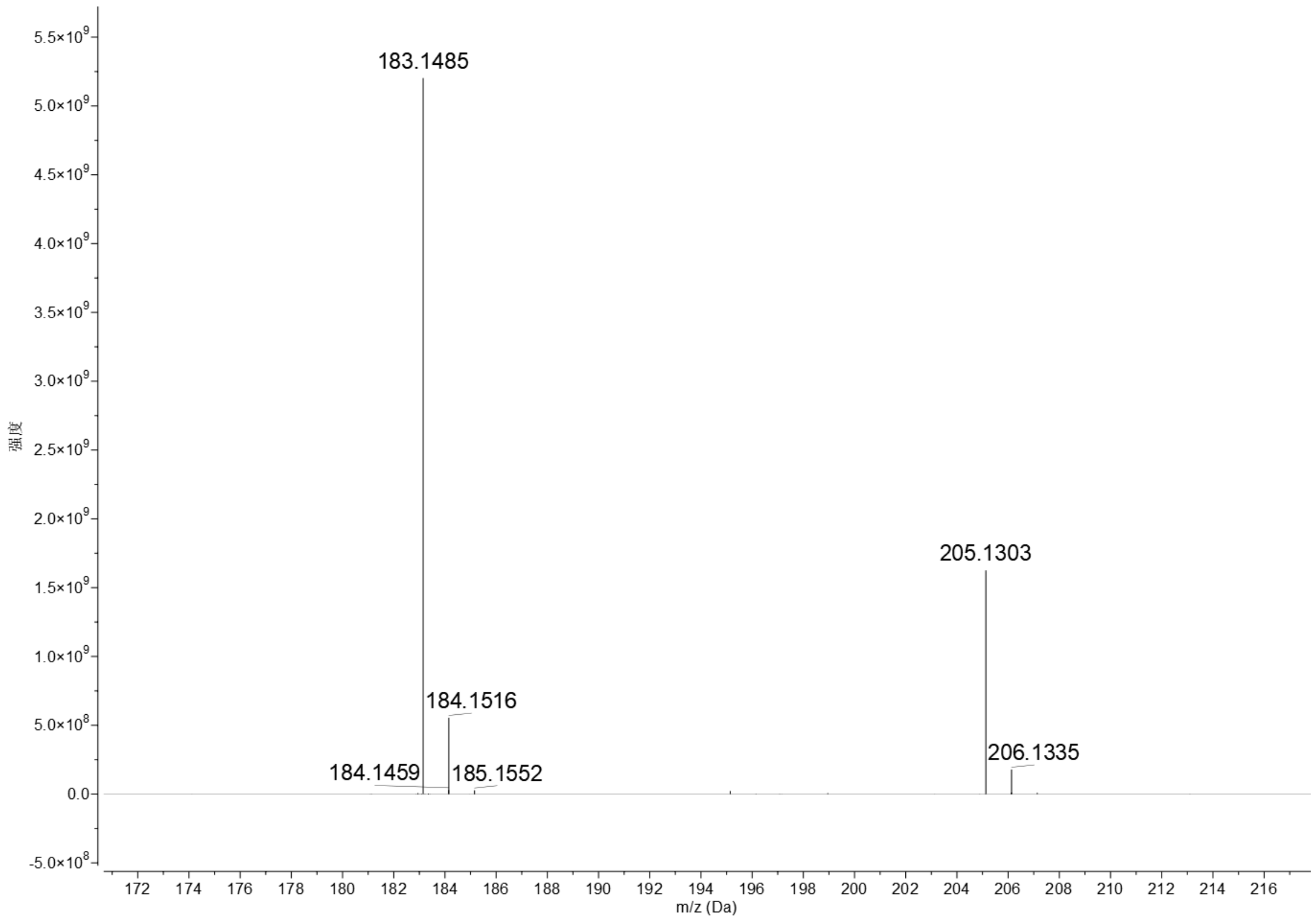

Figure S64 HRMS spectrum of compound **18**

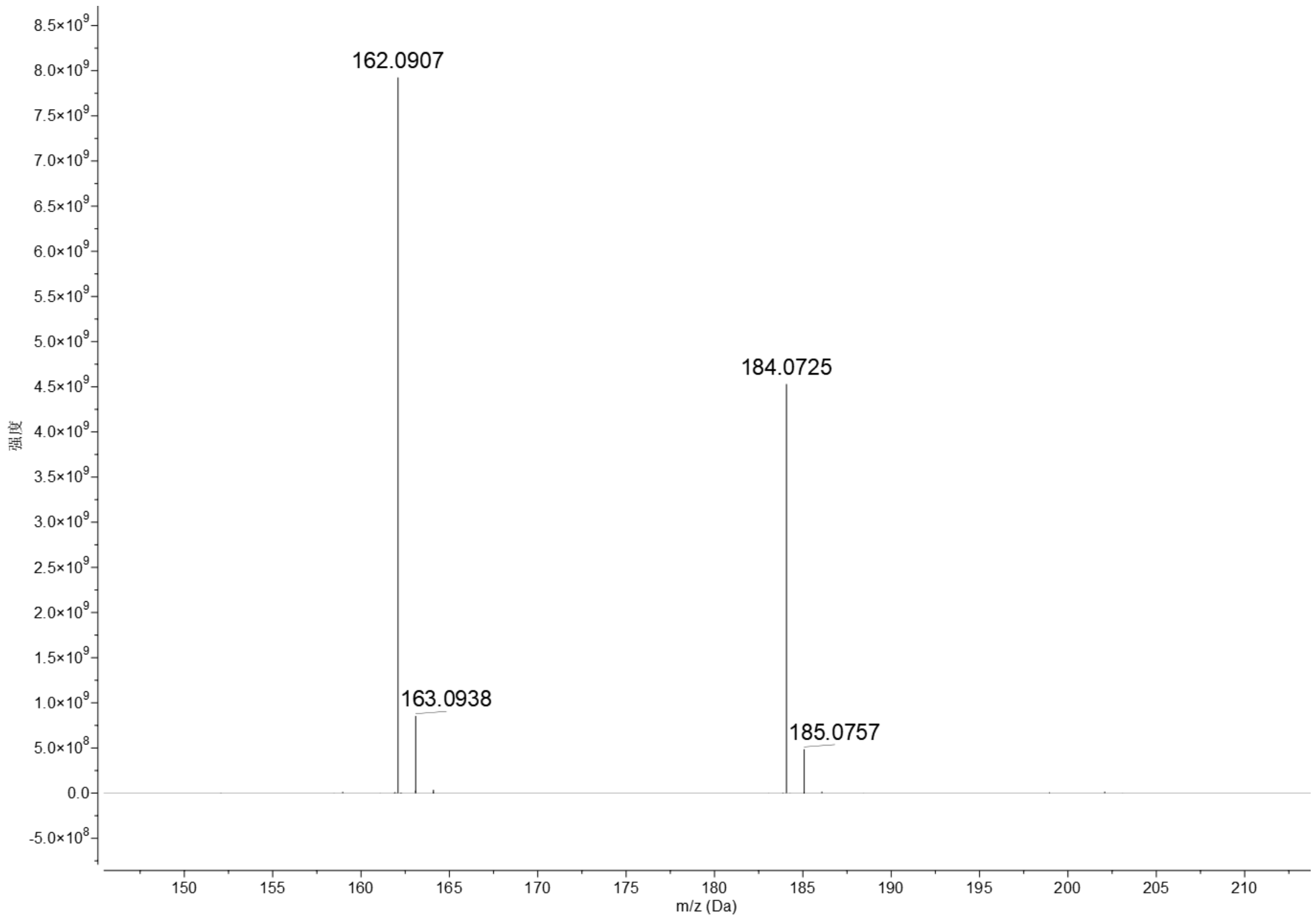

Figure S65 HRMS spectrum of compound **19**

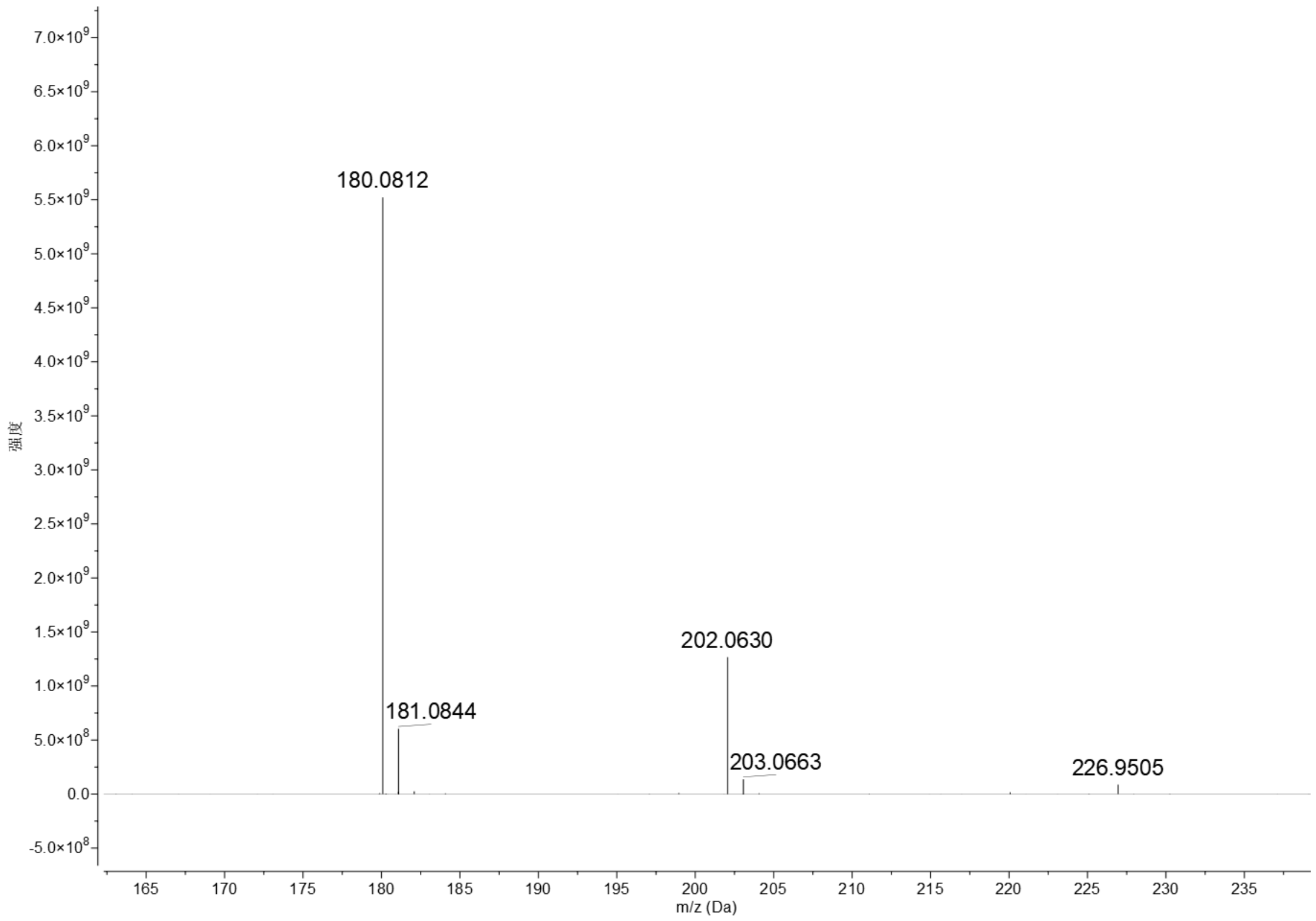

Figure S66 HRMS spectrum of compound **20**

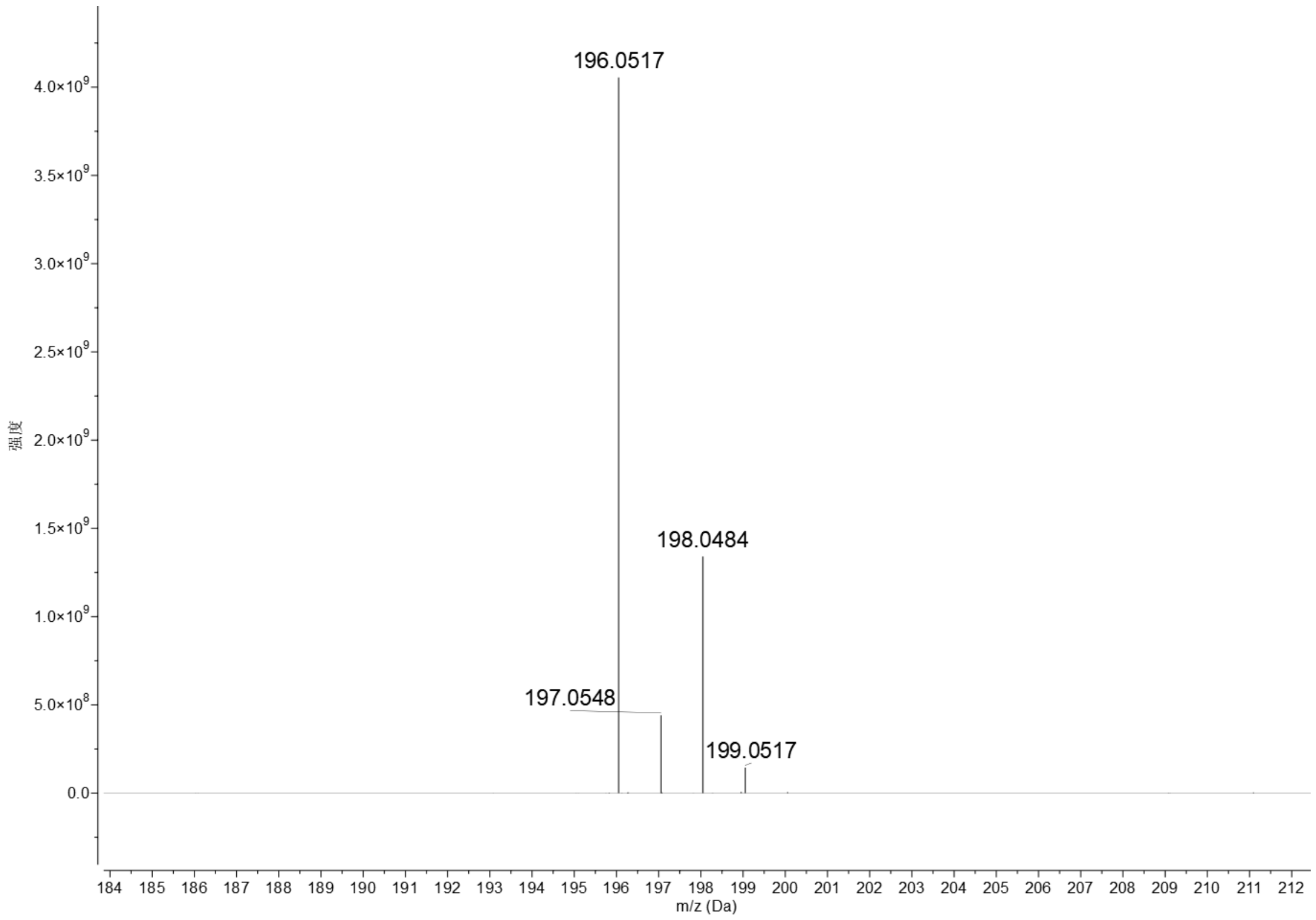

Figure S67 HRMS spectrum of compound **21**

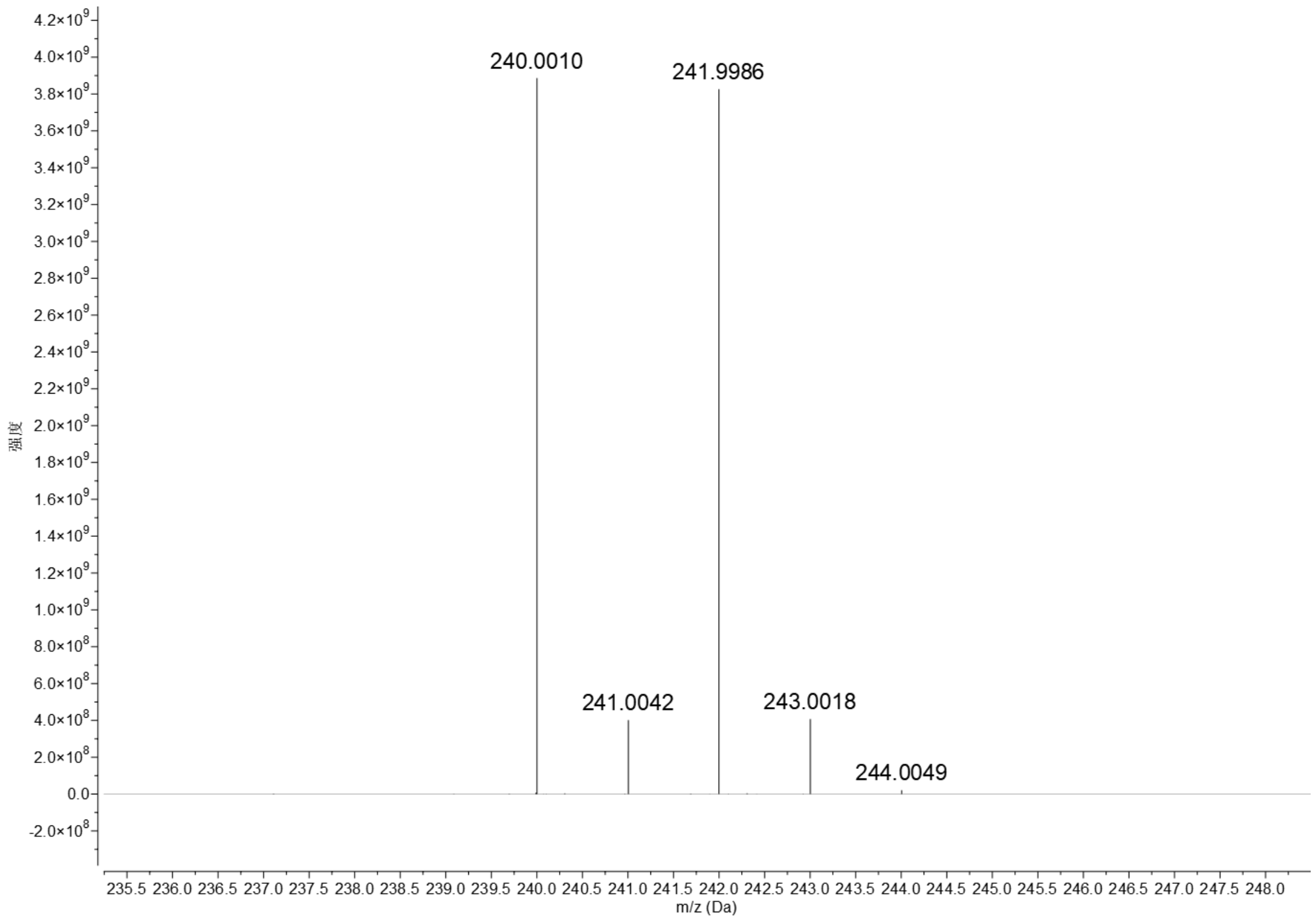

Figure S68 HRMS spectrum of compound **22**

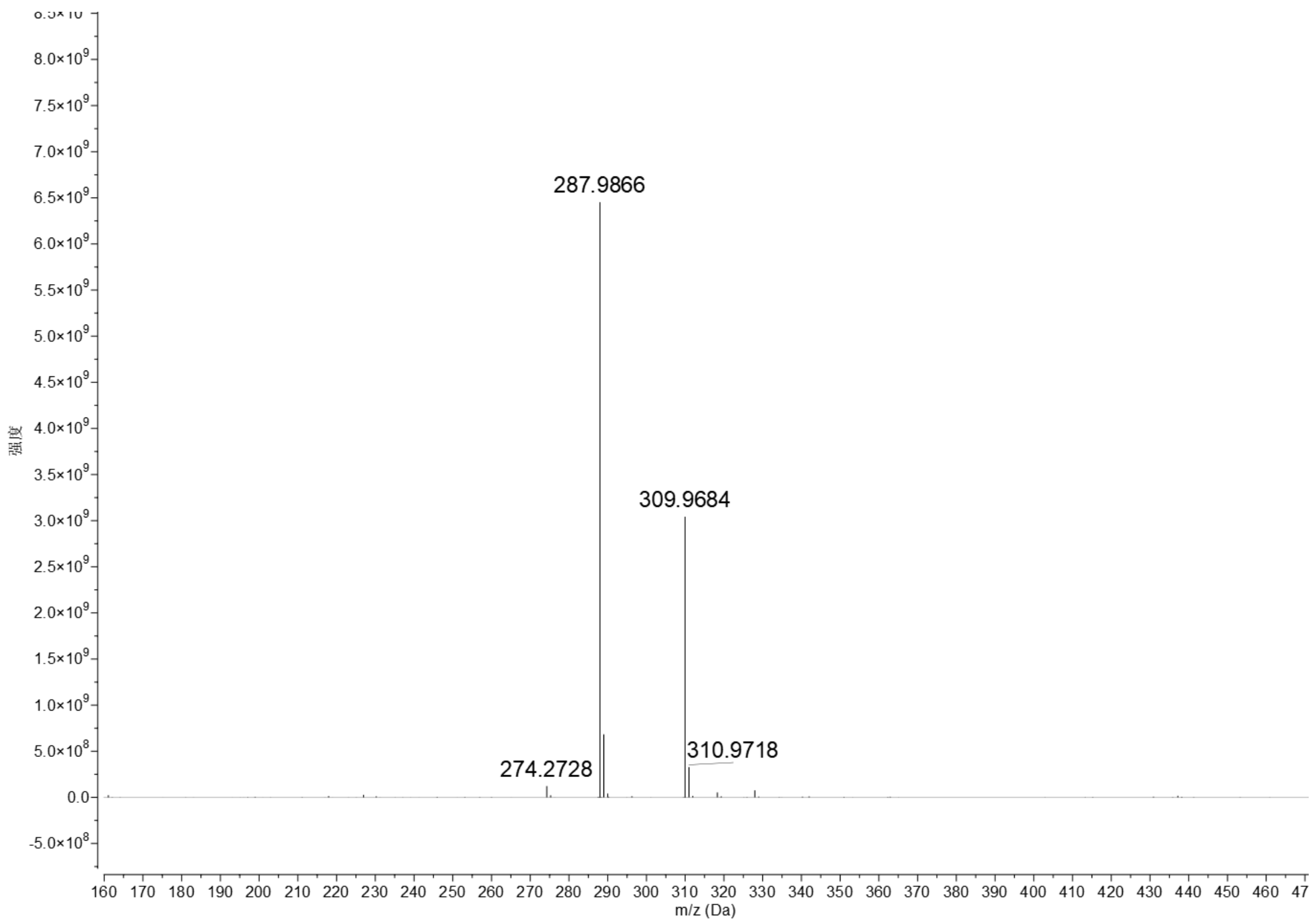

Figure S69 HRMS spectrum of compound **23**
